# Automatically listing senior members of departments as co-authors is highly prevalent in health sciences: meta-analysis of survey research

**DOI:** 10.1038/s41598-024-55966-x

**Published:** 2024-03-11

**Authors:** Reint A. Meursinge Reynders, Davide Cavagnetto, Gerben ter Riet, Nicola Di Girolamo, Mario Malički

**Affiliations:** 1 Department of Oral and Maxillofacial Surgery, Amsterdam University Medical Center (Amsterdam UMC) Location AMC, Meibergdreef 9, 1105 AZ Amsterdam, The Netherlands; 2 Studio di Ortodonzia, Via Matteo Bandello 15, 20123 Milan, Italy; 3 Faculty of Health, Urban Vitality Centre of Expertise, Amsterdam University of Applied Sciences, Amsterdam, The Netherlands; 4 Department of Cardiology, Amsterdam University Medical Center (Amsterdam UMC) Location AMC, Meibergdreef 9, 1105 AZ Amsterdam, The Netherlands; 5 Department of Clinical Sciences, Cornell University, 930 Campus Rd, Ithaca, NY 14853 USA; 6 EBMVet, Via Sigismondo Trecchi 20, 26100 Cremona, CR Italy; 7 Stanford Program on Research Rigor and Reproducibility (SPORR), Stanford University, Stanford, CA USA; 8 Department of Epidemiology and Population Health, Stanford University, Stanford, CA USA; 9 Meta-Research Innovation Center at Stanford (METRICS), Stanford University, Stanford, CA USA

**Keywords:** Health care, Medical research

## Abstract

A systematic review with meta-analysis was conducted to assess the prevalence of automatically listing (a) senior member(s) of a department as co-author(s) on all submitted articles in health sciences and the prevalence of degrees of support on a 5-point justification scale. Survey research was searched in PubMed, Lens.org, and Dimensions.ai. until January 5 2023. We assessed the methodological quality of studies and conducted quantitative syntheses. We identified 15 eligible surveys, that provided 67 results, all of which were rated as having low quality. A pooled estimate of 20% [95% CI 16–25] (10 surveys, 3619 respondents) of researchers in various health sciences reported that a senior member of their department was automatically listed as an author on all submitted articles. Furthermore, 28% [95% CI 22–34] of researchers (10 surveys, 2180 respondents) felt that this practice was ‘never’, 24% [95% CI 22–27] ‘rarely’, 25% [95% CI 23–28] ‘sometimes’, 13% [95% CI 9–17] ‘most of the time’, and 8% [95% CI 6–9] ‘always justified’. The practice of automatically assigning senior members of departments as co-authors on all submitted manuscripts may be common in the health sciences; with those admitting to this practice finding it unjustified in most cases.

*Registration of the protocol* The protocol was registered in Open Science Framework. Link: https://osf.io/4eywp/.

## Introduction

The practice of listing a senior member(s) of a department, who did not qualify for authorship, as a co-author on all or most submitted articles can be an efficient way to boost the scientific output of these individuals. This practice sets a poor role model, dilutes the input of those who really did the work and can also mislead other researchers, practicing physicians, policymakers, and the public^1^. This systematic review of survey research assessed the prevalence of this practice in research departments of health sciences and the prevalence of degrees of support.

Honorary authorship (HA) refers to authorship assigned to individuals that should not have been included as authors of a publication, because they made no or insufficient contributions to qualify as authors^2^. The International Committee of Medical Journal Editors (ICMJE) has defined a series of criteria for authorship, which are commonly used in publications in health sciences^3^. Not fulfilling one or more of these criteria has been defined as ICMJE-based HA. The prevalence of HA is commonly measured as perceived HA or ICMJE-based HA^3–6^. The practice of automatically listing senior members of a department as co-authors on all or most submitted articles from these departments when such members did not qualify for authorship can be considered as a form of serial honorary authorship. This practice has several consequences: (1) the mass production of honorary authors, (2) the facilitation of the publication of manuscripts, especially when the senior members are well known researchers and when editors and peer-reviewers are not blinded to the authors’ identities, (3) propagation of inequality and unfairness, (4) violation of the principle that an academic author has made an important intellectual contribution to a scholarly work, (5) undermining of scientific integrity in that readers might assume that the research is more credible than it actually is, based on the reputation of honorary author(s), and (6) erosion of accountability for the work.

Although a few studies assessed this practice in specific fields^6–8^, insight into the magnitude of this practice in the health sciences more broadly is missing. The objective of this study was to assess, in the health sciences, the prevalence of:

1. Researchers reporting the practice of listing (a) senior member(s) of a department, who did not qualify for authorship, as co-author(s) on all or most submitted articles by default (Review item 1).
2. Researchers reporting the practice of automatically listing (a) senior member(s) of a department as a co-author(s) on all submitted articles (Review item 2).
3. Degree of support for the practice reported under review item 2 (Review item 3).

## Methods

The objectives and outcomes of the protocol differed only slightly from those of a previous protocol on honorary authorship issues^2^. We assessed all outcomes that were originally planned in this new protocol. We registered this new protocol in Open Science Framework (OSF), https://osf.io/4eywp/. The Checklist of the Preferred Reporting Items for Systematic review and Meta-Analysis (PRISMA) statement^9,10^ was included with this manuscript. Additional information on our research procedures and methodological differences between the original published protocol^2^ and the completed review are given in the Appendix.

### Eligibility criteria

We included publications in health sciences which reported on survey results on a series of pre-defined items regarding the practice of listing (a) senior member(s) of a department, as (a) co-author(s) on all or most submitted articles by default. We included publications on this topic in any language, and any setting and time point. We used the same eligibility criteria as those used in our previous review on HA in health sciences^2^. Full eligibility criteria are in the Appendix (Additional item C, page 5).

### Information sources and search strategy

We searched PubMed, Lens.org, and Dimensions from inception till January 5 2023 with no language or data filters. We chose these databases as they are freely available to all researchers and their coverage has been shown to be greater than that of proprietary databases^11–13^. Only for Lens.org and Dimensions.ai we applied health sciences filters. To avoid missing eligible studies we also manually searched all references of the included surveys for additional eligible surveys. The complete strategy is presented in the Appendix (Additional item D, page 6). For the development of our search strategy, we consulted both previous systematic reviews on our research topic as well as an information specialist to help design our initial strategies. These strategies were subsequently piloted and fine-tuned and were then peer-reviewed and approved in our published protocol.

### Survey selection process and data collection

Survey selection and data collection was conducted by RMR and DC, independently. Disagreements were resolved through discussions, information provided by the contacted authors of the surveys, or through arbitration by a third reviewer (GTR). Rayyan^14^ was used first to remove duplicates and then for the initial screening of titles and abstracts. Full texts of potentially eligible manuscripts were subsequently retrieved and assessed. References of included surveys were also assessed for eligibility. We implemented Cochrane’s strategies^15^ to identify multiple reports from the same study and checked eligible surveys for retractions, possible scientific misconduct, or for published corrections, errata or comments. Excluded full-text articles and the rationale for their exclusion are given in the Appendix (Additional item K, pages 30–33). All data items to extract were defined in our data extraction forms (Appendix, Additional item E, pages 7–12).

### Study risk of bias assessment

We used a critical appraisal tool tailored to our review to assess how the non-implementation of specific quality safeguards could have affected each eligible result of each survey. This tool consists of a 14 items pilot-tested checklist^2^. Congruent with the AMSTAR-2 tool^16^, 7 of the 14 items were labeled as ‘critical’. We adopted the AMSTAR-2 ratings high’, ‘moderate’, ‘low’, and ‘critically low’ to rate the overall confidence in each result of each eligible survey. These ratings were reported in tables together with the prevalence of yes, no, and unclear answers to each question of our critical appraisal tool. All assessments and ratings were conducted by RMR and DC, independently. In the case of disagreements, we implemented the same strategies as reported for the study selection and data collection procedures. The 14-item checklist of our quality assessment tool with user’s instructions is given in the protocol^2^ and in the Appendix (Additional item F, pages 13–17).

### Occurrence measures and synthesis methods

The prevalence was the occurrence measure used both in the presentation of single outcomes as well as in the quantitative syntheses. These proportions are reported with their exact (Wilson) 95% confidence intervals. All outcomes for the three review items were defined in the Appendix (Additional item G, page 18) with the pertinent numerators and denominators. We also reported the various response rates measured.

A narrative systematic synthesis was first conducted for all outcomes. Based on the criteria delineated in the Appendix (Additional item G, page 19) and in our established protocol, we refrained from conducting certain meta-analyses^2^. When conducting quantitative syntheses, proportions are presented in forest plots with 95% confidence intervals. The meta-analyses were done using metaprop command in Stata 18^17^. Random-effects models were used, because we expected between-survey variance. To address unit-of-analysis issues we checked whether the same surveyees participated more than once in the same survey. To address missing data issues, we contacted either the corresponding authors or those involved in the statistical analysis by email and sent them reminders after one and two weeks. The data were labelled as missing when after 2 weeks no data were received.

### Investigation of heterogeneity and sensitivity analyses

The presence and extent of heterogeneity was inspected visually by assessing the overlap of the confidence intervals in the forest plot, by conducting the test of homogeneity (Chi^2^), and by calculating tau^2^ i.e., the estimate of between study variance, and by calculating *I*^2^ to assess the inconsistency in the results of the surveys^18^. We explored heterogeneity through meta-regression and subgroup analyses of a series of survey-and methodology-related explanatory variables defined in our protocol^2^. Additional information on our methods to explore possible causes of heterogeneity among study results and sensitivity analyses to assess the robustness of pooled results are reported in the Appendix (Additional item G, pages 18–20).

### (Non)-reporting bias assessment

We used the term non-reporting bias which is the preferred term suggested by Cochrane^19^ and adopted the various strategies according to Cochrane to address these biases (Appendix, Additional item H, page 21). Tests for funnel plot asymmetry were not conducted, because there is no evidence that proportions adequately adjust for these tests^20^.

### Certainty assessment

The GRADE approach was implemented to assess the overall certainty of the body of evidence^21^. Four levels of certainty were assigned according to GRADE, i.e., ‘high’, ‘moderate’, ‘low’, and ‘very low certainty’^21^. For each outcome these ratings were presented in summary of findings tables together with the rationale for these ratings. The Appendix (Additional item I, pages 22–23) reports further guidance for grading the overall certainty of evidence.

## Results

### Study selection and study characteristics

**Figure 1:**
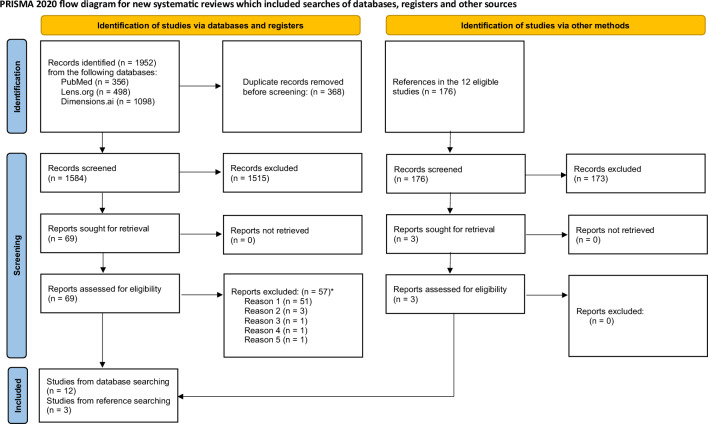
PRISMA flow diagram. *Reason for exclusion: reason 1: did not assess honorary authorship issues as defined in our protocol; reason 2: could not obtain the survey questionnaire through contacting of the authors; reason 3: non-eligible participants; reason 4: not a survey; reason 5: duplicate.

Of the 1952 records identified, 1584 remained after deduplication (Fig. 1)^9,10^. After screening 69 articles were selected for full-text assessments of which 12 were finally included in the review. Three additional articles were identified during the screening of references. This added up to a total of 15 included surveys with 15 questions on researchers reporting the practice of automatically listing a senior member(s) of a department as co-author(s) on submitted articles and 12 questions on the various justifications of this practice. These latter questions could be addressed with the following 5 justifications i.e., ‘never justified’, ‘rarely justified’, ‘sometimes justified’, ‘most of the time justified’, and ‘always justified’. A total of 67 results were obtained. All full text reports that were excluded are listed in the Appendix (Additional item K, pages 30–33) with the rationale for their non-eligibility. The questionnaires for all included surveys were either identified in the manuscripts or through contacting of authors.

Across 11 surveys that reported gender of respondents, 76.3% of respondents were male (IQR 76.3 to 80.4) (Table 1)^22^. Across 10 surveys that reported academic positions, 48.8% of respondents were associate professor or full professor (IQR 32.1 to 51.7) (Table 1). We could not reliably extract the prevalences of the continents of the respondents’ origins, because these characteristics were often not assessed or were unclear. Additional characteristics of all 15 included surveys and their characteristics are reported in the Appendix (Additional item J, pages 24–29).

**Table 1:** Characteristics of the 15 included studies.

Study/year	Response rate (%)*	Target field	Target population	Characteristics of the responding surveyees reported	Characteristics of the non-responding surveyees reported	Males among responding surveyees (%)**	Females among responding surveyees (%)**	Associate professor and higher among responding surveyees (%)
Bonekamp^23^	41.6 (490/1179) (N2)	Radiology	Corresponding authors	Yes	No	70.0 (343/490)	30.0 (147/490)	48.8 (239/490)
Eisenberg^24^	28.6 (383/1338) (N1)	Radiology	First authors	Yes	No	76.3 (299/392)	23.7 (93/392)	38.0 (149/392)
Eisenberg^25^	24.5 (328/1337) (N2)	Radiology	First authors	Yes	No	Not reported	Not reported	21.3 (23/108)
Eisenberg^4^	16.8 (309/1839) (N3)	Radiology	First authors	Yes	No	Not reported	Not reported	32.0 (73/228)
Gadjradj^26^	30.1 (354/1143) (N2)	Neurosur-gery	Corresponding authors	Yes	No	88.4 (313/354)	11.6 (41/354)	51.7 (193/373)
Gadjradj^27^	24.1 (284/1180) (N2)	Spine	Corresponding authors	Yes	No	80.4 (229/285)	19.6 (56/285)	Not assessed
Gadjradj^7^	24.7 (226/914) (N2)	Oral and maxillofa-cial surgery	Corresponding authors	Yes	No	74.9 (170/227)	25.1 (57/227)	Not assessed
Gülen^28^	54.5 (666/1221) (N3)	Cochrane reviews	First authors	Yes	No	44.6 (297/666)	54.8 (365/666)	32.1 (225/700)
Hardjosan-toso^29^	19.5 (329/11,688) (N1)	Ophtha-mology	Corresponding authors	Yes	No	Not reported	Not reported	Not assessed
Kayapa^30^	25.1 (341/1359) (N1)	Dermato-logy	Corresponding authors	Yes	No	61.4 (210/342)	38.6 (132/342)	56.2 (187/333)
Luiten^5^	29.6 (307/1037) (N3)	General surgery	Corresponding authors	Yes	No	77.4 (236/305)	22.6 (69/305)	60.1 (179/298)
Matawlie^31^	21.5 (226/1051) (N1)	Pain medicine	Mix of corresponding, first, and senior authors	Yes	No	Not reported	Not reported	Not assessed
Noruzi	38.6 (583/11,511) (N3)	Cardio-thoracic surgery	Corresponding authors	Yes	No	86.3 (505/585)	13.7 (80/585)	50.1 (293/585)
Nurmoha-med^8^	34.4 (479/1392) (N2)	Orthope-dics and sports medicine	Corresponding authors	Yes	No	78.3 (375/479)	Not reported	Not assessed
Rajaseka-ran^32^	27.2 (247/908) (N3)	Physical medicine and rehabilita-tion	First authors	Yes	No	51.2 (125/244)	48.8 (119/244)	38.2 (71/186)

*N1: Number of emails with questionnaires sent, N2: Number of emails with questionnaires not bounced, N3: Number of questionnaires for which the surveyee was available.

**None of the included surveys gave complete gender/sex breakdowns for all considered categories and only the terms ‘males’ and ‘females’ were used^22^.

### Assessment of methodological quality

The overall confidence in the 67 results in the 15 eligible surveys was rated as either ‘low’ (n = 31) or ‘critically low’ (n = 36). These ratings were based on the 7 ‘critical items of the 14-item quality checklist. The prevalence of the critical and non-critical ratings are in Table 2^33^. The characteristics of the respondents (item 6), and the review items (Item 7) were defined in all surveys. However, whether the characteristics of the respondents were representative for the target population (item 7) was unclear, often caused by partial or poor reporting on socio-demographics and non-reporting on the characteristics of non-responders. The low-quality ratings were predominantly the result of shortcomings in the survey methods (31% (21/67), low response rates (99% (66/67), and inadequate sample sizes (46% (31/67). The overall confidence in the results of each survey question of each eligible survey together with the ratings for the 7 critical items of the quality checklist are presented in the Appendix (Additional item L, pages.

**Table 2:**
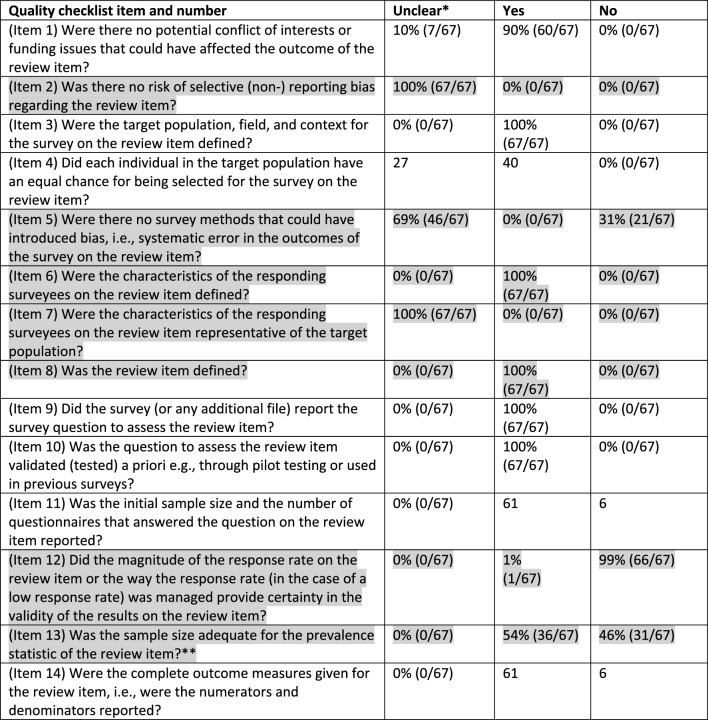
Prevalence of answers to quality checklist questions (the 7 critical items are shaded grey).

*‘Unclear’ was assigned when too few details were reported in the manuscript or additional files to make a judgment of assigning ‘Yes’ or ‘No’.

**The required sample size was calculated with EpiTools epidemiological calculators and was based on the identified prevalence and the total sample size^33^. The estimated prevalence was calculated with a 0.95 confidence level (desired precision of estimate 0.05).

### Results of individual studies and of syntheses

All results to individual survey questions are reported in Tables 3 and 4 and are further explained in the Appendix (Additional item M, pages 38–54). When identical questions were used in more than one study, we conducted meta-analyses. This applied to review items 2a, 2b, and 3a. When a specific question was used by one single survey only, we reported the prevalence with the 95% confidence intervals. This applied to review items 2c, 2d, 3b, and 3d.

**Table 3:** Summary table of the response rates and results for review item 2.

Survey items	Response rate	Prevalence of the practice reported under the review item
*Review Item 2a*. Researchers reporting the practice of automatically listing a senior member(s) of their department (including section chief or department head) as an author on all submitted articles *Question review item 2a:* Is there a senior member of your department (including section chief or department head) who is automatically listed as an author in all submitted manuscripts?	22% (N1*) [95% CI 19–25] (4098 surveyees in 3 surveys) 31% (N2*) [95% CI 25–37] (5808 surveyees in 5 surveys) 35% (N3*) [95% CI 33–36] (2548 surveyees in 2 surveys)	20% [95% CI 16–25] (3619 respondents in 10 surveys)
*Review Item 2b*. Researchers reporting the practice of automatically listing their section or department head as an author on all submitted articles *Question review item 2b:* Is your section or department head automatically listed as an author in all submitted manuscripts?	29% (383/1338) (N1*) [95% CI 26–31] (1338 surveyees in 1survey)^24^ 29% (328/1337) (N2*) [95% CI 22–27] (1337 surveyees in 1survey)^25^ 17% (309/1839) (N3*) [95% CI 15–19] (1839 surveyees in 1survey)^4^	25% [95% CI 22–27] (1020 respondents in 3 surveys)
*Review Item 2c*. Researchers reporting the practice of automatically listing a senior member(s) of their department, including their section chief or department head, as a co-author on a manuscript without fulfilling the ICMJE criteria for authorship *Question review item 2c:* Is there a senior member of your department, including your section chief or department head, who is automatically listed as a coauthor in the review without fulfilling the ICMJE criteria for authorship?	54.5% (666/1221) (N3*) [95% CI 51.7–57.4] (1221 surveyees in 1 survey)^28^	6.8% (45/666) [95% CI 5–8.9] (666 respondents in 1 survey)^28^
*Review Item 2d.* Researchers reporting the practice of automatically listing a senior member(s) of their department, including their section chief or department head, as a co-author(s) on all articles submitted by these researchers *Question review item 2d:* Is there a senior member of your department, including your section chief or department head, who is automatically listed as a coauthor on all of your submitted manuscripts?	27.2% (247/908) (N3*) [95% CI 24.3–30.2] (908 surveyees in 1 survey)^32^	12.6% (31/247) [95% CI 8.7–17.4] (247 respondents in 1 survey)^32^

*N1: Number of emails with questionnaires sent, N2: Number of emails with questionnaires not bounced, N3: Number of questionnaires for which the surveyee was available.

**Table 4:** Summary table of the response rates and results for review item 3.

Survey items	Response rate	Prevalence of the justification ‘never justified’	Prevalence of the justification ‘rarely justified’	Prevalence of the justification ‘sometimes justified’	Prevalence of the justification ‘most of the time justified’	Prevalence of the justification ‘always justified’
*Review item 3a*. Justification of review item 2a, i.e., Researchers reporting the practice of automatically listing a senior member(s) of their department (including section chief or department head) as an author on all submitted articles *Question review item 3a:* If so, do you feel this is justified?	10% (N1*) [95% CI 2–18] (4098 surveyees in 3 surveys) 20% (N2*) [95% CI 8–32] (5808 surveyees in 5 surveys) 24% (N3*) [95% CI 22–26] (2548 surveyees in 2 surveys)	*Review item 3a* 28% [95% CI 22–34] (2180 respondents in 10 surveys)	*Review item 3a* 24% [95% CI 22–27] (2180 respondents in 10 surveys)	*Review item 3a* 25% [95% CI 23–28] (2180 respondents in 10 surveys)	*Review item 3a* 13% [95% CI 9–17] (2180 respondents in 10 surveys)	*Review item 3a* 8% [95% CI 6–9] (2180 respondents in 10 surveys)
*Review item 3b* Justification of review item 2b, i.e., Researchers reporting the practice of automatically listing their section or department head as an author on all submitted articles *Question review item 3b:* If so, do you feel that this is justified in all cases?	7.2% (96/1338) (N1*) [95% CI 5.9–8.7] (1338 surveyees in 1survey)^24^	Not assessed	Not assessed	Not assessed	Not assessed	*Review item 3b* 35.4% (34/96) [95% CI 25.9–45.8] (96 respondents in 1 survey)^24^ This outcome was assessed in 2 other surveys^4,25^, but results were not published
*Review item 3c* Justification of review item 2c, i.e., Researchers reporting the practice of automatically listing a senior member(s) of their department, including their section chief or department head, as a co-author on a manuscript without them fulfilling the ICMJE criteria for authorship	Not assessed	Not assessed	Not assessed	Not assessed	Not assessed	Not assessed
*Review item 3d* Justification of review item 2d, i.e., Researchers reporting the practice of automatically listing a senior member(s) of their department, including their section chief or department head, as a co-author(s) on all articles submitted by these researchers *Question review item 3d:* If yes, do you feel that this is *justified in all cases*?	3.4% (31/908) (N3*) [95% CI 2.3–4.8] (908 surveyees in 1survey)^32^	Not assessed	Not assessed	Not assessed	Not assessed	*Review item 3d* 67.7% (21/31) [95% CI 48.6–83.3] (31 respondents in 1 survey)^32^

*N1: Number of emails with questionnaires sent, N2: Number of emails with questionnaires not bounced, N3: Number of questionnaires for which the surveyee was available.

*Researchers reporting the practice of listing a senior member(s) of a department, who did not qualify for authorship, as co-author(s) on all or most submitted articles by default (Review item 1).*

No surveys addressed this review item.

*Researchers reporting the practice of automatically listing (a) senior member(s) of their department (including section chief or department head) as an author on all submitted articles (Review item 2a) and the justification for this practice (Review item 3a).*

Different questions were used to assess the prevalence of researchers reporting the practice of automatically listing a senior member(s) of their department as an author on all submitted articles (Review item 2) and the justification for this practice (Review item 3). These review items were therefore divided in Review item 2a and 2b and Review item 3a and 3b (Tables 3 and 4).

**Figure 2:**
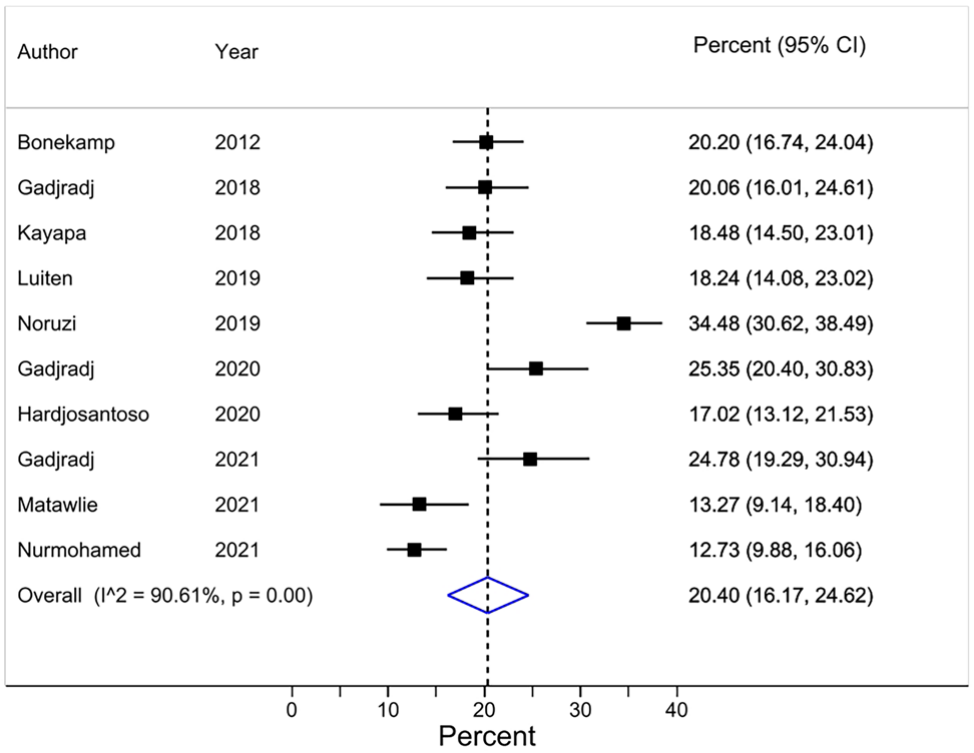
Forest plot for ‘Researchers reporting the practice of automatically listing (a) senior member(s) of their department (including section chief or department head) as an author on all submitted articles’.* Effect Size (ES) 20% [95% CI 16–25]. Heterogeneity *χ*^2^ = 95.84 [d*f* = 9]; *P* < 0.001. Variation in ES attributable to heterogeneity: *I*^2^ = 90.61%. Between-study variance estimate *τ*^2^ = 0.00. Test of ES = 0: *z* = 9.47; *P* < 0.001. *The results of each individual survey were based on the answers to the same question regarding a specific publication by the surveyee.

A pooled average of 20% [95% CI 16–25] of researchers (based on data from 10 surveys, and a total of 3619 respondents) reported that a senior member of their department (including section chief or department head) was automatically listed as an author on all submitted articles (Review item 2a) (Table 3) (Fig. 2). Results were heterogeneous (Chi^2^ = 95.84 (df = 9) *P* < 0.001; I^2^ = 90.61%). 2180 respondents (10 surveys) reported on how they justified this practice. A pooled weighted average of 28% [95% CI 22–34] felt it was ‘never justified’, 24% [95% CI 22–27] ‘rarely justified’, 25% [95% CI 23–28] ‘sometimes justified’, 13% [95% CI 9–17] ‘most of the time justified’, and 8% [95% CI 6–9] felt it was ‘always justified’, respectively. Corresponding forest plots are reported in the Appendix (Additional item M, pages 46–43).

*Researchers reporting the practice of automatically listing their section or department head as an author on all submitted articles (Review Item 2b) and the justification for this practice (Review item 3b).*

Review item 2b (Table 3) did not refer to senior members in general, but specifically to section or department heads and when pooled this led to an average of 25% [95% CI 22–27] of researchers (based on data from 3 surveys, and a total of 1020 respondents). The corresponding forest plot is in the Appendix (Additional item M, pages 52–53). All three surveys were conducted in the field of radiology by the same research group^4,24,25^. Results were homogeneous (Chi^2^ = 0.29 (df = 2) *P* = 0.87; I^2^ = 0.00%). Exploration of this homogeneity was not possible, because the contacted corresponding author reported that individual survey data of all 3 surveys were not available anymore. All 3 surveys assessed the justification of this practice, but only 1 of these surveys^24^ published the results, i.e., 35.4% (34/96) of respondents felt that this practice was justified in all cases (Review item 3b) (Table 4).

*Researchers reporting the practice of automatically listing (a) senior member(s) of their department, including their section chief or department head, as a co-author on a manuscript without fulfilling the ICMJE criteria for authorship (Review Item 2c).*

Review item 2c (Table 3) was addressed in one survey^28^ only and showed that 6.8% [95% CI 5–8.9] of 666 researchers reported that a senior member of their department, including their section chief or department head was automatically listed as a coauthor in a Cochrane review without fulfilling the ICMJE criteria for authorship^3^. The justification of this practice was not assessed (Table 4). The question for this review item differed from the previous questions in that it did not refer to automatically listing of a co-author to all submitted manuscripts, but to a specific manuscript submitted by the surveyee. Further, this question referred to co-authorship without fulfilling the ICMJE criteria for authorship in Cochrane reviews (Appendix, Additional item M, page 54).

*Researchers reporting the practice of automatically listing (a) senior member(s) of their department, including their section chief or department head, as a co-author(s) on all articles submitted by these researchers (Review item 2d) and the justification of this practice (Review item 3d).*

Contrary to the previous review items, review item 2d referred specifically to all manuscripts submitted by the surveyee. This review item was assessed in 1 survey^32^ (Table 3), which showed that 27.2% [95% CI 24.3–30.2] of 908 researchers reported that a senior member of their department including their section chief or department head was automatically listed as a coauthor on all manuscripts submitted by the surveyee. This practice was considered ‘always justified’ by 67.7% of 31 respondents (Review item 3d) (Table 4).

### Response rates

Response rate of each individual survey questions are reported in the Appendix (Additional item M, pages 38–45). Response rates were meta-analyzed when surveys had used the same denominators, e.g., number of emails with questionnaires sent (Tables 3 and 4)^4,24,25,28,32^. It was not always clear which denominators were used to calculate the response rates for the justification of the practice of automatically listing a senior member of a department as an author on all submitted articles (Review item 3). To avoid possible overestimation of these rates, we used the same denominators for review item 3 as those used for review item 2, i.e., researchers reporting the practice of automatically listing a senior member of their department as an author on all submitted articles. Overall, response rates for review item 3 were lower than those for review item 2 (Tables 3 and 4).

### Investigation of heterogeneity and sensitivity analyses

Meta-regression and subgroup analyses identified significant associations (*p* < 0.05) with several explanatory variables, but these associations were all based on small numbers of observations (n = 10). Further, for all tested associations only 1 of the survey characteristics differed from the other 9 surveys, which could further jeopardize the drawing of sound conclusions. We did not conduct sensitivity analyses, because the sources in which the eligible surveys were identified and the survey design and quality of the included surveys were found to be similar. All results of the meta-regression and subgroup analyses are given in the Appendix (Additional item N, pages 55–61).

### Non-reporting biases in syntheses

An overall judgement about risk of bias due to missing results in a synthesis (non-reporting biases) was ‘moderate’ for the results of questions 2a, 2b, and 3a^19^. The methods, results, and rationale for these judgments were reported in the Appendix (Additional item O, pages 62–64).

### Certainty of evidence

Table 5 summarizes the findings of this systematic review and assigns certainty of evidence grades (GRADE)^21^ to each outcome. These grades were either low or very low (Table 5). The rationales for assigning these grades are further explained in the Appendix (Additional item P, pages 65–66).

**Table 5:** Summary of findings.

*Surveyee*: Any author on the author list of a scientific publication, e.g., first, last, corresponding author, that was invited to participate in a survey on at least one of our review items. *Settings*: Any. *Intervention*: Surveys based on questionnaires for self-completion			
Survey items	Prevalence (95% CI)	# of respondents and surveys	Certainty of the evidence (GRADE)*
*Review Item 2a (Question 2a)*. Researchers reporting the practice of automatically listing a senior member(s) of their department (including section chief or department head) as an author on all submitted articles	20% [95% CI 16–25]	3619 respondents in 10 surveys	⊕⊝⊝⊝ *Very low* Due to risk of bias, inconsistency, imprecision, and moderate risk of non-reporting biases
*Review Item 2b (Question 2b)*. Researchers reporting the practice of automatically listing their section or department head as an author on all submitted articles	25% [95% CI 22–27]	1020 respondents in 3 surveys	⊕⊝⊝⊝ *Low* Due to risk of bias, and moderate risk of non-reporting biases
*Review Item 2c (Question 2c)*. Researchers reporting the practice of automatically listing a senior member(s) of their department, including their section chief or department head, as a co-author on a manuscript without fulfilling the ICMJE criteria for authorship	6.8% (45/666) [95% CI 5–8.9]	666 respondents in 1 survey^28^	⊕⊕⊝⊝ *Low* Due to risk of bias, imprecision, and moderate risk of non-reporting biases
*Review Item 2d (Question 2d)*. Researchers reporting the practice of automatically listing a senior member(s) of their department, including their section chief or department head, as a co-author(s) on all articles submitted by these researchers	12.6% (31/247) [95% CI 8.7–17.4]	247 respondents in 1 survey^32^	⊕⊝⊝⊝ *Very low* Due to risk of bias, imprecision, and moderate risk of non-reporting biases
*Review item 3a (Question 3a)*. Justification ‘*Never justified*’ for review item 2a, i.e., researchers reporting the practice of automatically listing a senior member(s) of their department (including section chief or department head) as an author on all submitted articles	28% [95% CI 22–34]	2180 respondents in 10 surveys	⊕⊝⊝⊝ *Very low* Due to risk of bias, inconsistency, imprecision, and moderate risk of non-reporting biases
*Review item 3a (Question 3a)*. Justification ‘*Rarely justified*’ for review item 2a, i.e., researchers reporting the practice of automatically listing a senior member(s) of their department (including section chief or department head) as an author on all submitted articles	24% [95% CI 22–27]	2180 respondents in 10 surveys	⊕⊕⊝⊝ *Low* Due to risk of bias and moderate risk of non-reporting biases
*Review item 3a (Question 3a)*. Justification ‘*Sometimes justified*’ for review item 2a, i.e., researchers reporting the practice of automatically listing a senior member(s) of their department (including section chief or department head) as an author on all submitted articles	25% [95% CI 23–28]	2180 respondents in 10 surveys	⊕⊕⊝⊝ *Low* Due to risk of bias and moderate risk of non-reporting biases
*Review item 3a (Question 3a)*. Justification ‘*Most of the time justified*’ for review item 2a, i.e., researchers reporting the practice of automatically listing a senior member(s) of their department (including section chief or department head) as an author on all submitted articles	13% [95% CI 9–17]	2180 respondents in 10 surveys	⊕⊝⊝⊝ *Very low* Due to risk of bias, inconsistency, imprecision, and moderate risk of non-reporting biases
*Review item 3a (Question 3a)*. Justification ‘*Always justified*’ for review item 2a, i.e., researchers reporting the practice of automatically listing a senior member(s) of their department (including section chief or department head) as an author on all submitted articles	8% [95% CI 6–9]	2180 respondents in 10 surveys	⊕⊝⊝⊝ *Very low* Due to risk of bias, inconsistency, imprecision, and moderate risk of non-reporting biases
*Review item 3b (Question 3b)*. Justification ‘*Justified in all cases*’ for review item 2b, i.e., Researchers reporting the practice of automatically listing their section or department head as an author on all submitted articles	35.4% (34/96) [95% CI 25.9–45.8]	96 respondents in 1 survey^24^	⊕⊝⊝⊝ *Very low* Due to risk of bias, imprecision, and high risk of non-reporting biases
*Review item 3d. (Question 3d)* Justification ‘*Justified in all cases*’ for review item 2d, i.e., Researchers reporting the practice of automatically listing a senior member(s) of their department, including their section chief or department head, as a co-author(s) on all articles submitted by these researchers	67.7% (21/31) [95% CI 48.6–83.3]	31 respondents in 1 survey^32^	⊕⊝⊝⊝ *Very low* Due to risk of bias, imprecision, and non-reporting biases

Prevalence of issues regarding the practice of automatically listing senior members as co-authors on submitted articles.

*The rationales for the certainty grades (GRADE) are further explained in the Appendix (Additional item P, pages 65–66).

## Discussion

### Principal findings

Pooling results from 10 surveys, we found that 20% of researchers across health sciences reported the practice of automatically listing (a) senior member(s) of their department (including section chief or department head) as (a) co-author(s) on all submitted articles. Heterogeneity and inconsistency of results were explored through subgroup analyses and meta-regression, but the small number of included studies (n = 10) prevented us from obtaining robust results (Appendix, Additional item N, pages 55–61). In those same 10 surveys, researchers were also asked on how they judged the practice of automatic authorship and 52% of researchers felt it is ‘never or rarely justified’.

### Comparison with other studies

In a recent systematic review and meta-analysis we found high prevalences for a series of honorary authorship issues in the health sciences^34^. In particular, more than a quarter of researchers perceived at least one of their co-authors as honorary authors on their manuscript, not referring to specific criteria for authorship and more than half when assessed against ICMJE criteria for authorship. This systematic review^34^ as well as another systematic review^35^ addressed different honorary authorship issues than were the focus of our current review. Other reviews on honorary authorship were narrative^36–38^ or integrative^39^.

### Limitations

The main limitation of our study we see is pooling data from surveys of low quality with high likelihood of biases, whose direction is hard to judge. This hinders generalizability and drawing strong conclusions. Nevertheless, surveys based on self-report remain the main approach for quantifying the prevalence of honorary authorship. Also, as our study only covered health sciences, it does not shed light on the prevalence of this practice in other disciplines. Further, it is difficult to interpret the meaning of the statement: “Automatic authorship, defined as automatically listing the senior on all manuscripts is ‘sometimes justified’.” Since, how can something be justified part of the time when the phenomenon itself is happening always, without exceptions?

### Why this study is important and what is next

If automatic listing of senior department members as authors on all submitted articles were as common as the 20% estimate from this meta-analysis suggests, it is worrisome. The finding that in half of these cases such listings were considered ‘unjustified’ is even more problematic. First, automatic authorship disconnects, at least partly, the accountability for the work from those listed as authors. Second, when automatic authors are authorities in their field, the practice may influence the acceptance probabilities of submitted manuscripts. Third, automatic authorship unjustly inflates the publication output of senior researchers, further benefiting their careers in a competitive research environment in which long publication lists are still seen as the crucial element of academic performance. Fourth, automatic authorship may deflate the work done by those who actually merit authorship, although in our courses on research integrity we do encounter, even junior, researchers who actively welcome addition of authoritative names on their papers. In other cases it seems that senior researchers make deals about authorships without asking consent of juniors listed as first authors. We believe that the practice of automatic authorship may be hard to eradicate unless the academic recognition and reward system is overhauled and will pay more attention to for example clarifying authorship contributions at the start of research projects, responsible mentoring, peer review, quality over quantity and the transparency associated with open science work styles^40–43^. Courses on ICMJE and CRediT principles^3,44^ may play a minor role, but are currently mostly directed at junior researchers, possibly creating more frustration than when they had been unaware of the rules around authorship. In our view, a minor role could be played by better protection of whistleblowers and potential funding or legal repercussions for those engaging in automatic authorship. Research institutions play a key role in drawing up rules and monitoring compliance.

## Conclusions

The practice of automatically assigning senior members of departments as co-authors on all submitted manuscripts may be common in the health sciences, with those admitting to this practice finding it unjustified in most cases. These findings, when replicated in high quality surveys, are worrisome and require an effective response, most likely in the realm of the academic reward system.

### Supplementary Information

Supplementary Information.

## Data Availability

All raw and analyzed data of this systematic review are reported in the manuscript and Appendix or were deposited in OSF Storage https://osf.io/4eywp/. We will respond rapidly to requests for additional clarifications on our data. Requests can be made to the corresponding author (RMR) at reyndersmail@gmail.com. *Protocol registration and publication*: The protocol for this systematic review was registered in Open Science Framework. Link: https://osf.io/4eywp/. This protocol was based on our previous published protocol^2^ for a systematic review on honorary authorship issues. Link: https://systematicreviewsjournal.biomedcentral.com/articles/10.1186/s13643-022-01928-1.

## Abbreviations

HA: Honorary authorship
ICMJE: International Committee of Medical Journal Editors
OSF: Open Science Framework
QRP: Questionable research practice

## References

[1] Wager E (2009). Recognition, reward and responsibility: Why the authorship of scientific papers matters. Maturitas.

[2] Meursinge Reynders R, Ter Riet G, Di Girolamo N, Malički M (2022). Honorary authorship in health sciences: A protocol for a systematic review of survey research. Syst. Rev..

[3] International Committee of Medical Journal Editors (ICMJE). *Recommendations for the Conduct, Reporting, Editing, and Publication of Scholarly Work in Medical Journals.* Updated May 2023. [online] Available from: http://www.icmje.org/icmje-recommendations.pdf (accessed 10 August 2023). 25558501

[4] Eisenberg RL, Ngo LH, Heidinger BH, Bankier AA (2018). Honorary authorship in radiologic research articles: Assessment of pattern and longitudinal evolution. Acad. Radiol..

[5] Luiten JD, Verhemel A, Dahi Y, Luiten EJT, Gadjradj PS (2019). Honorary authorships in surgical literature. World J. Surg..

[6] Noruzi A, Takkenberg JJM, Kayapa B, Verhemel A, Gadjradj PS (2019). Honorary authorship in cardiothoracic surgery. J. Thorac. Cardiovasc. Surg..

[7] Gadjradj PS, Jalimsing M, Jalimsing S, Voigt I (2021). Authorship in oral and maxillofacial surgery. J. Maxillofac. Oral Surg..

[8] Nurmohamed FRH, Voigt I, Awadpersad P, Matawlie RHS, Gadjradj PS (2021). Authorship decision-making in the field of orthopedic surgery and sports medicine. J. Clin. Orthop. Trauma.

[9] Page MJ, McKenzie JE, Bossuyt PM, Boutron I, Hoffmann TC, Mulrow CD, Shamseer L, Tetzlaff JM, Akl EA, Brennan SE, Chou R, Glanville J, Grimshaw JM, Hróbjartsson A, Lalu MM, Li T, Loder EW, Mayo-Wilson E, McDonald S, McGuinness LA, Stewart LA, Thomas J, Tricco AC, Welch VA, Whiting P, Moher D (2021). The PRISMA 2020 statement: An updated guideline for reporting systematic reviews. BMJ.

[10] Page MJ, Moher D, Bossuyt PM, Boutron I, Hoffmann TC, Mulrow CD, Shamseer L, Tetzlaff JM, Akl EA, Brennan SE, Chou R, Glanville J, Grimshaw JM, Hróbjartsson A, Lalu MM, Li T, Loder EW, Mayo-Wilson E, McDonald S, McGuinness LA, Stewart LA, Thomas J, Tricco AC, Welch VA, Whiting P, McKenzie JE (2021). PRISMA 2020 explanation and elaboration: updated guidance and exemplars for reporting systematic reviews. BMJ.

[11] Martín-Martín A, Thelwall M, Orduna-Malea E, López-Cózar ED (2021). Google Scholar, Microsoft Academic, Scopus, Dimensions, Web of Science, and OpenCitations’ COCI: A multidisciplinary comparison of coverage via citations. Scientometrics.

[12] Singh VK, Singh P, Karmakar M, Leta J, Mayr P (2021). The journal coverage of Web of Science, Scopus and Dimensions: A comparative analysis. Scientometrics.

[13] Visser M, van Eck NJ, Waltman L (2021). Large-scale comparison of bibliographic data sources: Scopus, Web of Science, Dimensions, Crossref, and Microsoft Academic. Quant. Sci. Stud..

[14] Rayyan QRCI. [online] Available from: https://rayyan.qcri.org/welcome (accessed 10 August 2023).

[15] Lefebvre C, Glanville J, Briscoe S, Littlewood A, Marshall C, Metzendorf M-I, Noel-Storr A, Rader T, Shokraneh F, Thomas J, Wieland LS, Higgins JPT, Thomas J, Chandler J, Cumpston M, Li T, Page MJ, Welch VA (2021). Chapter 4: Searching for and selecting studies. Cochrane Handbook for Systematic Reviews of Interventions Version 6.2 (Updated February 2021).

[16] Shea BJ, Reeves BC, Wells G, Thuku M, Hamel C, Moran J, Moher D, Tugwell P, Welch V, Kristjansson E, Henry DA (2017). AMSTAR 2: A critical appraisal tool for systematic reviews that include randomised or non randomised studies of healthcare interventions, or both. BMJ.

[17] StataCorp (2023). Stata Statistical Software: Release 18.

[18] Deeks JJ, Higgins JPT, Altman DG, Higgins JPT, Thomas J, Chandler J, Cumpston M, Li T, Page MJ, Welch VA (2021). Chapter 10: Analysing data and undertaking meta-analyses. Cochrane Handbook for Systematic Reviews of Interventions Version 6.2 (Updated February 2021).

[19] Page MJ, Higgins JPT, Sterne JAC, Higgins JPT, Thomas J, Chandler J, Cumpston M, Li T, Page MJ, Welch VA (2022). Chapter 13: Assessing risk of bias due to missing results in a synthesis. Cochrane Handbook for Systematic Reviews of Interventions Version 6.3 (Updated February 2022).

[20] Barker TH, Migliavaca CB, Stein C, Colpani V, Falavigna M, Aromataris E, Munn Z (2021). Conducting proportional meta-analysis in different types of systematic reviews: A guide for synthesisers of evidence. BMC Med. Res. Methodol..

[21] Schünemann HJ, Higgins JPT, Vist GE, Glasziou P, Akl EA, Skoetz N, Guyatt GH, Higgins JPT, Thomas J, Chandler J, Cumpston M, Li T, Page MJ, Welch VA (2021). Chapter 14: Completing ‘summary of findings’ tables and grading the certainty of the evidence. Cochrane Handbook for Systematic Reviews of Interventions Version 6.2 (Updated February 2021).

[22] Van Epps H, Astudillo O, del Pozo MY, Marsh J (2022). The sex and gender equity in research (SAGER) guidelines: Implementation and checklist development. Eur. Sci. Ed..

[23] Bonekamp S, Halappa VG, Corona-Villalobos CP, Mensa M, Eng J, Lewin JS, Kamel IR (2012). Prevalence of honorary coauthorship in the American journal of roentgenology. AJR Am. J. Roentgenol..

[24] Eisenberg RL, Ngo L, Boiselle PM, Bankier AA (2011). Honorary authorship in radiologic research articles: Assessment of frequency and associated factors. Radiology.

[25] Eisenberg RL, Ngo LH, Bankier AA (2014). Honorary authorship in radiologic research articles: Do geographic factors influence the frequency?. Radiology.

[26] Gadjradj PS, Fezzazi RE, Meppelder CA, Rietdijk WJ, Matabadal NN, Verhemel A, Harhangi BS (2018). Letter: Honorary authorship in neurosurgical literature: A cross-sectional analysis. Neurosurgery..

[27] Gadjradj PS, Peul WC, Jalimsing M, Arjun Sharma JRJ, Verhemel A, Harhangi BS (2020). Who should merit co-authorship? An analysis of honorary authorships in leading spine dedicated journals. Spine J..

[28] Gülen S, Fonnes S, Andresen K, Rosenberg J (2020). More than one-third of Cochrane reviews had gift authors, whereas ghost authorship was rare. J. Clin. Epidemiol..

[29] Hardjosantoso HC, Dahi Y, Verhemel A, Dahi I, Gadjradj PS (2020). Honorary authorships in the ophthalmological literature. J. Curr. Ophthalmol..

[30] Kayapa B, Jhingoer S, Nijsten T, Gadjradj PS (2018). The prevalence of honorary authorship in the dermatological literature. Br. J. Dermatol..

[31] Matawlie RH, Arjun Sharma JR, de Rooij JD, Sardjoe Mishre G, Huygen FJ, Gadjradj PS (2021). Honorary authorship in high-impact journals in anaesthesia and pain medicine. Br. J. Pain.

[32] Rajasekaran S, Shan RL, Finnoff JT (2014). Honorary authorship: Frequency and associated factors in physical medicine and rehabilitation research articles. Arch. Phys. Med. Rehabil..

[33] Sergeant ESG. Sergeant, ESG, 2018. Epitools Epidemiological Calculators. Ausvet. [Online] Available from: http://epitools.ausvet.com.au (accessed 10 August 2023).

[34] Meursinge Reynders, R.A., Ter Riet, G., Di Girolamo, N., Cavagnetto, D. & Malički M. Honorary authorship is highly prevalent in health sciences: systematic review and meta-analysis of surveys. *Sci Rep.* **14**(1), 4385. 10.1038/s41598-024-54909-w (2024). 10.1038/s41598-024-54909-w PMC10883936 38388672

[35] Marušić A, Bošnjak L, Jerončić A (2011). A systematic review of research on the meaning, ethics and practices of authorship across scholarly disciplines. PLoS One.

[36] Aliukonis V, Poškutė M, Gefenas E (2020). Perish or publish dilemma: Challenges to responsible authorship. Medicina (Kaunas).

[37] Gureyev VN, Lakizo I, Mazov NA (2019). Unethical authorship in scientific publications (A review of the problem). Sci. Tech. Inf. Process..

[38] Tarkang EE, Kweku M, Zotor FB (2017). Publication practices and responsible authorship: A review article. J. Public Health Afr..

[39] Kornhaber RA, McLean LM, Baber RJ (2015). Ongoing ethical issues concerning authorship in biomedical journals: An integrative review. Int. J. Nanomed..

[40] Moher D, Naudet F, Cristea IA, Miedema F, Ioannidis JPA, Goodman SN (2018). Assessing scientists for hiring, promotion, and tenure. PLoS Biol..

[41] Moher D, Bouter L, Kleinert S, Glasziou P, Sham MH, Barbour V, Coriat AM, Foeger N, Dirnagl U (2020). The Hong Kong principles for assessing researchers: Fostering research integrity. PLoS Biol..

[42] Agreement on reforming research assessment. 20 July 2022 [online] Available from: https://coara.eu/agreement/the-agreement-full-text/ (accessed 21 February 2024).

[43] Scholcommlab [online] Available from: https://www.scholcommlab.ca/authorship-guidelines/ (accessed 21 February 2024).

[44] Contributor Role Taxonomy (CRediT). CASRAI CRediT Standard. [Online] Available from: https://credit.niso.org (accessed 10 August 2023).

