# Supplementary material for: Automatically listing senior members of departments as co-authors is highly prevalent in health sciences: meta-analysis of survey research
Source: Sci Rep. 2024 Mar 11;14:5883. doi: 10.1038/s41598-024-55966-x (PMC10928221; doi:10.1038/s41598-024-55966-x)
Supplement: Supplementary file 1 — Supplementary Information. [file 41598_2024_55966_MOESM1_ESM.pdf]

Appendix

This is an appendix to ‘Automatically listing senior members of departments as co-authors is highly prevalent in health sciences: meta-analysis of survey research’ by Reint Meursinge Reynders, Davide Cavagnetto, Gerben ter Riet, Nicola Di Girolamo, and Mario Malički. This appendix further expands on what is presented in the manuscript and reports additional items for both the methods and for the results section. This appendix is based on the appendix for another systematic review on honorary authorship issues by the same authors [1].

Table of contents of additional items for the methods section

Additional item	Description	Page(s)
Additional item A	Glossary of terms and review items	3
Additional item B	Differences between the protocol and the final systematic review	4
Additional item C	Eligibility criteria	5
Additional item D	Search strategies for PubMed, Lens.org, and Dimensions.ai	6
Additional item E	Data collection forms	7-12
Additional item F	Quality assessment: Quality checklist for surveys on Honorary Authorship (HA) items, Guidance for completing the quality checklist for surveys on HA issues, Guidance for rating the overall confidence in the results of a survey	13-17
Additional item G	Occurrence measures and synthesis methods: Defining outcomes, Criteria for a quantitative synthesis, Investigation of heterogeneity	18-20
Additional item H	(Non) reporting bias assessment	21
Additional item I	Guidance for grading the certainty or quality of evidence for a review item	22-23

Table of contents of additional items for the results section

Additional item	Description	Page(s)
Additional item J	Study selection, included surveys and characteristics of included surveys	24-29
Additional item K	Excluded studies with rationale	30-33
Additional item L	Rating of the overall confidence in the results of a survey	34-37
Additional item M	Response rates and results of the survey questions	38-54
Additional item N	Investigation of heterogeneity	55-61
Additional item O	Non-reporting biases	62-64
Additional item P	Certainty of the evidence	65-66
Additional item Q	References for the Appendix	67-70

Methods

Methods for this systematic review were very similar to those for our published protocol 'Honorary authorship in health sciences: a protocol for a systematic review of survey research' [2]. We adapted this protocol to the research questions of this systematic review and registered it in Open Science Framework, https://osf.io/4eywp/. The additional items below explain the methodology used in this systematic review.

Additional item A. Glossary of terms and review items

Appendix Table A1. Glossary of terms and review items

**Term**	**Definition**
Survey [3]	Wikipedia [3] defines a survey as follows: 'In research of human subjects, a survey is a list of questions aimed for extracting specific data from a particular group of people'.
Surveyee	Any author on the author list of a scientific publication, e.g., first, last, corresponding author, that was invited to participate in a survey on at least one of our review items.
Health sciences [4]	Wikipedia [4] defines 'health sciences' as: 'are those sciences which focus on health, or health care, as core parts of their subject matter. Health sciences relate to multiple academic disciplines, including STEM disciplines and emerging patient safety disciplines (such as social care research).
Honorary authorship [2]	Refers to authorship assigned to individuals that should not have been included as authors of a publication, because they made no or insufficient contributions to qualify as authors.
ICMJE-defined criteria for authorship [5]	The ICMJE recommends that authorship is based on the following 4 criteria:<br>1) 'Substantial contributions to the conception or design of the work; or the acquisition, analysis, or interpretation of data for the work; AND<br>2) Drafting the work or revising it critically for important intellectual content; AND<br>3) Final approval of the version to be published; AND<br>4) Agreement to be accountable for all aspects of the work in ensuring that questions related to the accuracy or integrity of any part of the work are appropriately investigated and resolved.'
ICMJE-based honorary authorship	The perception/opinion of the surveyee that one or more of the co-authors did not meet the criteria for authorship of the ICMJE.
Perceived honorary authorship	The perception/opinion of the surveyee that one or more of the co-authors should not have been included as author(s) of a publication, because they made no or insufficient contributions to qualify as authors.
Review item 1	The prevalence of researchers in the health sciences reporting the practice of listing (a) senior member(s) of a department, who did not qualify for authorship, as co-author(s) on all or most submitted articles by default.
Review item 2	The prevalence of researchers in the health sciences reporting the practice of automatically listing (a) senior member(s) of a department as a co-author(s) on all submitted articles.
Review item 3	The prevalence of justifications for this previous practice.

Additional item B. Differences between the protocol and the final systematic review

The following changes to the protocol were implemented:

- For the study selection, data extraction, and quality assessments Nicola Di Girolamo was substituted by Davide Cavagnetto. This replacement was implemented to facilitate these procedures, because both Reint Meursinge Reynders and Davide Cavagnetto collaborate daily at the same institutions. Davide Cavagnetto was calibrated through pilot tests for all three procedures. For the pilot study selection we used a sample of records to screen. For the piloting of the data extraction and quality assessment we used a representative sample of surveys to be reviewed.
- We conducted meta-analysis when one or more criteria precluding such analyses were met. For example, we undertook quantitative syntheses even when the quality of the results of one or more of the included outcomes was rated as 'critically low' or high heterogeneity and inconsistency (I^2>50%) was present [6]. We made this decision, because heterogeneity and low-quality of surveys is common to this research design. We advise readers when using or citing the summary estimates to consider and reflect biases associated with survey research.
- We omitted double arcsine transformation prior to statistical pooling, because recent publications on this topic indicated that double arcsine transformation can lead to misleading results in meta-analyses of proportions [7,8].
- We conducted subgroup analyses to assess whether risk of multiple submissions of questionnaires by surveyees or whether $\geq$ 50% of the surveyees being male were predictors for the results for questions 2a and 3a (Additional item N).
- We did not build generalized linear mixed models to assess which factors contribute by how much to each of the prevalences reviewed, because individual respondent data were not available for any of the 15 included surveys.

Additional item C. Eligibility criteria

We included publications in health sciences which reported on survey results on a series of pre-defined items regarding the practice of listing (a) senior member(s) of a department, as (a) co-author(s) on all or most submitted articles by default. We included publications on this topic in any language, and any setting and time point. Except for the 3 new outcomes, all criteria were identical to those in our previous review on HA in health sciences [2].

Appendix Table A2. Inclusion and exclusion criteria

Item	Inclusion criteria	Exclusion criteria
**Domain**	• Health sciences as defined in Appendix Table A1.	
**Study designs**	• Studies including at least one survey according to its definition in Appendix Table A1.	• Surveys in which it was unclear what questions were used to assess review items 1-3, i.e., surveys which did not report or whose authors were unreachable or did not provide exact questions asked in the survey
**Participants**	• Any author on the author list of a scientific publication, e.g., first, last, corresponding author etc., that was invited to participate in a survey on at least one of our authorship items.	
**Survey instruments**	• Surveys based on questionnaires for self-completion.<br>• Surveys administered by email, internet platforms, by post, or by hand.<br>• We will only consider closed surveys, i.e., surveys open to a specific sample of participants selected by the investigators.<br>• Surveys with or without incentives to complete it.	• Focus groups discussions and one-to one interviews.
**Outcomes**	• One or more of the outcomes on authorship issues listed in our objectives for review items 1-3 (Appendix Table A1).	• Outcomes that were not reported as prevalence statistics or were not given in a format that such statistics could be calculated.
**Time point**	• Any time point for measuring outcomes will be eligible, i.e., we will not set exclusion criteria whether an article on which the surveyee was questioned was published 1, 2, 3 etc. years previously.	
**Setting**	• Any	
**Language**	• Any	
**Publication status**	• Peer-and non-peer-reviewed manuscripts.	
**Publication dates**	• Articles published from bibliography inception onwards.	

Additional item D. Search strategies for PubMed, Lens.org, and Dimensions.ai

Appendix Table A3. Search strategy

PubMed

Date search: January 5 2023

Search strategy: (contributorship[Title/Abstract] OR authorship[Title/Abstract]) AND (survey[Title/Abstract] OR questionnaire[Title/Abstract])

Link:https://pubmed.ncbi.nlm.nih.gov/?term=%28contributorship%5BTitle%2FAbstract%5D+OR+authorship%5BTitle%2FAbstract%5D%29+AND+%28survey%5BTitle%2FAbstract%5D+OR+questionnaire%5BTitle%2FAbstract%5D%29

Results: 356

Lens.org

Date search: January 5 2023

Search strategy: (title:(authorship OR contributorship) OR abstract:(authorship OR contributorship)) AND (title:(survey OR questionnaire) OR abstract:(survey OR questionnaire))

Filters: Field of Study = (Medicine, Medical education, Family medicine, Alternative medicine, Nursing, Nurse education)

Link:
https://www.lens.org/lens/search/scholar/list?q=(title:(authorship%20OR%20contributorship)%20OR%20abstract:(authorship%20OR%20contributorship))%20AND%20(title:(survey%20OR%20questionnaire)%20OR%20abstract:(survey%20OR%20questionnaire))&p=0&n=10&s=_score&d=%2B&f=false&e=false&l=en&authorField=author&dateFilterField=publishedYear&orderBy=%2B_score&presentation=false&stemmed=true&useAuthorId=false&fieldOfStudy.must=Medicine&fieldOfStudy.must=Medical%20education&fieldOfStudy.must=Family%20medicine&fieldOfStudy.must=Alternative%20medicine&fieldOfStudy.must=Nursing&fieldOfStudy.must=Nurse%20education

Results: 498

Dimensions.ai

Date search: January 5 2023

Search strategy: (contributorship or authorship) and (survey or questionnaire)

Filter: Fields of Research: Medical and Health Sciences

Link:
https://app.dimensions.ai/discover/publication?search_mode=content&search_text=(contributorship%20or%20authorship)%20and%20(survey%20or%20questionnaire)&search_type=kws&search_field=full_search

Results: 1098

Additional item E Data collection forms

Data collection was conducted by RMR and DC, independently. Disagreements were resolved through discussions, information provided by the contacted authors of the surveys, or through arbitration by a third reviewer (GTR). All data items to extract were defined in our pilot-tested data extraction forms (Appendix Tables A4-A12). All entries of the data collection forms were collected in Excel spreadsheets.

Appendix Table A4. Data collection forms at the study level in surveys on HA issues*

Entry	Description
Year	Report the year of the publication of the article.
Reference	Report the full reference.
Journal	Report the name of the pertinent journal.
IF (year)	Report the impact factor of the journal. We will report the latest impact factor and the year in which this impact factor was assigned.
Language	Report the language of the article.
Number of authors	Report the number of authors in the article.
Affiliation	Report the type of the first affiliation, i.e., university, industry, care facility, other.
Country first affiliation and name of the first affiliation	Report the country of the first affiliation listed and the name of the institute.
Conflict-of-interest statement reported	Was a conflict-of-interest statement reported? Answer: Yes/No/Unclear. If No, give the rationale.
No potential conflict of interest and funding issues	Were there no potential conflict of interests or funding issues that could have affected the outcomes of the survey? Answer: Yes/No/Unclear. If No, give the rationale.
Registration/publication of the protocol	Was the protocol registered or published a priori? Answer: Yes/No/Unclear. If Yes, we will also extract where and when the protocol was registered.
Institutional Review Board (IRB) approval	Report whether the survey was approved by an IRB. Answer: Yes/No/Unclear.
Institutional Review Board (IRB) exemption	Report whether the survey received an exemption from IRB. Answer: Yes/No/Unclear.
Limitations reported	Did the authors report the limitations of the survey? Answer: Yes/No/Unclear.
To do or contact authors	Report what actions should be undertaken to obtain additional information and whether it is necessary to contact the authors and if so report what information should be obtained.

*We assigned 'Unclear' when too few details were reported in the manuscript or additional files to make a judgment of assigning 'Yes' or 'No'.

Appendix Table A5. Data collection forms at the eligibility level in surveys on HA issues*

Target population	Report the type of target population of the survey, e.g., first or corresponding author or any other author. The target population further refers to characteristics such as assistant, associate, or full professor or head of a department etc.
Target field and context	Report the target field and context of the survey. For the field we refer to e.g., neurology or plastic surgery. For the context we refer to articles, journals, and publication dates on which the target population was surveyed.

*We assigned 'Unclear' when too few details were reported in the manuscript or additional files to make a judgment of assigning 'Yes' or 'No'.

Appendix Table A6. Data collection forms at the sampling level in surveys on HA issues*

Sampling technique	Report what sampling technique was used, e.g., consecutive, i.e., consecutive subjects were sampled, random, non-random, open link on a website, cluster (multistage), sampling weights, strata etc.

*We assigned 'Unclear' when too few details were reported in the manuscript or additional files to make a judgment of assigning 'Yes' or 'No'.

Appendix Table A7. Data collection forms at the survey methods level in surveys on HA issues*

Survey delivery (Subitem 1)	Report the methods of survey delivery, e.g., email, post, telephone etc.
Incentives (Subitem 2)	Report whether incentives were given to surveyees to complete the questionnaires. Answer: Yes/No/Unclear. If Yes, give the type of incentives.
Timeframe (Subitem 3)	Report whether the timeframe between the year of publication of a research publication and the date of the survey on this publication was reported. Answer: Yes/No/Unclear. If Yes, give the time frame.
Multiple (desired) submissions of surveys by the same surveyee (Subitem 4)	Report whether the manuscript reported that the surveyee completed more than 1 survey, e.g., when surveyees who have published multiple articles in the eligible time span were asked to submit a questionnaire for each published article. Answer: Yes/No/Unclear. If Yes, report on these multiple submissions.
Methods to prevent multiple (undesired) submissions of surveys by the same surveyee (Subitem 5)	Report whether methods were implemented for preventing the submitting of more than one questionnaire by the same surveyee when he/she was invited to submit only one. Answer: Yes/No/Unclear. If yes, give the methods.

*We assigned 'Unclear' when too few details were reported in the manuscript or additional files to make a judgment of assigning 'Yes' or 'No'.

Appendix Table A8. Data collection forms at the surveyee level in surveys on HA issues*

Characteristics of the responding surveyees	Report distribution characteristics of responding surveyees such as: Sociodemographic characteristics: distributions of age (measure of central tendency (mean, p50), measure of dispersion [SD, IQR]), Sex/Gender**, career level such as PhD students, seniority etc. country, number of published papers, additional characteristics.
Characteristics of the non-responding surveyees	Were the characteristics of the non-responding surveyees defined? Answer: Yes/No/Unclear.

*We assigned 'Unclear' when too few details were reported in the manuscript or additional files to make a judgment of assigning 'Yes' or 'No'.

**We reported how the terms sex/gender were used and considered (with complete gender/sex breakdown for all considered categories) in the design of each included survey [9].

Appendix Table A9. Data collection forms at the response rate level in surveys on HA issues

Number of emails with questionnaires on HA issues sent (N1)	The total number of emails with questionnaires on HA issues sent.
Number of emails with questionnaires on HA issues not bounced (N2)	The total number of emails with questionnaires on HA issues sent that had surveyees with valid email addresses.
Number of questionnaires for which the surveyee was available (N3)	The total number of emails with questionnaires sent to assess HA issues with surveyees with valid email addresses and for which the surveyee was available. Unavailability can be the result of, e.g., automated responses such as 'out of office', 'study leave', 'on strike', 'vacation leave', 'maternity leave' etc.
Number of partly or completely answered questionnaires (N4)	The total number of questionnaires on HA issues received back in which the questions were answered (either partial or completely).
Number of completely answered questionnaires (N5)	The total number of questionnaires on HA issues received back in which all questions were answered.
Overall response rates in questionnaires on HA issues	N4 or N5/N1, N2, or N3
Sex/Gender prevalence among the respondents*	For example: Number of males/N4 or N5, e.g., the number of males/N4 or N5 or the number of females/N4 or N5.

*We reported how the terms sex/gender were used and considered (with complete gender/sex breakdown for all considered categories) in the design of each included survey [9].

Appendix Table A10 Data collection forms for review item 1*

Review item 1 defined	Was review item 1 defined? Answer: Yes/No/Unclear.
Definition review item 1	Report the definition of review item 1.
Reporting of survey question to assess review item 1	Did the survey (or any additional file) report the survey question to assess review item 1? Answer: Yes/No/Unclear.
Survey question to assess review item 1	Report the survey question to assess review item 1.
Type of answering scale	Yes/No/Don't know etc.
Validation of the survey question to assess review item 1	Was the question to assess review item 1 validated (tested) a priori e.g., through pilot testing or used in previous surveys? Answer: Yes/No/Unclear.
Number of questionnaires that answered the question on review item 1 (N6)	The total number of questionnaires received back in which the question on review item 1 was answered.
Reporting of the response rate on review item 1	Was the initial sample size and the number of questionnaires that answered the question on review item 1 reported? Answer: Yes/No/Unclear. Initial sample size refers to any initial sample size, i.e., N1, N2, N3, N4, or N5
Response rate on review item 1	N6/N1, N2, N3, N4 or N5
Magnitude of the response rate on review item 1	Was the response rate on review item 1 higher than 50%? (Bethlehem 2017) Answer: Yes/No/Unclear.
Number of questionnaires in which the respondents reported review item 1 (N7)	The number of questionnaires in which the respondents reported review item 1, i.e., researchers reporting the practice of listing a senior member(s) of a department, who did not qualify for authorship, as co-author(s) on all or most submitted articles by default
Prevalence of review item 1 (**Primary outcome**)	N7/N6
Sample size on the prevalence of review item 1	Was the sample size adequate for the prevalence statistic of review item 1? Answer: Yes/No.
Approach to statistical analysis for review item 1	Report the approach to statistical analysis (regression, group comparisons) for review item 1.
Complete reporting of outcome measures on review item 1	Were the complete outcome measures given for review item 1, i.e., were the numerators and denominators reported? Answer: Yes/No.
Weighting of the survey results for review item 1	Were the results of the survey for review item 1 weighted, i.e., corrected for selective nonresponse? Answer: Yes/No/Unclear. If Yes, describe the methods.
Additional issues	Report whether additional issues on review item 1 could have affected outcomes. Answer: Yes/No/Unclear. If Yes, explain.

*We will assign 'Unclear' when too few details were reported in the manuscript or additional files to make a judgment of assigning 'Yes' or 'No'.

Appendix Table A11. Data collection forms for review item 2*

Review item 2 defined	Was review item 2 defined? Answer: Yes/No/Unclear.
Definition review item 2	Report the definition of review item 2.
Reporting of survey question to assess review item 2	Did the survey (or any additional file) report the survey question to assess review item 2? Answer: Yes/No/Unclear.
Survey question to assess review item 2	Report the survey question to assess review item 2.
Type of answering scale	Yes/No/Don't know etc.
Validation of the survey question to assess review item 2	Was the question to assess review item 2 validated (tested) a priori e.g., through pilot testing or used in previous surveys? Answer: Yes/No/Unclear.
Number of questionnaires that answered the question on review item 2 (N8)	The total number of questionnaires received back in which the question on review item 2 was answered.
Reporting of the response rate on review item 2	Was the initial sample size and the number of questionnaires that answered the question on review item 2 reported? Answer: Yes/No/Unclear. Initial sample size refers to any initial sample size, i.e., N1, N2, N3, N4, or N5
Response rate on review item 2	N8/N1, N2, N3, N4 or N5
Magnitude of the response rate on review item 2	Was the response rate on review item 2 higher than 50%? (Bethlehem 2017) Answer: Yes/No/Unclear.
Number of questionnaires in which the respondents reported review item 2 (N9)	The number of questionnaires in which the respondents reported review item 2, i.e., researchers reporting the practice of automatically listing a senior member(s) of a department as a co-author(s) on all submitted articles
Prevalence of review item 2	N9/N8
Sample size on the prevalence of review item 2	Was the sample size adequate for the prevalence statistic of review item 2? Answer: Yes/No.
Approach to statistical analysis for review item 2	Report the approach to statistical analysis (regression, group comparisons) for review item 2.
Complete reporting of outcome measures on review item 2	Were the complete outcome measures given for review item 2, i.e., were the numerators and denominators reported? Answer: Yes/No.
Weighting of the survey results for review item 2	Were the results of the survey for review item 2 weighted, i.e., corrected for selective nonresponse? Answer: Yes/No/Unclear. If Yes, describe the methods.
Additional issues	Report whether additional issues on review item 2 could have affected outcomes. Answer: Yes/No/Unclear. If Yes, explain.

*We will assign 'Unclear' when too few details were reported in the manuscript or additional files to make a judgment of assigning 'Yes' or 'No'.

Appendix Table A12. Data collection forms for review item 3*

Compared to our previous protocol (Meursinge Reynders 2022) we only modified the description of the entry ‘Type of answering scale’.

Review item 3 defined	Was review item 3 defined? Answer: Yes/No/Unclear.
Definition review item 3	Report the definition of review item 3
Reporting of survey question to assess review item 3	Did the survey (or any additional file) report the survey question to assess review item 3? Answer: Yes/No/Unclear.
Survey question to assess review item 3	Report the survey question to assess review item 3.
Type of answering scale	Never justified, rarely justified, sometimes justified, most of the time justified, always justified
Validation of the survey question to assess review item 3	Was the question to assess review item 2 validated (tested) a priori e.g., through pilot testing or used in previous surveys? Answer: Yes/No/Unclear.
Number of questionnaires that answered the question on review item 3 (N10)	The total number of questionnaires received back in which the question on review item 3 was answered.
Reporting of the response rate on review item 3	Was the initial sample size and the number of questionnaires that answered the question on review item 3 reported? Answer: Yes/No/Unclear. Initial sample size refers to any initial sample size, i.e., N1, N2, N3, N4, or N5
Response rate on review item 3	N10/N1, N2, N3, N4 or N5
Magnitude of the response rate on review item 3	Was the response rate on review item 3 higher than 50%? [10] Answer: Yes/No/Unclear.
Number of questionnaires in which the respondents reported each type of justification for review item 3 (N11)	The number of questionnaires in which the respondents reported each type of justification for review item, i.e., the type of researchers’ justifications for the practice of automatically listing a senior member of a department as an author on all submitted articles. These justifications refer to: never justified, rarely justified, sometimes justified, most of the time justified, always justified.
Prevalence of each type of justification for review item 3	N11/N10 for each type of justification, i.e., for each type of justification, never justified, rarely justified etc., a separate prevalence statistic will be calculated.
Sample size on the prevalence of review item 3	Was the sample size adequate for the prevalence statistic of review item 3? Answer: Yes/No.
Approach to statistical analysis for review item 3	Report the approach to statistical analysis (regression, group comparisons) for review item 3
Complete reporting of outcome measures on review item 3	Were the complete outcome measures given for review item 3, i.e., were the numerators and denominators reported? Answer: Yes/No.
Weighting of the survey results for review item 3	Were the results of the survey for review item 3 weighted, i.e., corrected for selective nonresponse? Answer: Yes/No/Unclear. If Yes, describe the methods.
Additional issues	Report whether additional issues on review item 3 could have affected outcomes. Answer: Yes/No/Unclear. If Yes, explain.

*We will assign ‘Unclear’ when too few details were reported in the manuscript or additional files to make a judgment of assigning ‘Yes’ or ‘No’.

Additional item F Quality assessment

Quality checklist for surveys on HA items

We used a critical appraisal tool tailored to our review to assess how the non-implementation of specific quality safeguards could have affected each eligible result of each survey. This tool consists of a 14 items pilot-tested checklist [2]. Congruent with the AMSTAR-2 tool [11], 7 of the 14 items were labeled as 'critical' (Items **2, 5, 6, 7, 8, 12, and 13**) (See 'Guidance for rating the overall confidence in the results of the survey'). For each result of each survey we made a quality assessment. We adopted the AMSTAR-2 ratings 'high', 'moderate', 'low', and 'critically low' to rate the overall confidence in each result of each eligible survey. These ratings were reported in tables together with the prevalence of yes, no, and unclear answers to each question of our critical appraisal tool. All assessments and ratings were conducted by RMR and DC, independently. In the case of disagreements, we implemented the same strategies as reported for the study selection and data collection procedures. The 14-item checklist of our quality assessment tool with user's instructions is given in the protocol [2] and under here.

Appendix Table A13. Quality checklist for results of surveys on review item (#)*

#	Item	Question
1	No conflict of interest and funding issues regarding review item (#)	Were there no potential conflict of interests or funding issues that could have affected the outcome of review item (#)? Answer: Yes/No/Unclear. If No, give the rationale.
2	Selective (non) reporting regarding review item (#)	Was there no risk of selective (non-) reporting bias regarding review item (#)? For example: (1) non registering or publication of the review protocol (2) incomplete reporting on the outcomes of review item (#) or changes in definitions of this review item or changes in analyses of this review item that were not congruent with those planned. Answer: Yes/No/Unclear. If No, give the rationale.
3	Target population, field, and context for review item (#) defined	Were the target population, field, and context for the survey on review item (#) defined? Answer: Yes/No/Unclear.
4	Sampling for review item (#)	Did each individual in the target population have an equal chance for being selected for the survey on review item (#)? Answer: Yes/No/Unclear.
5	Survey methods for review item (#)**	Were there no survey methods that could have introduced bias, i.e., systematic error in the outcomes of the survey on review item (#)? Answer: Yes/No/Unclear. If No, give rationale how bias was introduced.
6	Responding surveyees for review item (#) defined	Were the characteristics of the responding surveyees on review item (#) defined? Answer: Yes/No/Unclear.
7	Responding surveyees for review item (#) representative of the target population	Were the characteristics of the responding surveyees on review item (#) representative of the target population? Answer: Yes/No/Unclear.
8	Review item (#) defined	Was review item (#) defined? Answer: Yes/No/Unclear.
9	Reporting of survey question to assess review item (#)	Did the survey (or any additional file) report the survey question to assess review item (#)? Answer: Yes/No/Unclear.
10	Validation of the survey question to assess review item (#)	Was the question to assess review item (#) validated (tested) a priori e.g., through pilot testing or used in previous surveys? Answer: Yes/No/Unclear.
11	Reporting of the response rate on item (#)**	Was the initial sample size and the number of questionnaires that answered the question on review item (#) reported? Answer: Yes/No/Unclear. Initial sample size refers to any initial sample size, i.e., N1, N2, N3, N4, or N5
12	Response rate on review item (#)**	Did the magnitude of the response rate on review item (#) or the way the response rate (in the case of a low response rate) was managed provide certainty in the validity of the results on this review item?<br>Answer: Yes/No/Unclear.
13	Sample size on the prevalence of review item (#)**	Was the sample size adequate for the prevalence statistic of review item (#)? Answer: Yes/No/Unclear.
14	Complete reporting of outcome measures on review item (#)	Were the complete outcome measures given for review item (#), i.e., were the numerators and denominators reported? Answer: Yes/No/Unclear.

*We assigned 'Unclear' when too few details were reported in the manuscript or additional files to make a judgment of assigning 'Yes' or 'No'.
**Guidance for addressing this question is reported under here in the section 'Guidance for completing the checklist for surveys on HA issues'.

Guidance for completing the quality checklist for surveys on HA issues

Answering most items is straightforward. Items that need additional guidance are presented under here.

Item 5. Survey methods

For this item we considered 5 subitems: 1) survey delivery; 2) incentives; 3) timeframe; 4) multiple (desired) submissions by the same surveyee; 5) methods to prevent multiple (undesired) submissions of surveys by the same surveyee. We addressed the signaling questions for each subitems. Based on the answers to these signaling questions we addressed the checklist question. 'No' was assigned when bias could be the result of methodological issues in one or more of these subitems, which could lead to systematic error in the outcomes of the survey. When 'No' was scored we gave the rationale.

Appendix Table A14. Signaling questions for Item 5. Survey methods*

**Subitem**	**Signaling questions**
Survey delivery (Subitem 1)	Were the methods of survey delivery reported, e.g., email, post, telephone etc. Answer: Yes/No/Unclear. If Yes, give the type of survey delivery.
Incentives (Subitem 2)	Report whether incentives were given to surveyees to complete the questionnaires. Answer: Yes/No/Unclear. If Yes, give the type of incentives.
Timeframe (Subitem 3)	Report whether the timeframe between the year of publication of a research publication and the date of the survey on this publication was reported. Answer: Yes/No/Unclear. If Yes, give the time frame.
Multiple (desired) submissions of surveys by the same surveyee (Subitem 4)	Report whether the manuscript reported that the surveyee completed more than 1 survey, e.g., when surveyees who have published multiple articles in the eligible time span were asked to submit a questionnaire for each published article. Answer: Yes/No/Unclear. If Yes, report on these multiple submissions.
Methods to prevent multiple (undesired) submissions of surveys by the same surveyee (Subitem 5)	Report whether methods were implemented for preventing the submitting of more than one questionnaire by the same surveyee when he/she was invited to submit only one. Answer: Yes/No/Unclear. If yes, give the methods.

*We assigned 'Unclear' when too few details were reported in the manuscript or additional files to make a judgment of assigning 'Yes' or 'No'.

Item 7. Characteristics of responding surveyees representative of the target population

Whether the characteristics of the responding surveyees were representative of the target population could only be assessed when the characteristics of both responders and non-responders were reported. 'Unclear' was scored when the characteristics of both categories were not reported or partly reported or when only the characteristics of one of both categories were reported. 'No' was scored when characteristics of both responders and non-responders were reported, but the characteristics differed substantially to have introduced risk of bias. A rationale for each 'no' scores was given.

Item 12. Response rate

The impact of nonresponse on the results of a survey will be little when response rates are high [10], but low response rates may diminish the validity of a survey's results. However, 'Yes' can still be answered to the question in item 12 when response rates are modest, i.e., when authors can show that non-response was not related to the outcome measured and that the characteristics of responders and non-responders are comparable. Whether and how adjustment weighting was implemented was considered when answering the question of item 12. Adjustment weighting refers to correcting for selective nonresponse. For example, assigning higher weights to underrepresented respondents. In this context we also considered the issue of same surveyees submitting more than one questionnaire, e.g., when surveyees who have published multiple articles in the eligible time span for the survey were asked to submit a questionnaire for each published article.

Item 13. Sample size

We calculated the required sample size with EpiTools epidemiological calculators based on the identified prevalence and the total sample size [12]. The estimated prevalence was calculated with a 0.95 confidence level (desired precision of estimate 0.05). The sample size used to calculate the prevalence

of a review item was considered 'inadequate' when this sample size was smaller than the required sample size (as calculated by the EpiTools software) for this prevalence.

Guidance for rating the overall confidence in the results of a survey

Our rating of the overall confidence in the results of a survey reflects how non-implementation of one or more of these 14 safeguard items might possibly have impacted bias of the results of the survey. Because not all results in the same survey are at risk of the same biases, we rated the overall confidence for each result of each survey separately. Seven (Items 3, 5, 6, 7, 8, 12, and 13) of the 14-item checklist were considered 'critical' for this rating. We adopted the rating scheme reported for the AMSTAR 2 critical appraisal tool [11] to assign ratings of the overall confidence in each result of each survey. Appendix Table A15. presents this rating scheme and is an exact copy of the AMSTAR 2 instrument.

Appendix Table A15. Rating the overall confidence in the results of a survey*

**Rating**	**Description**
High	No or one non-critical weakness was scored in the 14-item quality checklist
Moderate	*More than one non-critical weakness** was scored in the 14-item quality checklist
Low	*One critical flaw with or without non-critical weaknesses* was scored in the 14-item quality checklist
Critically low	*More than one critical flaw with or without non-critical weaknesses* was scored in the 14-item quality checklist

*Multiple non-critical weaknesses may diminish confidence in the results of a survey and it may be appropriate to move the overall appraisal down from moderate to low confidence

Tabular presentation for rating the overall confidence in the results of review item (#)

Appendix Table A16. Tabular presentation of the scores of the 14 item quality checklist for review item (#)* **

Reference	***Survey question***	***Item 1***	***Item 2***	***Item 3***	***Item 4***	***Item 5***	***Item 6…. Item 14***	***Overall confidence in the result***

* All critical appraisal scores Yes/No/Unclear will be given for each result

** Overall confidence ratings are: High, Moderate, Low, and Critically low

Additional item G Occurrence measures and synthesis methods

Defining outcomes

The prevalence was the occurrence measure used both in the presentation of single outcomes as well as in the quantitative syntheses. These prevalences were reported with their exact (Wilson) 95% confidence intervals. All outcomes for the three review items were defined in Appendix Table A17 with the pertinent numerators and denominators. We also reported the various response rates measured.

Appendix Table A17. Definition of response rates and outcomes for review items 1-3

Outcome	Definition
Number of emails with questionnaires on HA issues sent (N1)	The total number of emails with questionnaires on HA issues sent.
Number of emails with questionnaires on HA issues not bounced (N2)	The total number of emails with questionnaires on HA issues sent that had surveyees with valid email addresses.
Number of questionnaires for which the surveyee was available (N3)	The total number of emails with questionnaires sent to assess HA issues with surveyees with valid email addresses and for which the surveyee was available. Unavailability can be the result of, e.g., automated responses such as ‘out of office’, ‘study leave’, ‘on strike’, ‘vacation leave’, ‘maternity leave’ etc.
Number of partly or completely answered questionnaires (N4)	The total number of questionnaires on HA issues received back in which the questions were answered (either partial or completely).
Number of completely answered questionnaires (N5)	The total number of questionnaires on HA issues received back in which all questions were answered.
Overall response rates in questionnaires on HA issues	N4 or N5/N1, N2, or N3
Number of questionnaires that answered the question on review item 1* (N6)	The total number of questionnaires received back in which the question on review item 1* was answered.
Response rate on review item 1*	N6/N1, N2, N3, N4 or N5
Number of questionnaires in which the respondents reported review item 1* (N7)	The number of questionnaires in which the respondents reported review item 1*, i.e., researchers reporting the practice of listing a senior member(s) of a department, who did not qualify for authorship, as co-author(s) on all or most submitted articles by default
Prevalence of review item 1*	N7/N6
Number of questionnaires that answered the question on review item 2** (N8)	The total number of questionnaires received back in which the question on review item 2** was answered.
Response rate on review item 2**	N8/N1, N2, N3, N4 or N5
Number of questionnaires in which the respondents reported review item 2** (N9)	The number of questionnaires in which the respondents reported review item 2**, i.e., researchers reporting the practice of automatically listing a senior member(s) of a department as a co-author(s) on all submitted articles
Prevalence of review item 2**	N9/N8
Number of questionnaires that answered the question on review item 3*** (N10)	The total number of questionnaires received back in which the question on review item 3*** was answered.
Response rate on review item 3***	N10/N1, N2, N3, N4, or N5.
Number of questionnaires in which the respondents reported each type of justification for review item 3*** (N11)	The number of questionnaires in which the respondents reported each type of justification for review item 3***, i.e., the type of researchers’ justifications for the practice of automatically listing a senior member of a department as an author on all submitted articles. These justifications refer to: never justified, rarely justified, sometimes justified, most of the time justified, always justified.
Prevalence of each type of justification for review item 3***	N11/N10 for each type of justification, i.e., for each type of justification, never justified, rarely justified etc., a separate prevalence statistic will be calculated.

*Review item 1: Researchers reporting the practice of listing a senior member(s) of a department, who did not qualify for authorship, as co-author(s) on all or most submitted articles by default.
**Review item 2: Researchers reporting the practice of automatically listing a senior member(s) of a department as a co-author(s) on all submitted articles.
***Review item 3: The type of researchers’ justifications for the practice of automatically listing a senior member of a department as an author on all submitted articles. These justifications refer to: never justified, rarely justified, sometimes justified, most of the time justified, always justified.

Synthesis methods

A narrative systematic synthesis was first conducted for all outcomes. When conducting quantitative syntheses, proportions are presented in forest plots with 95% confidence intervals. The meta-analyses were done using metaprop command in Stata 18 [13]. Random-effects models were used, because we expected between-survey variance. Response rates were meta-analyzed when surveys had used the same denominators (e.g., only N1 or only N2, or only N3, See Appendix Table A17).

To address unit-of-analysis issues we checked whether the same surveyees participated more than once in the same survey. To address missing data issues, we contacted either the corresponding authors or those involved in the statistical analysis by email and sent them reminders after one and two weeks. The data were labelled as missing when after 2 weeks no data were received.

Criteria for a quantitative synthesis

As reported in our protocol [2] we planned to refrain from meta-analysis in the following scenarios: (1) less than 2 included surveys (2) very different definitions of outcomes (3) incomplete reporting of proportions (4) biased evidence such as 'Low', and 'Critically low' ratings of the overall confidence in the results of the survey (5) explained and unexplained heterogeneity [6]. In our protocol we also stated that we considered a I^2 larger than 50% as an approximate rule of thumb for not conducting meta-analysis. When applying this rule, we considered that the value of I^2 depends on the direction and magnitude of the outcomes and the strengths of the evidence for the identified heterogeneity [6]. Prior to refraining from meta-analysis, we assessed if solutions were possible for dealing with one or more of these limiting criteria [14,15]. Post hoc changes in the implementation of the criteria for undertaking meta-analyses were reported with rationale.

Investigation of heterogeneity and sensitivity analyses

The presence and extent of heterogeneity was inspected visually by assessing the overlap of the confidence intervals in the forest plot, by conducting the test of homogeneity (Chi^2), and by calculating tau^2 i.e., the estimate of between study variance, and by calculating I^2 to assess the inconsistency in the results of the surveys [6]. We explored the diversity through meta-regression and subgroup analyses of a series of survey-and methodology-related explanatory variables defined in our protocol [2]. We only conducted subgroup analyses and meta-regression when at least ten observations on a potentially explanatory variable were reported (i.e., 10 studies that reported data regarding a specific explanatory variable) [6,16] and at least one of these observations differed from the other observations. For the tests of subgroup differences, we reported the value of chi-square (Q), the degrees of freedom (df), and the p value. A p value of < 0.05 was considered to be statistically significant.

For the meta-regression we reported the regression coefficient, the 95% confidence intervals, and the p value, e.g., (regression coefficient, XX, 95%CI: XX to XX, p=XX). During the review process we also assessed specific issues to explore in sensitivity analyses, e.g., the impact of the quality or the characteristics of the survey design of certain reviews on the results of this systematic review [2].

Additional item H (Non) reporting bias assessment

Cochrane suggests using the term non-reporting biases over reporting biases [17]. Non-reporting biases lead to bias due to missing results [17]. We used various strategies to address these biases such as:

1) Using a broad-spectrum search strategy with a high sensitivity.
2) Assessing the availability of registered or published protocols and if available we assessed whether the planned outcomes were the same as those reported in the completed surveys.
3) Contacting of authors to obtain information on possible multiple publication of the same research data, the availability of unpublished, ongoing surveys, and protocols.
4) We implemented the 6-step framework suggested by Cochrane to assess risk of bias due to missing results in a synthesis [17].

We did not conducted tests for funnel plot asymmetry, because there is no evidence that proportional data adequately adjust for these graphical tests [18].

Additional item I Guidance for grading the certainty or quality of evidence for a review item

We used the GRADE approach [19] for grading the certainty or quality of evidence for each outcome of our planned systematic review on survey research. The GRADE approach assigns four levels of certainty: 'High', 'Moderate', 'Low', and 'Very low certainty' that a point estimate for a specific outcome is correct [19]. The rationale for assigning these ratings for each outcome was given. GRADE ratings for outcomes of interventional studies start with assigning high quality to randomized controlled trials and low quality to observational studies. For surveys we assigned high quality when surveyees had an equal chance of being selected for the survey and low quality when they did not. We started with this initial quality rating and then according to the GRADE approach assessed 5 factors that can lower the quality rating. These 5 factors are presented as domains 1-5 and are explained.

Domain 1. Risk of bias

Domain 2. Inconsistency

Domain 3. Indirectness

Domain 4. Imprecision

Domain 5. Publication bias

Domain 1. Bias in the included surveys.

Bias in the results of the included surveys was based on our 14-item quality checklist reported in Additional item E. For each outcome we assigned one of the following overall confidence ratings: High, Moderate, Low, and Critically low. (See quality checklist for surveys on HA items in Additional item F). The rationale for assigning each type of rating was given for each outcome.

Domain 2. Heterogeneity or inconsistency of results.

We assessed the presence and the extent of heterogeneity. In the forest plots we assessed the overlap of the confidence intervals for the results of the individual surveys. We calculated Tau^2 (Estimate of

between study variance) and Chi^2 tests to measure statistical heterogeneity [6]. We calculated I^2 to quantify inconsistency and used the following rough interpretation of pertinent thresholds for I^2 [6].

0% to 40%**:** might not be important

30% to 60%: may represent moderate heterogeneity

50% to 90%: may represent substantial heterogeneity

75% to 100%: considerable heterogeneity

Domain 3. Indirectness of evidence

Indirectness of evidence was assigned when for example not all, but only a subgroup of corresponding authors (for example only the heads of departments) of a target population were surveyed. Outcomes for such a subgroup were not considered representative for all corresponding authors of that target population.

Domain 4. Imprecision of results

Surveys with few surveyees or with few events are imprecise and have wide confidence intervals [19]. We assessed imprecision for each outcome.

Domain 5. Publication bias

For this domain we assessed whether publication bias is likely. Methods to detect publication bias were reported in the manuscript in the section (Non) reporting bias assessment.

Results

Additional item J. Study selection, included surveys and characteristics of included surveys

The results of the search and selection procedures are presented in a PRISMA flow diagram [20,21]. In total we identified 1,952 records of which 1,584 remained after the removal of duplicates. After screening 69 articles were selected for full-text assessments of which 12 were finally included in the review. Three additional articles were identified during the screening of references. This added up to a total of 15 included surveys. None of these eligible surveys addressed review item 1 as reported in our objectives. Review items 2 and 3 were addressed by 15 questions on researchers reporting the practice of automatically listing a senior member(s) of a department as co-author(s) on submitted articles and 12 questions on the various justifications of this practice. These latter questions could be addressed with the following 5 justifications i.e., ‘never justified’, ‘rarely justified’, ‘sometimes justified’, ‘most of the time justified’, and ‘always justified’. A total of 67 results were obtained. The questionnaires for all included surveys were either identified in the manuscripts or through contacting of authors.

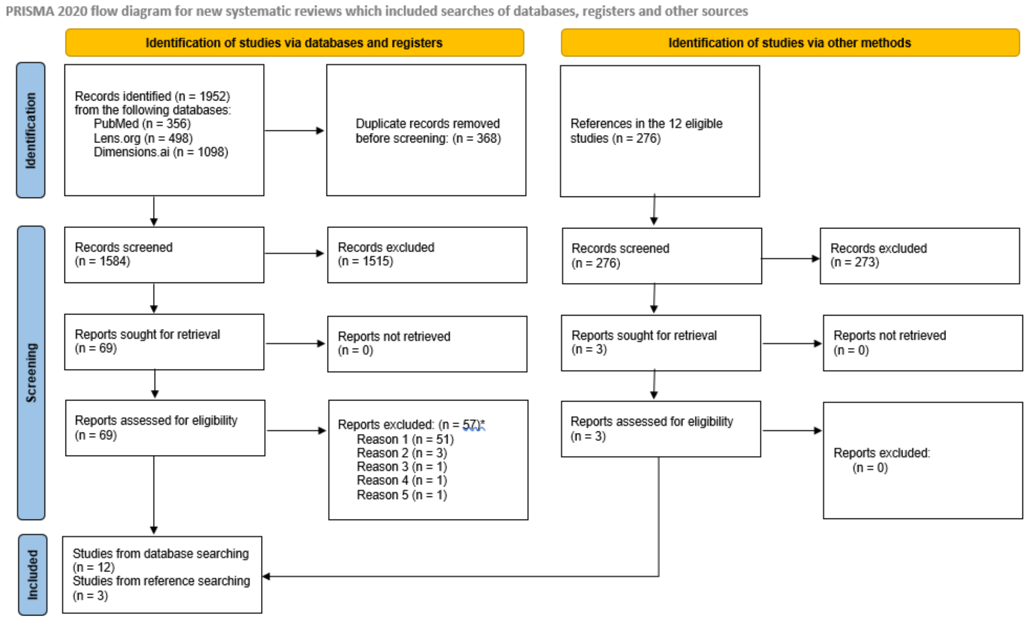

*Reason for exclusion: reason 1: did not assess honorary authorship issues as defined in our protocol; reason 2: could not obtain the survey questionnaire through contacting of the authors; reason 3: non-eligible participants; reason 4: not a survey; reason 5: duplicate

Appendix Figure A1. PRISMA flow diagram

Included surveys

The 15 eligible surveys are reported in Appendix Table A18.

Appendix Table A18. Included surveys

First author/year of publication	Full reference
Bonekamp 2012	Bonekamp S, Halappa VG, Corona-Villalobos CP, Mensa M, Eng J, Lewin JS, Kamel IR. Prevalence of honorary coauthorship in the American Journal of Roentgenology. AJR Am J Roentgenol. 2012 Jun;198(6):1247-55. doi: 10.2214/AJR.11.8253. PMID: 22623536.
Eisenberg 2011	Eisenberg RL, Ngo L, Boiselle PM, Bankier AA. Honorary authorship in radiologic research articles: assessment of frequency and associated factors. Radiology. 2011 May;259(2):479-86. doi: 10.1148/radiol.11101500. Epub 2011 Mar 8. PMID: 21386051.
Eisenberg 2014	Eisenberg RL, Ngo LH, Bankier AA. Honorary authorship in radiologic research articles: do geographic factors influence the frequency? Radiology. 2014 May;271(2):472-8. doi: 10.1148/radiol.13131710. Epub 2013 Nov 27. PMID: 24475845.
Eisenberg 2018	Eisenberg RL, Ngo LH, Heidinger BH, Bankier AA. Honorary Authorship in Radiologic Research Articles: Assessment of Pattern and Longitudinal Evolution. Acad Radiol. 2018;25(11):1451–1456. doi:10.1016/j.acra.2018.02.023
Gadjradj 2018	Gadjradj PS, Fezzazi RE, Meppelder CA, Rietdijk WJ, Matabadal NN, Verhemel A, Harhangi BS. Letter: Honorary Authorship in Neurosurgical Literature: A Cross-sectional Analysis. Neurosurgery. 2018 Jan 1;82(1):E25-E28. doi: 10.1093/neuros/nyx525. PMID: 29053850.
Gadjradj 2020	Gadjradj PS, Peul WC, Jalimsing M, Arjun Sharma JRJ, Verhemel A, Harhangi BS. Who should merit co-authorship? An analysis of honorary authorships in leading spine dedicated journals. Spine J. 2020 Jan;20(1):121-123. doi: 10.1016/j.spinee.2019.08.008. Epub 2019 Aug 21. PMID: 31445166.
Gadjradj 2021	Gadjradj PS, Jalimsing M, Jalimsing S, Voigt I. Authorship in Oral and Maxillofacial Surgery. J Maxillofac Oral Surg. 2021 Jun;20(2):330-335. doi: 10.1007/s12663-021-01538-9. Epub 2021 Mar 16. PMID: 33911405; PMCID: PMC8041930.
Gülen 2020	Gülen S, Fonnes S, Andresen K, Rosenberg J. More than one-third of Cochrane reviews had gift authors, whereas ghost authorship was rare. J Clin Epidemiol. 2020 Dec;128:13-19. doi: 10.1016/j.jclinepi.2020.08.004. Epub 2020 Aug 8. PMID: 32781115.
Hardjosantoso 2020	Hardjosantoso HC, Dahi Y, Verhemel A, Dahi I, Gadjradj PS. Honorary Authorships in the Ophthalmological Literature. J Curr Ophthalmol. 2020 Apr 30;32(2):199-202. doi: 10.4103/JOCO.JOCO_104_20. PMID: 32671306; PMCID: PMC7337016.
Kayapa 2018	Kayapa B, Jhingoer S, Nijsten T, Gadjradj PS. The prevalence of honorary authorship in the dermatological literature. Br J Dermatol. 2018 Jun;178(6):1464-1465. doi: 10.1111/bjd.16678. Epub 2018 May 12. PMID: 29663321.
Luiten 2019	Luiten JD, Verhemel A, Dahi Y, Luiten EJT, Gadjradj PS. Honorary Authorships in Surgical Literature. World J Surg. 2019;43(3):696–703. doi:10.1007/s00268-018-4831-3
Matawlie 2021	Matawlie RH, Arjun Sharma JR, de Rooij JD, Sardjoe Mishre G, Huygen FJ, Gadjradj PS. Honorary authorship in high-impact journals in anaesthesia and pain medicine. Br J Pain. 2021 Aug;15(3):246-248. doi: 10.1177/20494637211023526. Epub 2021 Jun 17. PMID: 34377454; PMCID: PMC8339945.
Noruzi 2019	Noruzi A, Takkenberg JJM, Kayapa B, Verhemel A, Gadjradj PS. Honorary authorship in cardiothoracic surgery [published online ahead of print, 2019 Nov 9]. J Thorac Cardiovasc Surg. 2019;S0022-5223(19)32489-4. doi:10.1016/j.jtcvs.2019.10.104
Nurmohamed 2021	Nurmohamed FRH, Voigt I, Awadpersad P, Matawlie RHS, Gadjradj PS. Authorship decision-making in the field of orthopedic surgery and sports medicine. J Clin Orthop Trauma. 2021 Jul 28;21:101531. doi: 10.1016/j.jcot.2021.101531. PMID: 34405087; PMCID: PMC8348525.
Rajasekaran 2014	Rajasekaran S, Shan RL, Finnoff JT. Honorary authorship: frequency and associated factors in physical medicine and rehabilitation research articles. Arch Phys Med Rehabil. 2014;95(3):418–428. doi:10.1016/j.apmr.2013.09.024

Characteristics of included surveys

In Appendix Tables A19-21 below we reported the characteristics of the 15 included surveys. Denominators of the different prevalence data of individual surveys were not always the same, because some surveyees did not respond to all questions of a survey. Nine surveys were conducted by researchers in the Netherlands and in 9 of the eligible surveys, corresponding authors were the surveyees. The characteristics of the responding surveyees were given in each eligible survey and those of the non-responders were not reported in any survey. None of the included surveys gave complete gender/sex breakdowns for all considered categories and only the terms 'males' and 'females' were used [9]. Males were the predominant responding surveyees (>51%) in 10 of the 11 surveys that reported the prevalence of responding males/females. The percentage of associate professors or higher among the surveyees was 30% or more in 9 of the 10 surveys that reported this outcome.

The majority of surveys (13/15) reported a conflict-of-interest statement and limitations of the conducted survey (13/15). Participants in all eligible surveys were sampled consecutively and were contacted via email. Seven of the included surveys implemented methods to avoid the risk of multiple submissions by the same surveyee, but in 4 surveys such methods were not implemented and in another 4 nothing was reported on such methods. None of the surveys gave incentives to participate in a survey. The time frame between the publication of the pertinent publication and the survey was 1 year or less in 7 of 14 surveys. Extraction of the prevalences of the countries or continents of origin of surveyees could not be extracted reliably, because of partial, imprecise, or non-reporting of this information in the eligible surveys.

Appendix Table A19. Characteristics of included surveys

Study/year of publication	Number of authors	Country first affiliation	Target field	Target population
Bonekamp 2012	7	USA	Radiology	Corresponding authors
Eisenberg 2011	4	USA	Radiology	First authors
Eisenberg 2014	3	USA	Radiology	First authors
Eisenberg 2018	4	USA	Radiology	First authors
Gadjradj 2018	7	The Netherlands	Neurosurgery	Corresponding authors
Gadjradj 2020	7	The Netherlands	Spine	Corresponding authors
Gadjradj 2021	4	The Netherlands	Oral and maxillofacial surgery	Corresponding authors
Gülen 2020	4	Denmark	Cochrane reviews	First authors
Hardjosantoso 2020	5	The Netherlands	Ophthamology	Corresponding authors
Kayapa 2018	4	The Netherlands	Dermatology	Corresponding authors
Luiten 2019	5	The Netherlands	General surgery	Corresponding authors
Matawlie 2021	6	The Netherlands	Pain medicine	Mix of corresponding, first, and senior authors
Noruzi 2019	5	The Netherlands	Cardiothoracic surgery	Corresponding authors
Nurmohamed 2021	5	The Netherlands	Orthopedics and sports medicine	Corresponding authors
Rajasekaran 2014	3	Canada	Physical medicine and rehabilitation	First authors

Appendix Table A20. Characteristics of included surveys

Study/year of publication	Characteristics of the responding surveyees reported	Characteristics of the non-responding surveyees reported	% Males among responding surveyees*	% Females among responding surveyees*	% Associate professor and higher among responding surveyees	Continent of origin of the surveyee**
Bonekamp 2012	Yes	No	70.0% (343/490)	30.0% (147/490)	48.8% (239/490)	Not assessed
Eisenberg 2011	Yes	No	76.3% (299/392)	23.7% (93/392)	38.0% (149/392)	Not assessed
Eisenberg 2014	Yes	No	Not reported	Not reported	21.3% (23/108)	Unclear
Eisenberg 2018	Yes	No	Not reported	Not reported	32.0% (73/228)	Unclear
Gadjradj 2018	Yes	No	88.4% (313/354)	11.6% (41/354)	51.7% (193/373)	Unclear
Gadjradj 2020	Yes	No	80.4 (229/285)	19.6% (56/285)	Not assessed	Unclear
Gadjradj 2021	Yes	No	74.9% (170/227)	25.1% (57/227)	Not assessed	Unclear
Gülen 2020	Yes	No	44.6% (297/666)	54.8% (365/666)	32.1% (225/700)	Not assessed
Hardjosantoso 2020	Yes	No	Not reported	Not reported	Not assessed	Unclear
Kayapa 2018	Yes	No	61.4% (210/342)	38.6% (132/342)	56.2% (187/333)	Unclear
Luiten 2019	Yes	No	77.4% (236/305)	22.6% (69/305)	60.1% (179/298)	Unclear
Matawlie 2021	Yes	No	Not reported	Not reported	Not assessed	Unclear
Noruzi 2019	Yes	No	86.3% (505/585)	13.7% (80/585)	50.1% (293/585)	Unclear
Nurmohamed 2021	Yes	No	78.3% (375/479)	Not reported	Not assessed	Unclear
Rajasekaran 2014	Yes	No	51.2% (125/244)	48.8% (119/244)	38.2% (71/186)	Unclear

*None of the included surveys gave complete gender/sex breakdowns for all considered categories and only the terms 'males' and 'females' were used [9].
**The continent of origin of the surveyee was either not assessed or Unclear..

Appendix Table A21. Characteristics of included surveys

Study/year of publication	Conflict of interest statement reported	Registration or publication of a protocol	Sampling	Survey delivery	Risk of multiple submissions of surveys by the same surveyee*	Time frame between publishing the manuscript and being surveyed	Incentives given	Limitations reported
Bonekamp 2012	Not reported	Not reported	Consecutively	Email	No	2-8 years	Not reported	Yes
Eisenberg 2011	Yes	Not reported	Consecutively	Email	No	3 years	Not reported	Yes
Eisenberg 2014	Yes	Not reported	Consecutively	Email	No	2-3 years	Not reported	Yes
Eisenberg 2018	Not reported	Not reported	Consecutively	Email	No	2-3 years	Not reported	Yes
Gadjradj 2018	Yes	Not reported	Consecutively	Email	Unclear	Not reported	Not reported	Not reported
Gadjradj 2020	Yes	Not reported	Consecutively	Email	Unclear	2 years	Not reported	Not reported
Gadjradj 2021	Yes	Not reported	Consecutively	Email	Unclear	1 year	Not reported	Yes
Gülen 2020	Yes	Not reported	Consecutively	Email	No	≥30 months	Not reported	Yes
Hardjosantoso 2020	Yes	Not reported	Consecutively	Email	Yes	1 year	Not reported	Yes
Kayapa 2018	Yes	Not reported	Consecutively	Email	Yes	1 year	Not reported	Yes
Luiten 2019	Yes	Not reported	Consecutively	Email	No	1 year	Not reported	Yes
Matawlie 2021	Yes	Not reported	Consecutively	Email	Unclear	1 year	Not reported	Yes
Noruzi 2019	Yes	Not reported	Consecutively	Email	No	1 year	Not reported	Yes
Nurmohamed 2021	Yes	Not reported	Consecutively	Email	Yes	1 year	Not reported	Yes
Rajasekaran 2014	Yes	Not reported	Consecutively	Email	Yes	≤ 3 years and 6 months	Not reported	Yes

*Submitting multiple surveys is possible when an author had published more than once in any of the eligible journals and completed a survey on each of their published manuscripts. No, is assigned when methods to prevent this were implemented. Unclear, is assigned when methods to prevent this were not reported.

Additional item K. Excluded studies with rationale

Appendix Table A22. Excluded studies with rationale

Reference	Year	Rationale for exclusion after full text assessment
Al-Herz W, Haider H, Al-Bahhar M, Sadeq A. Honorary authorship in biomedical journals: how common is it and why does it exist? J Med Ethics. 2014 May;40(5):346-8. doi: 10.1136/medethics-2012-101311. Epub 2013 Aug 17. PMID: 23955369.	2014	Did not assess HA issues as defined in our protocol
Ilakovac V, Fister K, Marusic M, Marusic A. Reliability of disclosure forms of authors' contributions. CMAJ. 2007 Jan 2;176(1):41-6. doi: 10.1503/cmaj.060687. PMID: 17200389; PMCID: PMC1764586.	2007	Did not assess HA issues as defined in our protocol
McClellan JM, Mansukhani N, Moe D, Derickson M, Chiu S, Kibbe MR, Martin MJ. Courtesy Authorship in Academic Surgery Publications. JAMA Surg. 2019 Dec 1;154(12):1110-1116. doi: 10.1001/jamasurg.2019.3140. PMID: 31532464; PMCID: PMC6752091.	2019	Did not assess HA issues as defined in our protocol
Shah A, Rajasekaran S, Bhat A, Solomon JM. Frequency and Factors Associated With Honorary Authorship in Indian Biomedical Journals: Analysis of Papers Published From 2012 to 2013. J Empir Res Hum Res Ethics. 2018 Apr;13(2):187-195. doi: 10.1177/1556264617751475. Epub 2018 Jan 18. PMID: 29345178.	2018	Did not assess HA issues as defined in our protocol
Slone RM. Coauthors' contributions to major papers published in the AJR: frequency of undeserved coauthorship. AJR Am J Roentgenol. 1996 Sep;167(3):571-9. doi: 10.2214/ajr.167.3.8751654. PMID: 8751654.	1996	Did not assess HA issues as defined in our protocol
Rajasekaran S, Lo A, Aly AR, Ashworth N. Honorary authorship in postgraduate medical training. Postgrad Med J. 2015 Sep;91(1079):501-7. doi: 10.1136/postgradmedj-2015-133493. Epub 2015 Aug 25. PMID: 26306503.	2015	Did not assess HA issues as defined in our protocol
Derickson M, McClellan JM, Mansukhani NA, Kibbe MR, Martin MJ. Variations in Courtesy Authorship Perceptions and Practices Among Modern Surgical Journals: The Generation Gap. J Surg Res. 2020 Oct;254:242-246. doi: 10.1016/j.jss.2020.04.034. Epub 2020 May 29. PMID: 32480067.	2020	Did not assess HA issues as defined in our protocol
Ashkenazi I, Olsha O. Honorific authorship and approval of the ICMJE criteria: A survey with a convenience sample. Learned publishing. 2021;34(4):647-654.	2021	Did not assess HA issues as defined in our protocol
Justin GA, Miller SC, Tsou B, Li X, Purt B, Fliotsos MJ, Zhao J, Gardner SE, Legault GL, Yonekawa Y, Rapuano CJ, Woreta FA, Pelton RW. Ghost and Honorary Authorship in Ophthalmology: A Cross-Sectional Survey. Am J Ophthalmol. 2022 Aug;240:67-78. doi: 10.1016/j.ajo.2022.02.012. Epub 2022 Feb 25. PMID: 35227695.	2022	Did not assess HA issues as defined in our protocol
Kwee TC, Almaghrabi M, Kwee RM. Scientific fraud, publication bias, and honorary authorship in nuclear medicine. J Nucl Med. 2022 Sep 8:jnumed.122.264679. doi: 10.2967/jnumed.122.264679. Epub ahead of print. PMID: 36215567.	2022	Did not assess HA issues as defined in our protocol
Wislar JS, Flanagin A, Fontanarosa PB, Deangelis CD. Honorary and ghost authorship in high impact biomedical journals: a cross sectional survey. BMJ. 2011 Oct 25;343:d6128. doi: 10.1136/bmj.d6128. PMID: 22028479; PMCID: PMC3202014.	2011	Did not assess HA issues as defined in our protocol
Aldughmi M, Qutaishat D, Karasneh R. Knowledge and Perceptions of Honorary Authorship among Health Care Researchers: Online Cross-sectional Survey Data from the Middle East. Sci Eng Ethics. 2021 Jun 7;27(3):39. doi: 10.1007/s11948-021-00317-6. PMID: 34100137.	2021	Non eligible participants
Kennedy MS, Barnsteiner J, Daly J. Honorary and ghost authorship in nursing publications. J Nurs Scholarsh. 2014 Nov;46(6):416-22. doi: 10.1111/jnu.12093. Epub 2014 Jun 13. PMID: 24930670.	2014	Did not assess HA issues as defined in our protocol
Condron ME, Kibbe MR, Azarow KS, Martin MJ. Courtesy Authorship Practices Among First and Senior Authors: Evaluation of Motivations, Gender Bias, and Inequities. Ann Surg. 2021 Sep 1;274(3):434-440. doi: 10.1097/SLA.0000000000004999. PMID: 34132701.	2021	Did not assess HA issues as defined in our protocol
Vinther S, Rosenberg J. Appearance of ghost and gift authors in Ugeskrift for Læger and Danish Medical Journal. Dan Med J. 2012 May;59(5):A4455. PMID: 22549492.	2012	We were unable to obtain the survey questions. Excluded, because the authors did not respond to 2 emails to get the questionnaires. Both emails were

		sent in May 2022. One on May 10 and repeated on May 21.
Hadji M, Asghari F, Yunesian M, Kabiri P, Fotouhi A. Assessing the Prevalence of Publication Misconduct among Iranian Authors Using a Double List Experiment. Iran J Public Health. 2016 Jul;45(7):897-904. PMID: 27516996; PMCID: PMC4980344.	2016	Did not assess HA issues as defined in our protocol
Mowatt G, Shirran L, Grimshaw JM, et al. Prevalence of honorary and ghost authorship in Cochrane reviews. JAMA. 2002;287:2769-2771.	2002	Did not assess HA issues as defined in our protocol
Dotson B, Slaughter RL. Prevalence of articles with honorary and ghost authors in three pharmacy journals. Am J Health Syst Pharm. 2011 Sep 15;68(18):1730-4. doi: 10.2146/ajhp100583. PMID: 21880889.	2011	Did not assess HA issues as defined in our protocol
Ivaniš A. Značajke autorstva znanstvenih članaka u akademskoj medicini. 2010	2010	Did not assess HA issues as defined in our protocol
Ivanis A, Hren D, Sambunjak D, Marusić M, Marusić A. Quantification of authors' contributions and eligibility for authorship: randomized study in a general medical journal. J Gen Intern Med. 2008;23(9):1303-1310. doi:10.1007/s11606-008-0599-8	2008	Did not assess HA issues as defined in our protocol
Shapiro DW, Wenger NS, Shapiro MF. The contributions of authors to multiauthored biomedical research papers. JAMA. 1994 Feb 9;271(6):438-42. PMID: 8295318.	1994	Did not assess HA issues as defined in our protocol
Masic I. The Malversations of Authorship - Current Status in Academic Community and How to Prevent It. Acta Inform Med. 2018;26(1):4-9. doi: 10.5455/aim.2018.26.4-9. PMID: 29719305; PMCID: PMC5869232.	2018	Did not assess HA issues as defined in our protocol
Flanagin A, Carey LA, Fontanarosa PB, Phillips SG, Pace BP, Lundberg GD, Rennie D. Prevalence of articles with honorary authors and ghost authors in peer-reviewed medical journals. JAMA. 1998 Jul 15;280(3):222-4. doi: 10.1001/jama.280.3.222. PMID: 9676661.	1998	Did not assess HA issues as defined in our protocol
Ivaniš A, Hren D, Marušić M, Marušić A. Less work, less respect: authors' perceived importance of research contributions and their declared contributions to research articles. PLoS One. 2011;6(6):e20206. doi: 10.1371/journal.pone.0020206. Epub 2011 Jun 21. PMID: 21713036; PMCID: PMC3119662.	2011	Did not assess HA issues as defined in our protocol
Okonta P, Rossouw T. Prevalence of scientific misconduct among a group of researchers in Nigeria. Dev World Bioeth. 2013 Dec;13(3):149-57. doi: 10.1111/j.1471-8847.2012.00339.x. Epub 2012 Sep 20. PMID: 22994914; PMCID: PMC3530634.	2013	Did not assess HA issues as defined in our protocol
Artino AR Jr, Driessen EW, Maggio LA. Ethical Shades of Gray: International Frequency of Scientific Misconduct and Questionable Research Practices in Health Professions Education. Acad Med. 2019 Jan;94(1):76-84. doi: 10.1097/ACM.0000000000002412. PMID: 30113363.	2019	Did not assess HA issues as defined in our protocol
Hoen WP, Walvoort HC, Overbeke AJ. What are the factors determining authorship and the order of the authors' names? A study among authors of the Nederlands Tijdschrift voor Geneeskunde (Dutch Journal of Medicine). JAMA. 1998 Jul 15;280(3):217-8. doi: 10.1001/jama.280.3.217. PMID: 9676659.	1998	We were unable to obtain the survey questions through contacting of the authors. No reference or link to questionnaire was given.
Marusic A, Bates T, Anic A, Marusic M (2006) How the structure of contribution disclosure statements affects validity of authorship: a randomized study in a general medical journal. Curr Med Res Opin 22: 1035–44.	2006	Did not assess HA issues as defined in our protocol
Swank JM, Houseknecht A, Puig A, Authorship Decision-Making: A National Survey of Counselor Educators. JCPS 2019;12(2)	2019	Did not assess HA issues as defined in our protocol
Rees CA, Keating EM, Dearden KA, Haq H, Robison JA, Kazembe PN, Bourgeois FT, Niescierenko M. Importance of authorship and inappropriate authorship assignment in paediatric research in low- and middle-income countries. Trop Med Int Health. 2019 Oct;24(10):1229-1242. doi: 10.1111/tmi.13295. Epub 2019 Aug 21. PMID: 31374140.	2019	Did not assess HA issues as defined in our protocol
Chambers LM, Watson CH, Yao M, Levinson K, Alvarez RD, Eskander RN, Buechel M, Michener CM, Jernigan A. Survey of trends in authorship assignment in gynecologic oncology: Keeping score and playing fair. Gynecol Oncol Rep. 2021 Mar 23;36:100755. doi: 10.1016/j.gore.2021.100755. PMID: 33855146; PMCID: PMC8027688.	2021	Did not assess HA issues as defined in our protocol

Kwee RM, Almaghrabi MT, Kwee TC. Scientific integrity and fraud in radiology research. Eur J Radiol. 2022 Oct 8;156:110553. doi: 10.1016/j.ejrad.2022.110553. Epub ahead of print. PMID: 36228454.	2022	Did not assess HA issues as defined in our protocol
Smith E, Williams-Jones B, Master Z, Larivière V, Sugimoto CR, Paul-Hus A, Shi M, Resnik DB. Misconduct and Misbehavior Related to Authorship Disagreements in Collaborative Science. Sci Eng Ethics. 2020 Aug;26(4):1967-1993. doi: 10.1007/s11948-019-00112-4. Epub 2019 Jun 3. PMID: 31161378; PMCID: PMC6888995.	2020	Did not assess HA issues as defined in our protocol
Dhaliwal U, Singh N, Bhatia A. Awareness of authorship criteria and conflict: survey in a medical institution in India. MedGenMed. 2006 Dec 12;8(4):52. PMID: 17415332; PMCID: PMC1868341.	2006	Did not assess HA issues as defined in our protocol
Badreldin H, Aloqayli S, Alqarni R, Alyahya H, Alshehri A, Alzahrani M, Al Tawalbeh A, Ismail WW. Knowledge and Awareness of Authorship Practices Among Health Science Students: A Cross-Sectional Study. Adv Med Educ Pract. 2021 Apr 20;12:383-392. doi: 10.2147/AMEP.S298645. PMID: 33907488; PMCID: PMC8069121.	2021	Did not assess HA issues as defined in our protocol
Nylenna M, Fagerbakk F, Kierulf P. Authorship: attitudes and practice among Norwegian researchers. BMC Med Ethics. 2014 Jul 2;15:53. doi: 10.1186/1472-6939-15-53. PMID: 24989359; PMCID: PMC4118778.	2014	Did not assess HA issues as defined in our protocol
Patience GS, Galli F, Patience PA, Boffito DC. Intellectual contributions meriting authorship: Survey results from the top cited authors across all science categories. PLoS One. 2019 Jan 16;14(1):e0198117. doi: 10.1371/journal.pone.0198117. PMID: 30650079; PMCID: PMC6334927.	2019	Did not assess HA issues as defined in our protocol
Pulsipher KJ, Presley CL, Szeto MD, Barber C, Rietcheck HR, Meckley AL, Militello M, Runion TM, Rundle CW, Dellavalle RP. A survey of osteopathic physician and student authorship in the dermatology literature. Dermatol Online J. 2021 Mar 15;27(3):13030/qt53w5s2vs. PMID: 33865292.	2021	Did not assess HA issues as defined in our protocol
Helgesson G, Juth N, Schneider J, Lövtrup M, Lynøe N. Misuse of Coauthorship in Medical Theses in Sweden. J Empir Res Hum Res Ethics. 2018 Oct;13(4):402-411. doi: 10.1177/1556264618784206. Epub 2018 Jul 9. PMID: 29985088.	2019	Did not assess HA issues as defined in our protocol
Ghajarzadeh M. Guest authors in an Iranian journal. Dev World Bioeth. 2014 Apr;14(1):15-9. doi: 10.1111/dewb.12002. Epub 2012 Oct 1. PMID: 23025813.	2014	Did not assess HA issues as defined in our protocol
Ljubenković AM, Borovečki A, Ćurković M, Hofmann B, Holm S. Survey on the Research Misconduct and Questionable Research Practices of Medical Students, PhD Students, and Supervisors at the Zagreb School of Medicine in Croatia. J Empir Res Hum Res Ethics. 2021 Oct;16(4):435-449. doi: 10.1177/15562646211033727. Epub 2021 Jul 26. PMID: 34310249.	2021	Did not assess HA issues as defined in our protocol
Landa-Blanco M, Santos-Midence C, Landa Blanco A, Academic integrity: attitudes and practices of students of a public university in Honduras. Academia. 2020; Vol 20-21:202-2017.	2020	Did not assess HA issues as defined in our protocol
Osareh F, Serati Shirazi M, Khademi R. A Survey on Co-authorship Network of Iranian Researchers in the field of Pharmacy and Pharmacology in Web of Science during 2000-2012. jha 2014; 17 (56) :33-45	2014	Did not assess HA issues as defined in our protocol
Tarnow E, De Young BR, Cohen MB. Coauthorship in pathology, a comparison with physics and a survery-generated and member-preferred authorship guideline. MedGenMed. 2004 Jul 22;6(3):1-2. PMID: 15520623; PMCID: PMC1435638.	2004	Did not assess HA issues as defined in our protocol
Mitcheson H, Collings S, Siebers RW. Authorship issues at a New Zealand academic institution. Int J Occup Environ Med. 2011 Jul;2(3):166-71. PMID: 23022833.	2011	Did not assess HA issues as defined in our protocol
Kratz JE, Strasser C. Researcher perspectives on publication and peer review of data. PLoS One. 2015 Feb 23;10(2):e0117619. doi: 10.1371/journal.pone.0117619. Erratum in: PLoS One. 2015;10(4):e0123377. PMID: 25706992; PMCID: PMC4338305.	2015	Did not assess HA issues as defined in our protocol
Dhingra D, Mishra D. Publication misconduct among medical professionals in India. Indian J Med Ethics. 2014 Apr 1;11(2):104-7. doi: 10.20529/IJME.2014.026. PMID: 24727622.	2014	Did not assess HA issues as defined in our protocol

Pupovac V, Prijić-Samaržija S, Petrovečki M. Research Misconduct in the Croatian Scientific Community: A Survey Assessing the Forms and Characteristics of Research Misconduct. Sci Eng Ethics. 2017 Feb;23(1):165-181. doi: 10.1007/s11948-016-9767-0. Epub 2016 Mar 3. PMID: 26940319.	2017	Did not assess HA issues as defined in our protocol
Jacard M, Herskovic V, Hernandez I, Reyes H. An analysis of authorship in articles published in Revista Medica de Chile [Spanish]. Rev Med Chil 2002;130:1391-8.	2002	Did not assess HA issues as defined in our protocol
Reinisch JF, Li WY, Yu DC, Walker JW. Authorship conflicts: a study of awareness of authorship criteria among academic plastic surgeons. Plast Reconstr Surg. 2013 Aug;132(2):303e-310e. doi: 10.1097/PRS.0b013e3182958b5a. PMID: 23897358.	2013	Did not assess HA issues as defined in our protocol
Goodman NW. Survey of fulfillment of criteria for authorship in published medical research. BMJ. 1994 Dec 3;309(6967):1482. doi: 10.1136/bmj.309.6967.1482. PMID: 7804054; PMCID: PMC2541657.	1994	Did not assess HA issues as defined in our protocol
Bekkelund SI, Hegstad AC, Førde OH. Uredelighet i medisinsk og helsefaglig forskning i Norge [Scientific misconduct and medical research in Norway]. Tidsskr Nor Laegeforen. 1995 Oct 20;115(25):3148-51. Norwegian. PMID: 8539699.	1995	Did not assess HA issues as defined in our protocol
Rennie D. Freedom and responsibility in medical publication: setting the balance right. JAMA. 1998 Jul 15;280(3):300-2. doi: 10.1001/jama.280.3.300. PMID: 9676691.	1998	Did not assess HA issues as defined in our protocol
Feeser VR, Simon JR. The ethical assignment of authorship in scientific publications: issues and guidelines. Acad Emerg Med. 2008 Oct;15(10):963-9. doi: 10.1111/j.1553-2712.2008.00239.x. Epub 2008 Sep 17. PMID: 18801021.	2008	not a survey
Ivanis A, Hren D, Sambunjak D, Marusić M, Marusić A. Quantification of authors' contributions and eligibility for authorship: randomized study in a general medical journal. J Gen Intern Med. 2008;23(9):1303-1310. doi:10.1007/s11606-008-0599-8	2008	Duplicate. Did not assess HA issues as defined in our protocol
O'Brien J, Baerlocher MO, Newton M, Gautam T, Noble J. Honorary coauthorship: does it matter? Can Assoc Radiol J. 2009 Dec;60(5):231-6. doi: 10.1016/j.carj.2009.09.001. Epub 2009 Oct 9. PMID: 19819102.	2009	Did not assess HA issues as defined in our protocol
Joubert G. Authorship: practices and experiences in the Faculty of Health Sciences of the University of the Free State. SA Fam Pract 2005;47(4): 57-60)	2005	Did not assess HA issues as defined in our protocol

Additional item L. Rating the overall confidence in the results of a survey

A total of 67 results were obtained, of which 63 results were included in 7 meta-analyses when same questions were asked, i.e., Question 2a (n=10 results). 'Is there a senior member of your department (including section chief or department head) who is automatically listed as an author in all submitted manuscripts?', Question 2b (n=3 results). 'Is your section or department head automatically listed as an author in all submitted manuscripts?', and Question 3a (n=50 results) 'If so, do you feel that this is justified?'. The justifications regarding the practice of researchers reporting the practice of automatically listing a senior member(s) of their department (including section chief or department head) as an author on all submitted articles were divided among 5 answers, i.e., 'never justified' (n=10 results), 'rarely justified' (n= 10 results), 'sometimes justified' (n= 10 results), 'most of the time justified' (n= 10 results), and 'always justified' (n=10 results). The results for each of these 5 justifications were quantitatively synthesized in separate meta-analyses with 10 results for each justification. The remaining 4 results of this systematic review came from single unique questions that could not be meta-analyzed, because these questions were only asked once. The ratings of the overall confidence in each result based on the seven critical items (Items 2, 5, 6, 7, 8, 12, and 13) of the 14-item quality checklist are given in Appendix Table A23.

Appendix Table A23. Rating the overall confidence in the results of a survey*

Reference	Survey question	Item 2	Item 5	Item 6	Item 7	Item 8	Item 12	Item 13	Overall confidence in the result
Bonekamp 2012	Is there a senior member of your department, including your section chief or department head, who is automatically listed as an author in all submitted manuscripts?	?	?	😊	?	😊	🙁	😊	low
Bonekamp 2012	If so, do you feel that this is justified? Answer: ‘never justified’	?	?	😊	?	😊	🙁	😊	low
Bonekamp 2012	If so, do you feel that this is justified? Answer: ‘rarely justified’	?	?	😊	?	😊	🙁	😊	low
Bonekamp 2012	If so, do you feel that this is justified? Answer: ‘sometimes justified’	?	?	😊	?	😊	🙁	😊	low
Bonekamp 2012	If so, do you feel that this is justified? Answer: ‘most of the time justified’	?	?	😊	?	😊	🙁	😊	low
Bonekamp 2012	If so, do you feel that this is justified? Answer: ‘always justified’	?	?	😊	?	😊	🙁	😊	low
Eisenberg 2011	Is your section or department head automatically listed as an author in all submitted manuscripts?	?	?	😊	?	😊	🙁	😊	low
Eisenberg 2011	If so, do you feel that this is justified in all cases?	?	?	😊	?	😊	🙁	😊	low
Eisenberg 2014	Is your section or department head automatically listed as an author in all submitted manuscripts?	?	?	😊	?	😊	🙁	😊	low
Eisenberg 2018	Is your section or department head automatically listed as an author in all submitted manuscripts?	?	?	😊	?	😊	🙁	🙁	critically low
Gadjradj 2018	Is there a senior member of your department, including section chief or department head, who is automatically listed as an author in all submitted manuscripts?	?	?	😊	?	😊	🙁	😊	low
Gadjradj 2018	If so, do you feel that this is justified? Answer: ‘never justified’	?	?	😊	?	😊	🙁	🙁	critically low
Gadjradj 2018	If so, do you feel that this is justified? Answer: ‘rarely justified’	?	?	😊	?	😊	🙁	🙁	critically low
Gadjradj 2018	If so, do you feel that this is justified? Answer: ‘sometimes justified’	?	?	😊	?	😊	🙁	🙁	critically low
Gadjradj 2018	If so, do you feel that this is justified? Answer: ‘most of the time justified’	?	?	😊	?	😊	🙁	😊	low
Gadjradj 2018	If so, do you feel that this is justified? Answer: ‘always justified’	?	?	😊	?	😊	🙁	😊	low
Gadjradj 2020	Is there a senior member of your department, including section chief or department head, who is automatically listed as an author in all submitted manuscripts?	?	?	😊	?	😊	🙁	😊	low
Gadjradj 2020	If so, do you feel that this is justified? Answer: ‘never justified’	?	?	😊	?	😊	🙁	🙁	critically low
Gadjradj 2020	If so, do you feel that this is justified? Answer: ‘rarely justified’	?	?	😊	?	😊	🙁	🙁	critically low
Gadjradj 2020	If so, do you feel that this is justified? Answer: ‘sometimes justified’	?	?	😊	?	😊	🙁	🙁	critically low
Gadjradj 2020	If so, do you feel that this is justified? Answer: ‘most of the time justified’	?	?	😊	?	😊	🙁	🙁	critically low
Gadjradj 2020	If so, do you feel that this is justified? Answer: ‘always justified’	?	?	😊	?	😊	🙁	😊	low
Gadjradj 2021	Is there a senior member of your department, including section chief or department head, who is automatically listed as an author in all submitted manuscripts?	?	?	😊	?	😊	🙁	😊	low
Gadjradj 2021	If so, do you feel that this is justified? Answer: ‘never justified’	?	?	😊	?	😊	🙁	🙁	critically low
Gadjradj 2021	If so, do you feel that this is justified? Answer: ‘rarely justified’	?	?	😊	?	😊	🙁	🙁	critically low
Gadjradj 2021	If so, do you feel that this is justified? Answer: ‘sometimes justified’	?	?	😊	?	😊	🙁	🙁	critically low
Gadjradj 2021	If so, do you feel that this is justified? Answer: ‘most of the time justified’	?	?	😊	?	😊	🙁	😊	critically low
Gadjradj 2021	If so, do you feel that this is justified? Answer: ‘always justified’	?	?	😊	?	😊	🙁	😊	low
Gülen 2020	Is there a senior member of your department, including your section chief or department head, who is automatically listed as a coauthor in the review without fulfilling the ICMJE criteria for authorship?	?	🙁	😊	?	😊	😊	😊	low
Hardjosantoso 2020	Is there a senior member of your department, including section chief or department head, who is automatically listed as an author in all submitted manuscripts?	?	🙁	😊	?	😊	🙁	😊	critically low

Hardjosantoso 2020	If so, do you feel that this is justified? Answer: ‘never justified’	?	☹	😊	?	😊	☹	☹	critically low
Hardjosantoso 2020	If so, do you feel that this is justified? Answer: ‘rarely justified’	?	☹	😊	?	😊	☹	☹	critically low
Hardjosantoso 2020	If so, do you feel that this is justified? Answer: ‘sometimes justified’	?	☹	😊	?	😊	☹	☹	critically low
Hardjosantoso 2020	If so, do you feel that this is justified? Answer: ‘most of the time justified’	?	☹	😊	?	😊	☹	☹	critically low
Hardjosantoso 2020	If so, do you feel that this is justified? Answer: ‘always justified’	?	☹	😊	?	😊	☹	☹	critically low
Kayapa 2018	Is there a senior member of your department, including section chief or department head, who is automatically listed as an author in all submitted manuscripts?	?	☹	😊	?	😊	☹	😊	critically low
Kayapa 2018	If so, do you feel that this is justified? Answer: ‘never justified’	?	☹	😊	?	😊	☹	☹	critically low
Kayapa 2018	If so, do you feel that this is justified? Answer: ‘rarely justified’	?	☹	😊	?	😊	☹	☹	critically low
Kayapa 2018	If so, do you feel that this is justified? Answer: ‘sometimes justified’	?	☹	😊	?	😊	☹	☹	critically low
Kayapa 2018	If so, do you feel that this is justified? Answer: ‘most of the time justified’	?	☹	😊	?	😊	☹	😊	low
Kayapa 2018	If so, do you feel that this is justified? Answer: ‘always justified’	?	☹	😊	?	😊	☹	😊	low
Luiten 2019	Is there a senior member of your department, who is automatically listed as an author in all submitted manuscripts?	?	?	😊	?	😊	☹	😊	low
Luiten 2019	If so, do you feel that this is justified? Answer: ‘never justified’	?	?	😊	?	😊	☹	☹	critically low
Luiten 2019	If so, do you feel that this is justified? Answer: ‘rarely justified’	?	?	😊	?	😊	☹	☹	critically low
Luiten 2019	If so, do you feel that this is justified? Answer: ‘sometimes justified’	?	?	😊	?	😊	☹	☹	critically low
Luiten 2019	If so, do you feel that this is justified? Answer: ‘most of the time justified’	?	?	😊	?	😊	☹	😊	low
Luiten 2019	If so, do you feel that this is justified? Answer: ‘always justified’	?	?	😊	?	😊	☹	😊	low
Matawlie 2021	Is there a senior member of your department, including section chief or department head, who is automatically listed as an author in all submitted manuscripts?	?	?	😊	?	😊	☹	😊	low
Matawlie 2021	If so, do you feel that this is justified? Answer: ‘never justified’	?	?	😊	?	😊	☹	☹	critically low
Matawlie 2021	If so, do you feel that this is justified? Answer: ‘rarely justified’	?	?	😊	?	😊	☹	☹	critically low
Matawlie 2021	If so, do you feel that this is justified? Answer: ‘sometimes justified’	?	?	😊	?	😊	☹	☹	critically low
Matawlie 2021	If so, do you feel that this is justified? Answer: ‘most of the time justified’	?	?	😊	?	😊	☹	😊	low
Matawlie 2021	If so, do you feel that this is justified? Answer: ‘always justified’	?	?	😊	?	😊	☹	😊	low
Noruzi 2019	Is there a senior member of your department, including section chief or department head, who is automatically listed as an author in all submitted manuscripts?	?	?	😊	?	😊	☹	😊	low
Noruzi 2019	If so, do you feel that this is justified? Answer: ‘never justified’	?	?	😊	?	😊	☹	😊	low
Noruzi 2019	If so, do you feel that this is justified? Answer: ‘rarely justified’	?	?	😊	?	😊	☹	😊	low
Noruzi 2019	If so, do you feel that this is justified? Answer: ‘sometimes justified’	?	?	😊	?	😊	☹	😊	low
Noruzi 2019	If so, do you feel that this is justified? Answer: ‘most of the time justified’	?	?	😊	?	😊	☹	😊	low
Noruzi 2019	If so, do you feel that this is justified? Answer: ‘always justified’	?	?	😊	?	😊	☹	😊	low
Nurmohamed 2021	Is there a senior member of your department, including section chief or department head, who is automatically listed as an author in all submitted manuscripts?	?	☹	😊	?	😊	☹	😊	critically low
Nurmohamed 2021	If so, do you feel that this is justified? Answer: ‘never justified’	?	☹	😊	?	😊	☹	☹	critically low
Nurmohamed 2021	If so, do you feel that this is justified? Answer: ‘rarely justified’	?	☹	😊	?	😊	☹	☹	critically low
Nurmohamed 2021	If so, do you feel that this is justified? Answer: ‘sometimes justified’	?	☹	😊	?	😊	☹	☹	critically low

Nurmohamed 2021	If so, do you feel that this is justified? Answer: 'most of the time justified'	?	☹️	😊	?	😊	☹️	☹️	critically low
Nurmohamed 2021	If so, do you feel that this is justified? Answer: 'always justified'	?	☹️	😊	?	😊	☹️	☹️	critically low
Rajasekaran 2014	Is there a senior member of your department, including your section chief or department head, who is automatically listed as a coauthor on all of your submitted manuscripts?	?	☹️	😊	?	😊	☹️	😊	critically low
Rajasekaran 2014	If yes, do you feel that this is justified in all cases?	?	☹️	😊	?	😊	☹️	☹️	critically low
Total ?		67	46	0	67	0	0	0	
Total 😊		0	0	67	0	67	1	36	
Total ☹️		0	21	0	0	0	66	31	

? = unclear risk of bias
😊 = low risk of bias
☹️ = high risk of bias
*The quality items scored in Appendix Table A23 "Rating the overall confidence in the results of a survey" are listed in this table and are further explained in Additional item F.
Quality item 2: Selective (non) reporting regarding review item (#)
Quality item 5: Risk of bias associated with the survey methods
Quality item 6: Defining the characteristics of the responding surveyees
Quality item 7: Characteristics of the responding surveyees on the review item representative for the target population?
Quality item 8: Defining the review item
Quality item 12: Magnitude of the response rate on the review item
Quality item 13: Magnitude of the sample size

The overall confidence in the 67 results in the 15 eligible surveys was rated as either 'low' (n= 31) or 'critically low' (n= 36). The prevalence of the answers to the 7 'critical items of the 14-item quality checklist was given in Appendix Table A24. The characteristics of the respondents (item 6), and the review items (Item 8) were defined in all surveys. However, whether the characteristics of the respondents were representative for the target population (item 7) was unclear, often caused by partial or poor reporting on socio-demographics and non-reporting on the characteristics of non-responders. The low-quality ratings were predominantly the result of shortcomings in the survey methods (31% (21/67), low response rates (99% (66/67), and inadequate sample sizes (46% (31/67).

Appendix Table A24. Prevalence of answers to the seven critical items of the quality checklist

**Quality checklist item and number**	**Unclear***	**Yes**	**No**
Selective (non) reporting regarding review item (#)? (Item 2)	100% (67/67)		0%
Were there no survey methods that could have introduced bias? (Item 5)	69% (46/67)	0%	31% (21/67)
Were the characteristics of the responding surveyees on the review item defined? (Item 6)	0%	100% (67/67)	0%
Were the characteristics of the responding surveyees on the review item representative for the target population (Item 7)	100% (67/67)	0%	0%
Was the review item defined? (Item 8)	0%	100% (67/67)	0%
Did the magnitude of the response rate on the review item or the way the response rate (in the case of a low response rate) was managed provide certainty in the validity of the results on the review item (Item 12)	0%	1% (1/67)	99% (66/67)
Was the sample size adequate for the prevalence statistic of the review item? (Item 13)**	0%	54% (36/67)	46% (31/67)

*'Unclear' was assigned when too few details were reported in the manuscript or additional files to make a judgment of assigning 'Yes' or 'No'.
** the required sample size was calculated with EpiTools epidemiological calculators and was based on the identified prevalence and the total sample size [12]. The estimated prevalence was calculated with a 0.95 confidence level (desired precision of estimate 0.05).

Additional item M. Response rates and results of survey questions

In this section we first reported the response rates and the results for each survey question and categorized them according to 'same research question asked' or 'unique research question asked'. At the end of this section, we report all outcomes in summary tables.

Results for review item 1

No survey item addressed review item 1, i.e., 'Researchers reporting the practice of listing a senior member(s) of a department, who did not qualify for authorship, as co-author(s) on all or most submitted articles by default.

Results of multiple surveys using the same research question for review item 2

Appendix Tables A25 and A26 report the results of multiple surveys that addressed either Question 2a or 2b. The results for each of these 2 questions were meta-analyzed and were reported at the end of this section in summary tables.

Appendix Table A25. Review Item 2a. Researchers reporting the practice of automatically listing a senior member(s) of their department (including section chief or department head) as an author on all submitted articles.
Question 2a. Is there a senior member of your department (including section chief or department head) who is automatically listed as an author in all submitted manuscripts?

Author	Year of publication	Numerator response rate	Denominator response rate (type of denominator)*	Response rate	Numerator review item	Denominator review item	Prevalence review item
Bonekamp	2012	490	1,179 (N2)	0.41560645	99	490	0.202041
Gadjradj	2018	354	1,143 (N2)	0.30971129	71	354	0.200565
Gadjradj	2020	284	1,180 (N2)	0.24067797	72	284	0.253521
Gadjradj	2021	226	914 (N2)	0.24726477	56	226	0.247788
Hardjosantoso	2020	329	1,688 (N1)	0.19490521	56	329	0.170213
Kayapa	2018	341	1,359 (N1)	0.25091979	63	341	0.184751
Luiten	2019	307	1,037 (N3)	0.29604629	56	307	0.18241
Matawlie	2021	226	1,051 (N1)	0.2150333	30	226	0.132743
Noruzi	2019	583	1,511 (N3)	0.38583719	201	583	0.344768
Nurmohamed	2021	479	1,392 (N2)	0.3441092	61	479	0.127349

*N1: Number of emails with questionnaires sent, N2: Number of emails with questionnaires not bounced, N3: Number of questionnaires for which the surveyee was available

Appendix Table A26. Review Item 2b. Researchers reporting the practice of automatically listing their section or department head as an author on all submitted articles.
Question 2b. Is your section or department head automatically listed as an author in all submitted manuscripts?

Author	Year of publication	Numerator response rate	Denominator response rate (type of denominator)*	Response rate	Numerator review item	Denominator review item	Prevalence review item
Eisenberg	2011	383	1,338 (N1)	0.28624813	96	383	0.250653
Eisenberg	2014	328	1,337 (N2)	0.24532536	83	328	0.253049
Eisenberg	2018	309	1,839 (N3)	0.1680261	73	309	0.236246

*N1: Number of emails with questionnaires sent, N2: Number of emails with questionnaires not bounced, N3: Number of questionnaires for which the surveyee was available

Results of single surveys using a unique research question for review item 2.

Appendix Tables A27 and A28 report the results of single surveys using a unique research question for review item 2 that was not assessed in the other eligible surveys.

Appendix Table A27. Review Item 2c. Researchers reporting the practice of automatically listing a senior member(s) of their department, including their section chief or department head, as a co-author on a manuscript without fulfilling the ICMJE criteria for authorship.*

Question 2c. Is there a senior member of your department, including your section chief or department head, who is automatically listed as a coauthor in the review without fulfilling the ICMJE criteria for authorship?

Author	Year of publication	Numerator response rate	Denominator response rate (type of denominator)**	Response rate	Numerator review item	Denominator review item	Prevalence review item
Gülen	2020	666	1,221 (N3)	0.54545	45	666	0.067568

*The results of single survey question 2c could not be combined with the meta-analyzed results for question 2a, because question 2c referred to fulfilling the ICMJE criteria for authorship and question 2a did not refer to specific criteria for authorship.

**N3: Number of questionnaires for which the surveyee was available.

Appendix Table A28. Review Item 2d. Researchers reporting the practice of automatically listing a senior member(s) of their department, including their section chief or department head, as a co-author(s) on all articles submitted by these researchers.*

Question 2d. Is there a senior member of your department, including your section chief or department head, who is automatically listed as a coauthor on all of your submitted manuscripts?

Author	Year of publication	Numerator response rate	Denominator response rate (type of denominator)**	Response rate	Numerator review item	Denominator review item	Prevalence review item
Rajasekaran	2014	247	908 (N3)	0.272026	31	247	0.125506

*The results of single survey question 2d could not be combined with the meta-analyzed results for question 2a, because question 2d referred specifically to 'all of your submitted manuscripts', while question 2a referred to 'all submitted manuscripts'.

**N3: Number of questionnaires for which the surveyee was available.

Results of multiple surveys using the same research question for review item 3

Appendix Tables A29-A33 report the results of multiple surveys on the various justifications ('never justified', 'rarely justified', 'sometimes justified', 'most of the time justified', 'always justified') of the practice reported in question 2a **'**Is there a senior member of your department, including section chief or department head, who is automatically listed as an author in all submitted manuscripts?' The results were meta-analyzed and were reported at the end of this section in summary tables.

Appendix Table A29. Review item 3a. Justification **'never justified'** for review item 2a, i.e., researchers reporting the practice of automatically listing a senior member(s) of their department (including section chief or department head) as an author on all submitted articles.

Question review item 3a: If so, do you feel this is justified? **Answer: 'Never justified'**

**Author**	**Year of publication**	**Numerator response rate**	**Denominator response rate (type of denominator)***	**Response rate**	**Numerator (never justified)**	**Denominator (never justified)**	**Prevalence (never justified)**
Bonekamp	2012	490	1,179 (N2)	0.415606	181	490	0.369388
Gadjradj	2018	250	1,143 (N2)	0.218723	84	250	0.336
Gadjradj	2020	195	1,180 (N2)	0.165254	49	195	0.251282
Gadjradj	2021	157	914 (N2)	0.171772	47	157	0.299363
Hardjosantoso	2020	58	1,688 (N1)	0.03436	8	58	0.137931
Kayapa	2018	199	1,359 (N1)	0.146431	72	199	0.361809
Luiten	2019	190	1,037 (N3)	0.183221	58	190	0.305263
Matawlie	2021	136	1,051 (N1)	0.129401	55	136	0.404412
Noruzi	2019	444	1,511 (N3)	0.293845	87	444	0.195946
Nurmohamed	2021	61	1,392 (N2)	0.043822	6	61	0.098361

*N1: Number of emails with questionnaires sent, N2: Number of emails with questionnaires not bounced, N3: Number of questionnaires for which the surveyee was available

Appendix Table A30. Review item 3a. Justification **'rarely justified'** for review item 2a, i.e., researchers reporting the practice of automatically listing a senior member(s) of their department (including section chief or department head) as an author on all submitted articles.

Question review item 3a: If so, do you feel this is justified? **Answer: 'Rarely justified'**

**Author**	**Year of publication**	**Numerator response rate**	**Denominator response rate (type of denominator)***	**Response rate**	**Numerator (rarely justified)**	**Denominator (rarely justified)**	**Prevalence (rarely justified)**
Bonekamp	2012	490	1,179 (N2)	0.415606	129	490	0.263265
Gadjradj	2018	250	1,143 (N2)	0.218723	66	250	0.264
Gadjradj	2020	195	1,180 (N2)	0.165254	38	195	0.194872
Gadjradj	2021	157	914 (N2)	0.171772	33	157	0.210191
Hardjosantoso	2020	58	1,688 (N1)	0.03436	13	58	0.224138
Kayapa	2018	199	1,359 (N1)	0.146431	49	199	0.246231
Luiten	2019	190	1,037 (N3)	0.183221	57	190	0.3
Matawlie	2021	136	1,051 (N1)	0.129401	40	136	0.294118
Noruzi	2019	444	1,511 (N3)	0.293845	105	444	0.236486
Nurmohamed	2021	61	1,392 (N2)	0.043822	10	61	0.163934

*N1: Number of emails with questionnaires sent, N2: Number of emails with questionnaires not bounced, N3: Number of questionnaires for which the surveyee was available

Appendix Table A31. Review item 3a. Justification **'sometimes justified'** for review item 2a, i.e., researchers reporting the practice of automatically listing a senior member(s) of their department (including section chief or department head) as an author on all submitted articles.

Question review item 3a: If so, do you feel this is justified? **Answer: 'Sometimes justified'**

Author	Year of publication	Numerator response rate	Denominator response rate (type of denominator)*	Response rate	Numerator (sometimes justified)	Denominator (sometimes justified)	Prevalence (sometimes justified)
Bonekamp	2012	490	1,179 (N2)	0.415606	131	490	0.267347
Gadjradj	2018	250	1,143 (N2)	0.218723	64	250	0.256
Gadjradj	2020	195	1,180 (N2)	0.165254	56	195	0.287179
Gadjradj	2021	157	914 (N2)	0.171772	43	157	0.273885
Hardjosantoso	2020	58	1,688 (N1)	0.03436	14	58	0.241379
Kayapa	2018	199	1,359 (N1)	0.146431	40	199	0.201005
Luiten	2019	190	1,037 (N3)	0.183221	49	190	0.257895
Matawlie	2021	136	1,051 (N1)	0.129401	23	136	0.169118
Noruzi	2019	444	1,511 (N3)	0.293845	131	444	0.295045
Nurmohamed	2021	61	1,392 (N2)	0.043822	15	61	0.245902

*N1: Number of emails with questionnaires sent, N2: Number of emails with questionnaires not bounced, N3: Number of questionnaires for which the surveyee was available

Appendix Table A32. Review item 3a. Justification **'most of the time justified'** for review item 2a, i.e., researchers reporting the practice of automatically listing a senior member(s) of their department (including section chief or department head) as an author on all submitted articles.

Question review item 3a: If so, do you feel this is justified? **Answer: 'Most of the time justified'**

Author	Year of publication	Numerator response rate	Denominator response rate (type of denominator)*	Response rate	Numerator (most of the time justified)	Denominator (most of the time justified)	Prevalence (most of the time justified)
Bonekamp	2012	490	1,179 (N2)	0.415606	24	490	0.04898
Gadjradj	2018	250	1,143 (N2)	0.218723	22	250	0.088
Gadjradj	2020	195	1,180 (N2)	0.165254	35	195	0.179487
Gadjradj	2021	157	914 (N2)	0.171772	18	157	0.11465
Hardjosantoso	2020	58	1,688 (N1)	0.03436	12	58	0.206897
Kayapa	2018	199	1,359 (N1)	0.146431	24	199	0.120603
Luiten	2019	190	1,037 (N3)	0.183221	16	190	0.084211
Matawlie	2021	136	1,051 (N1)	0.129401	12	136	0.088235
Noruzi	2019	444	1,511 (N3)	0.293845	81	444	0.182432
Nurmohamed	2021	61	1,392 (N2)	0.043822	17	61	0.278689

*N1: Number of emails with questionnaires sent, N2: Number of emails with questionnaires not bounced, N3: Number of questionnaires for which the surveyee was available

Appendix Table A33. Review item 3a. Justification **'Always justified'** for review item 2a, i.e., researchers reporting the practice of automatically listing a senior member(s) of their department (including section chief or department head) as an author on all submitted articles.

Question review item 3a: If so, do you feel this is justified? **Answer: 'Always justified'**

Author	Year of publication	Numerator response rate	Denominator response rate (type of denominator)*	Response rate	Numerator (always justified)	Denominator (always justified)	Prevalence (always justified)
Bonekamp	2012	490	1,179 (N2)	0.415606	25	490	0.05102
Gadjradj	2018	250	1,143 (N2)	0.218723	14	250	0.056
Gadjradj	2020	195	1,180 (N2)	0.165254	17	195	0.087179
Gadjradj	2021	157	914 (N2)	0.171772	16	157	0.101911
Hardjosantoso	2020	58	1,688 (N1)	0.03436	11	58	0.189655
Kayapa	2018	199	1,359 (N1)	0.146431	14	199	0.070352
Luiten	2019	190	1,037 (N3)	0.183221	10	190	0.052632
Matawlie	2021	136	1,051 (N1)	0.129401	6	136	0.044118
Noruzi	2019	444	1,511 (N3)	0.293845	40	444	0.09009
Nurmohamed	2021	61	1,392 (N2)	0.043822	13	61	0.213115

*N1: Number of emails with questionnaires sent, N2: Number of emails with questionnaires not bounced, N3: Number of questionnaires for which the surveyee was available

Results of single surveys using a unique research question for review item 3.

Appendix Tables A34 and A35 report the results of single surveys using a unique research question for review item 3 that was not assessed in the other eligible surveys.

Appendix Table A34. Review item 3b. Justification **'justified in all cases'** for review item 2b, i.e., researchers reporting the practice of automatically listing their section or department head as an author on all submitted articles.

Question review item 3b: If so, do you feel that this is justified in all cases? **Answer: 'Justified in all cases'**

Author	Year of publication	Numerator response rate	Denominator response rate (type of denominator)*	Response rate	Numerator (practice was justified)	Denominator (practice was justified)	Prevalence (practice was justified)
Eisenberg	2011	96	1,338 (N1)	0.071749	34	96	0.35417

*N1: Number of emails with questionnaires sent

Appendix Table A35. Review item 3d. Justification '**justified in all cases'** for review item 2d, i.e., researchers reporting the practice of automatically listing a senior member(s) of their department, including their section chief or department head, as a co-author(s) on all articles submitted by these researchers.

Question 3d. If yes, do you feel that this is justified in all cases? **Answer: 'Justified in all cases'**

Author	Year of publication	Numerator response rate	Denominator response rate (type of denominator)*	Response rate	Numerator review item (practice was justified)	Denominator review item (practice was justified)	Prevalence review item (practice was justified)
Rajasekaran	2014	31	908 (N3)	0.03414	21	31	0.67742

*N3: Number of questionnaires for which the surveyee was available.

Summary tables of the response rates and the results for the review items

Summary tables were created to visualize the individual and the meta-analyzed response rates and results for each review item (Appendix Table A36 and A37). We conducted meta-analyses (random effects model) for the results of each review item when more than one same question was asked for the pertinent review item. We conducted meta-analyses (random effects model) for response rates when same questions were asked and same denominators were used for these response rates.

Appendix Table A36. Summary Table 1. Response rates and results for review item 2

Survey items	Response rate	Prevalence of the practice reported under the review item
**Review Item 2a.** Researchers reporting the practice of automatically listing a senior member(s) of their department (including section chief or department head) as an author on all submitted articles.<br>**Question review item 2a:**<br>Is there a senior member of your department (including section chief or department head) who is automatically listed as an author in all submitted manuscripts?	22% (N1*)<br>[95% CI 19-25]<br>(4,098 surveyees in 3 surveys)<br><br>31% (N2*)<br>[95% CI 25-37]<br>(5,808 surveyees in 5 surveys)<br><br>35% (N3*)<br>[95% CI 33-36]<br>(2,548 surveyees in 2 surveys)	20%<br>[95% CI 16-25]<br>(3,619 respondents in 10 surveys)
**Review Item 2b.** Researchers reporting the practice of automatically listing their section or department head as an author on all submitted articles.<br>**Question review item 2b:**<br>Is your section or department head automatically listed as an author in all submitted manuscripts?	29% (383/1338) (N1*)<br>[95% CI 26-31]<br>(1,338 surveyees in 1survey)<br><br>29% (328/1337) (N2*)<br>[95% CI 22-27]<br>(1,337 surveyees in 1survey)<br><br>17% (309/1839) (N3*)<br>[95% CI 15-19]<br>(1,839 surveyees in 1survey)	25%<br>[95% CI 22-27]<br>(1,020 respondents in 3 surveys)
**Review Item 2c.** Researchers reporting the practice of automatically listing a senior member(s) of their department, including their section chief or department head, as a co-author on a manuscript without fulfilling the ICMJE criteria for authorship.<br>**Question review item 2c:**<br>Is there a senior member of your department, including your section chief or department head, who is automatically listed as a coauthor in the review without fulfilling the ICMJE criteria for authorship?	54.5% (666/1221) (N3*)<br>[95% CI 51.7-57.4]<br>(1,221 surveyees in 1 survey) [22]	6.8% (45/666)<br>[95% CI 5-8.9]<br>(666 respondents in 1 survey) [22]
**Review Item 2d.** Researchers reporting the practice of automatically listing a senior member(s) of their department, including their section chief or department head, as a co-author(s) on all articles submitted by these researchers.<br>**Question review item 2d:**<br>Is there a senior member of your department, including your section chief or department head, who is automatically listed as a coauthor on all of your submitted manuscripts?	27.2% (247/908) (N3*)<br>[95% CI 24.3-30.2]<br>(908 surveyees in 1 survey) [23]	12.6% (31/247)<br>[95% CI 8.7-17.4]<br>(247 respondents in 1 survey) [23]

*N1: Number of emails with questionnaires sent, N2: Number of emails with questionnaires not bounced, N3: Number of questionnaires for which the surveyee was available

Appendix Table A37. Summary Table2. Response rates and results for review item 3

Survey items	Response rate	Prevalence of the justification 'never justified'	Prevalence of the justification 'rarely justified'	Prevalence of the justification 'sometimes justified'	Prevalence of the justification 'most of the time justified'	Prevalence of the justification 'always justified'
**Review item 3a**. Justification of review item 2a, i.e., Researchers reporting the practice of automatically listing a senior member(s) of their department (including section chief or department head) as an author on all submitted articles.<br>**Question review item 3a:**<br>If so, do you feel this is justified?	10% (N1*)<br>[95% CI 2-18]<br>(4,098 surveyees in 3 surveys)<br><br>20% (N2*)<br>[95% CI 8-32]<br>(5,808 surveyees in 5 surveys)<br><br>24% (N3*)<br>[95% CI 22-26]<br>(2,548 surveyees in 2 surveys)	**Review item 3a**<br>28%<br>[95% CI 22-34]<br>(2,180 respondents in 10 surveys)	**Review item 3a**<br>24%<br>[95% CI 22-27]<br>(2,180 respondents in 10 surveys)	**Review item 3a**<br>25%<br>[95% CI 23-28]<br>(2,180 respondents in 10 surveys)	**Review item 3a**<br>13%<br>[95% CI 9-17]<br>(2,180 respondents in 10 surveys)	**Review item 3a**<br>8%<br>[95% CI 6-9]<br>(2,180 respondents in 10 surveys)
**Review item 3b**.<br>Justification of review item 2b, i.e., Researchers reporting the practice of automatically listing their section or department head as an author on all submitted articles.<br>**Question review item 3b:**<br>If so, do you feel that this is justified in all cases?	7.2% (96/1338) (N1*)<br>[95% CI 5.9-8.7]<br>(1,338 surveyees in 1survey) [24]	Not assessed	Not assessed	Not assessed	Not assessed	**Review item 3b**<br>35.4% (34/96)<br>[95% CI 25.9-45.8]<br>(96 respondents in 1 survey) [24]<br>This outcome was assessed in 2 other surveys [25,26], but results were not published.
**Review item 3c**.<br>Justification of review item 2c, i.e.,<br>Researchers reporting the practice of automatically listing a senior member(s) of their department, including their section chief or department head, as a co-author on a manuscript without them fulfilling the ICMJE criteria for authorship.	Not assessed	Not assessed	Not assessed	Not assessed	Not assessed	Not assessed
**Review item 3d**.<br>Justification of review item 2d, i.e., Researchers reporting the practice of automatically listing a senior member(s) of their department, including their section chief or department head, as a co-author(s) on all articles submitted by these researchers.<br>**Question review item 3d:**<br>If yes, do you feel that this is **justified in all cases**?	3.4% (31/908) (N3*)<br>[95% CI 2.3-4.8]<br>(908 surveyees in 1survey) [23]	Not assessed	Not assessed	Not assessed	Not assessed	**Review item 3d**<br>67.7% (21/31)<br>[95% CI 48.6-83.3]<br>(31 respondents in 1 survey) [23]

*N1: Number of emails with questionnaires sent, N2: Number of emails with questionnaires not bounced, N3: Number of questionnaires for which the surveyee was available

Response rates

It was not always clear which denominators were used to calculate the response rates for the justification of the practice of automatically listing a senior member of a department as an author on all submitted articles (Review item 3). To avoid possible overestimation of these rates, we decided to use the same denominators for review item 3 as those used for review item 2, i.e., researchers reporting the practice of automatically listing a senior member of their department as an author on all submitted articles (Appendix Table A36 and A37). Overall, response rates for review item 3 were lower than those for review item 2.

Results of individual studies and syntheses

Researchers reporting the practice of listing a senior member(s) of a department, who did not qualify for authorship, as co-author(s) on all or most submitted articles by default (Review item 1).

No surveys addressed this review item.

Researchers reporting the practice of automatically listing (a) senior member(s) of their department (including section chief or department head) as an author on all submitted articles (Review item 2a) and the justification for this practice (Review item 3a)

Different questions were used to assess the prevalence of researchers reporting the practice of automatically listing a senior member(s) of their department as an author on all submitted articles (Review item 2) and the justification for this practice (Review item 3). We therefore divided these review items in Review item 2a and 2b and Review item 3a and 3b (Appendix Table A36 and A37).

A pooled average of 20% [95% CI 16-25] of researchers (based on data from 10 surveys, and a total of 3,619 respondents) reported that a senior member of their department (including section chief or department head) was automatically listed as an author on all submitted articles (Review item 2a)((Appendix Table A36)(Appendix Figure A2). There was strong evidence of heterogeneity (Chi^2 = 95.84 (df = 9) P<0.001) and the high I^2 (I^2 = 90.61%) indicates considerable inconsistency across the prevalence

statistics of the surveys. A smaller number of respondents (2,180) reported on the justification of this practice (Review item 3a)(Appendix Table A37), i.e., a pooled weighed average of 28% [95% CI 22-34] of researchers felt that this practice was 'never justified', 24% [95% CI 22-27] 'rarely justified', 25% [95% CI 23-28] 'sometimes justified', 13% [95% CI 9-17] 'most of the time justified', and 8% [95% CI 6-9] 'always justified' (Appendix Figures A3-7).

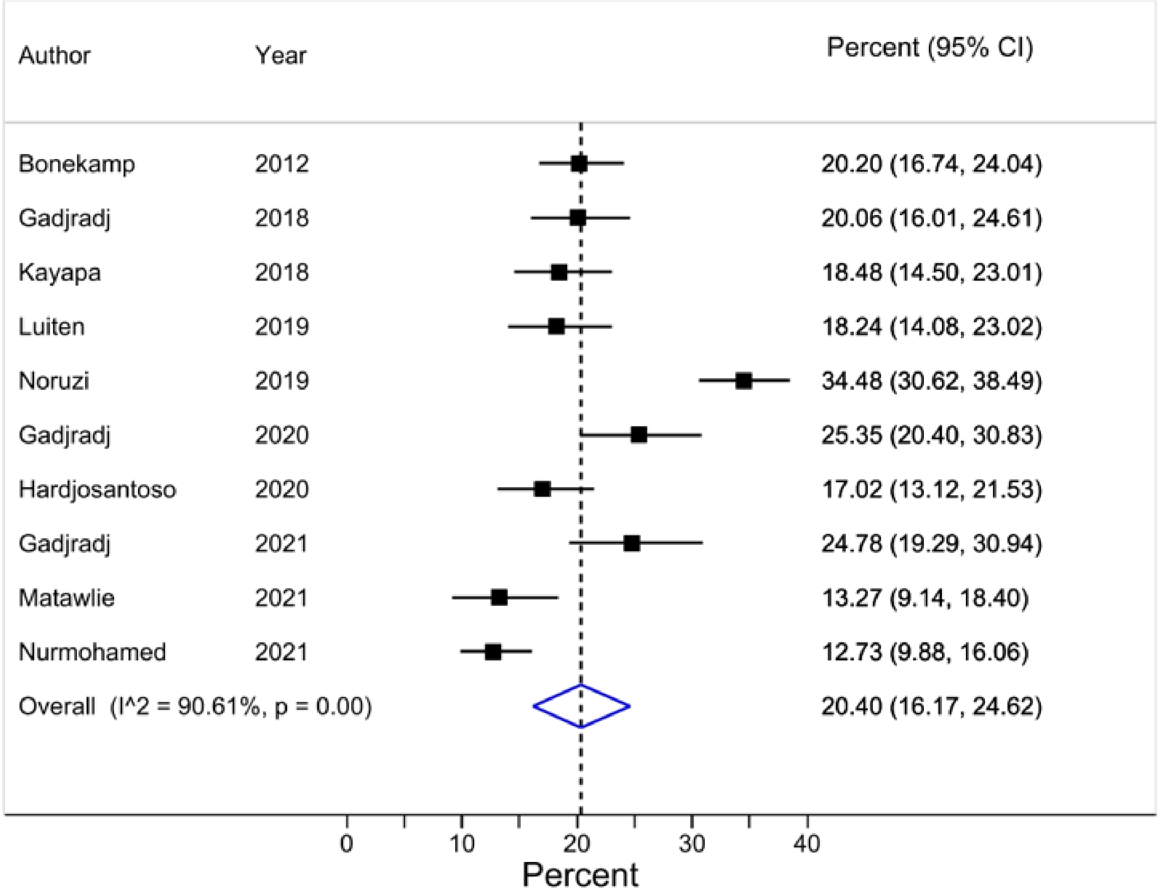

Appendix Figure A2. Forest plot for Review item 2a. Researchers reporting the practice of automatically listing a senior member(s) of their department (including section chief or department head) as an author on all submitted articles.

Effect Size (ES) 20% [95% CI 16-25]
Heterogeneity $\chi 2$ = 95.84 [df = 9]; P < .001
Variation in ES attributable to heterogeneity: I^2 = 90.61%
Between-study variance estimate τ^2 = 0.00
Test of ES=0: z= 9.47; P< .001

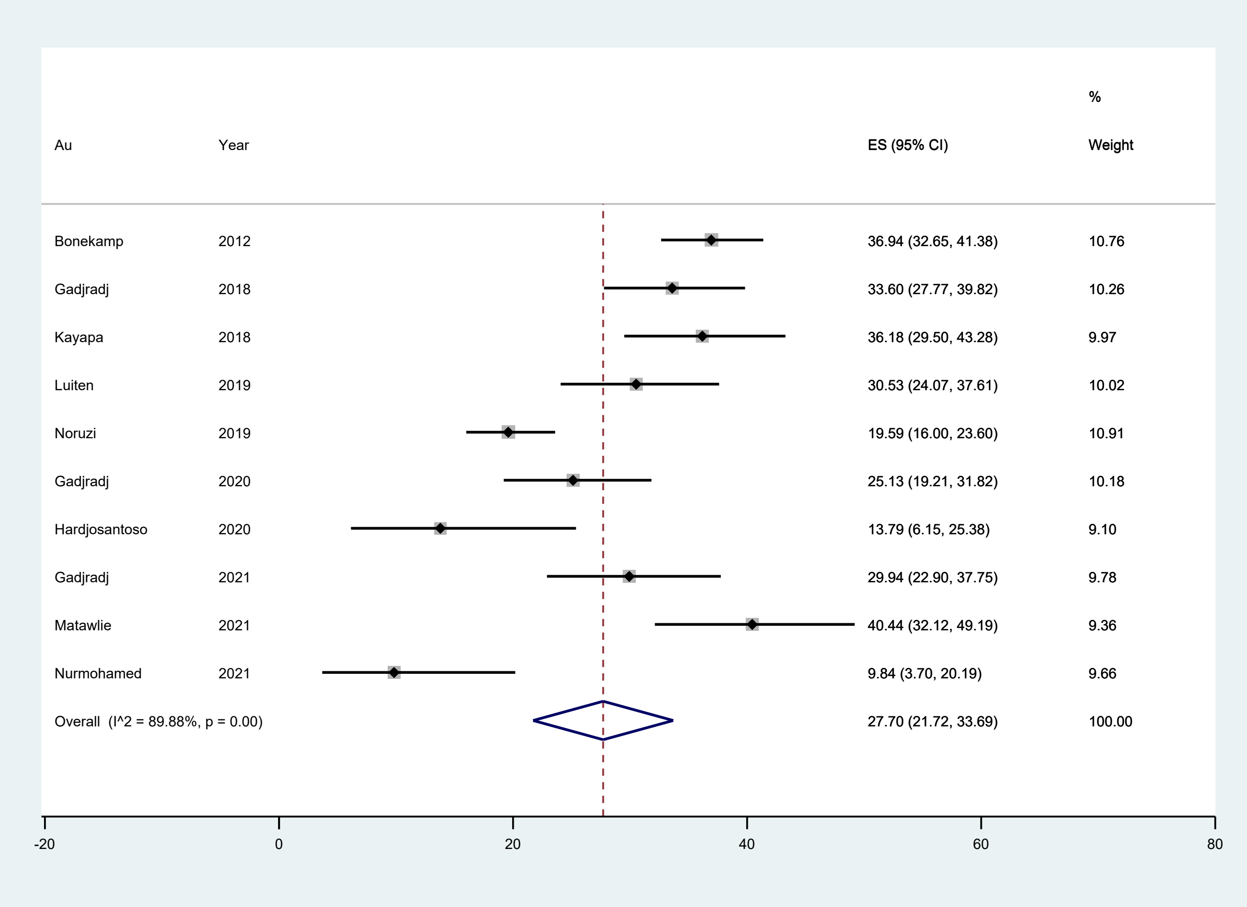

Appendix Figure A3. Forest plot for Review item 3a (justification: 'never justified'). Researchers reporting that the practice of automatically listing a senior member(s) of their department (including section chief or department head) as an author on all submitted articles is 'never justified'.

Effect Size (ES) 28% [95% CI 22-34]
Heterogeneity $\chi 2$ = 88.89 [df = 9]; P< .001
Variation in ES attributable to heterogeneity: I^2 = 89.88%
Between-study variance estimate τ^2 = 0.01
Test of ES=0: z= 9.07; P< .001

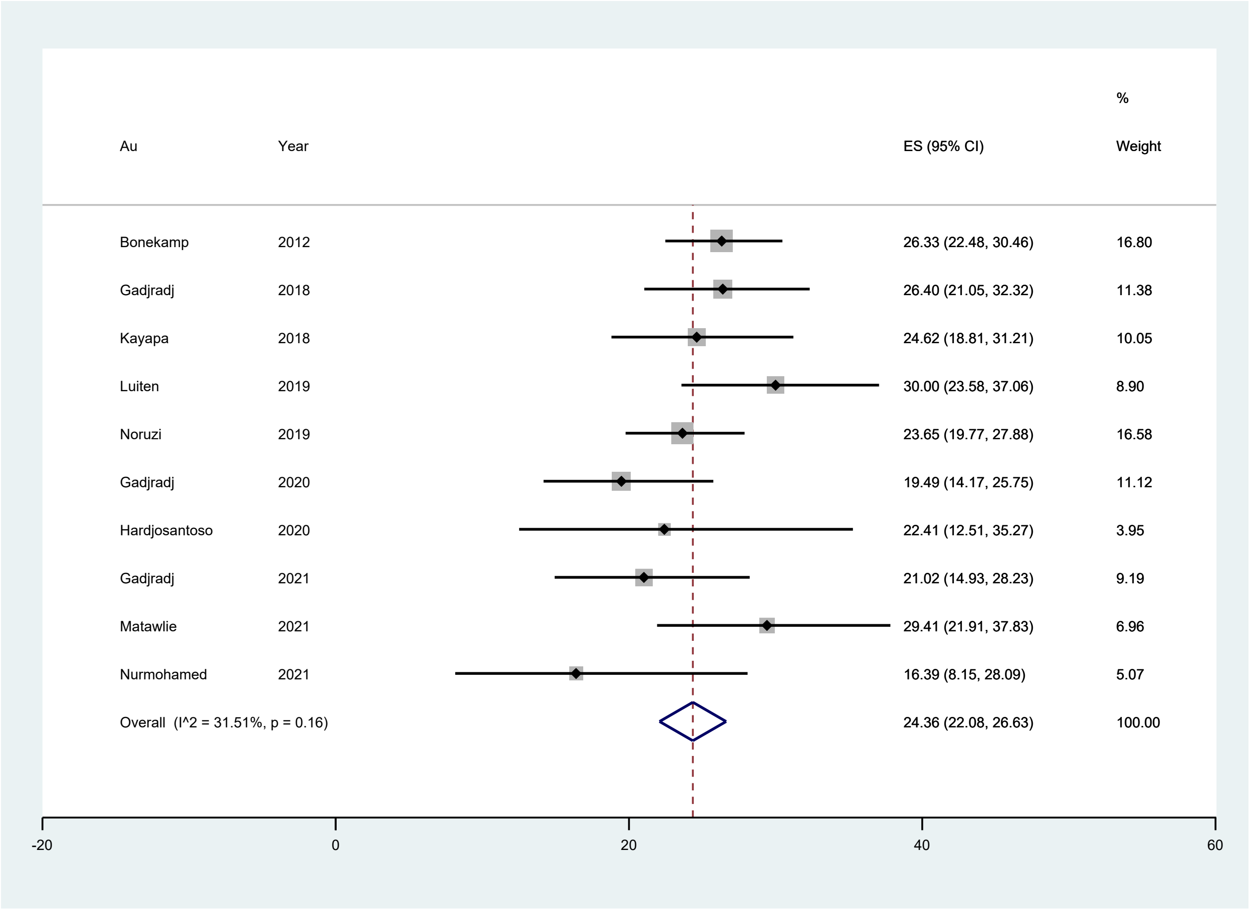

Appendix Figure A4. Forest plot for Review item 3a (justification: ‘rarely justified’). Researchers reporting that the practice of automatically listing a senior member(s) of their department (including section chief or department head) as an author on all submitted articles is ‘rarely justified’.

Effect Size (ES) 24% [95% CI 22-27]
Heterogeneity $\chi 2$ = 13.14 [df = 9]; P = 0.16
Variation in ES attributable to heterogeneity: I^2 = 31.51%
Between-study variance estimate τ^2 = 0.00
Test of ES=0: z= 21.01; P< .001

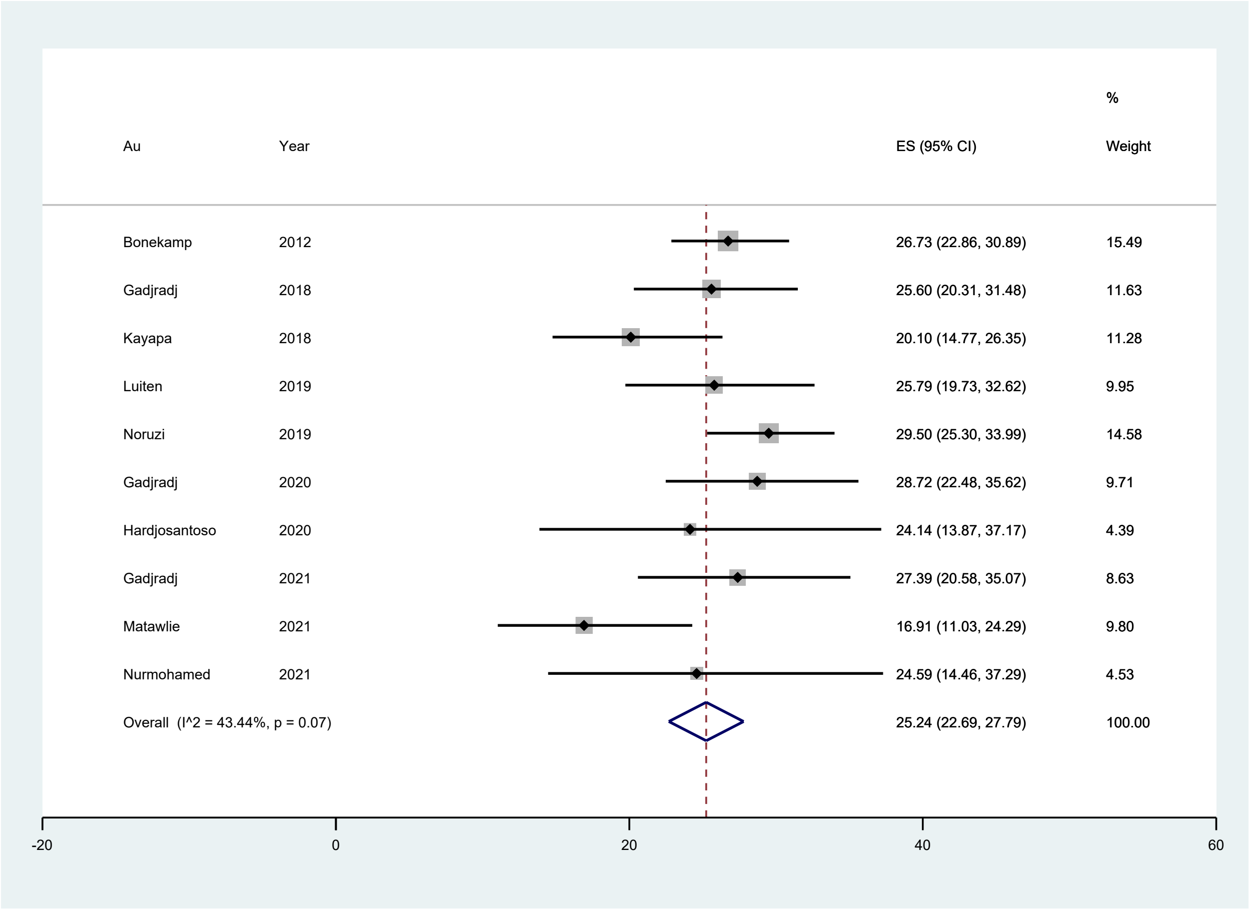

Appendix Figure A5. Forest plot for Review item 3a (justification: 'sometimes justified'). Researchers reporting that the practice of automatically listing a senior member(s) of their department (including section chief or department head) as an author on all submitted articles is 'sometimes justified'.

Effect Size (ES) 25% [95% CI 23-28]
Heterogeneity $\chi 2$ = 15.91 [df = 9]; P = 0.07
Variation in ES attributable to heterogeneity: I^2 = 43.44%
Between-study variance estimate τ^2 = 0.00
Test of ES=0: z= 19.41; P< .001

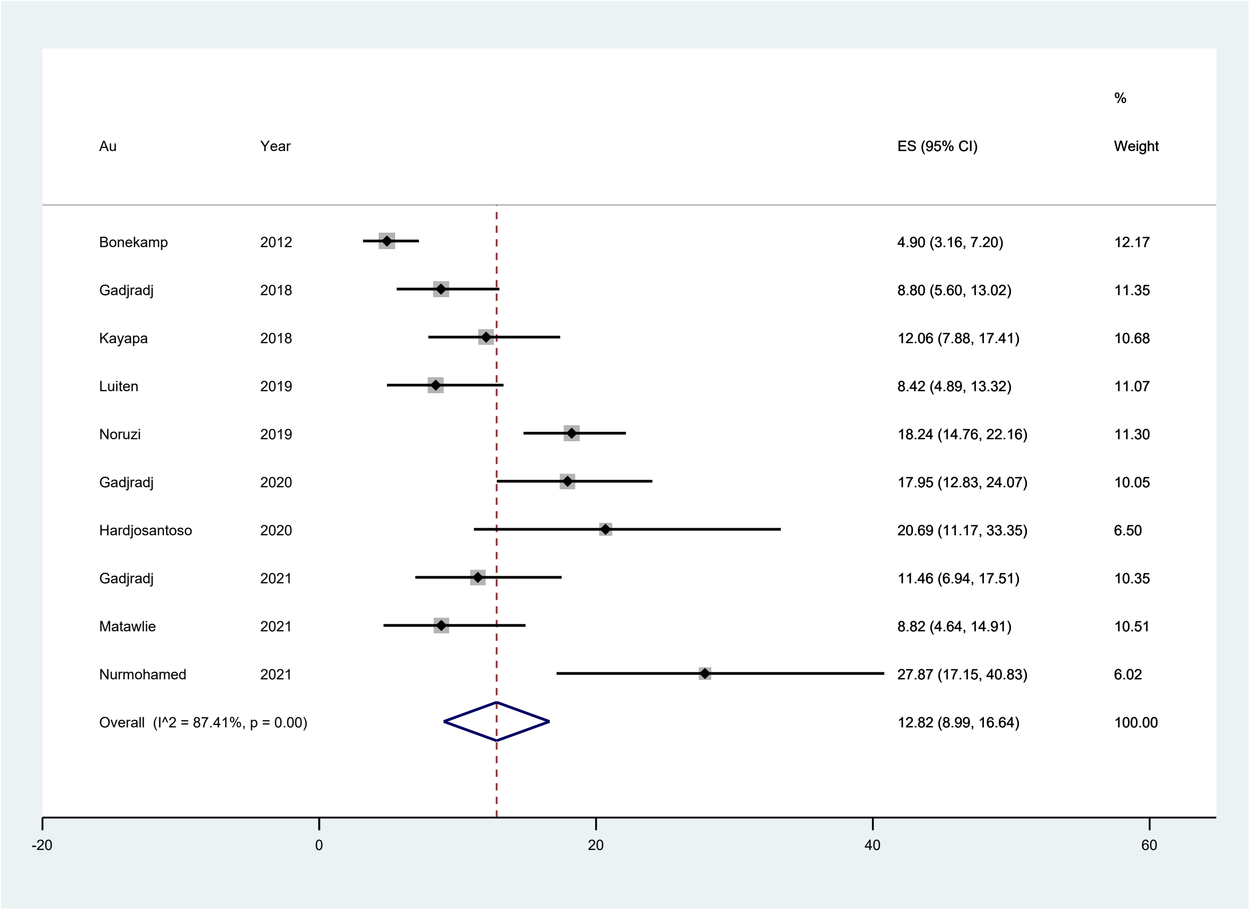

Appendix Figure A6. Forest plot for Review item 3a (justification: 'most of the time justified'). Researchers reporting that the practice of automatically listing a senior member(s) of their department (including section chief or department head) as an author on all submitted articles is 'most of the time' justified.

Effect Size (ES) 25% [95% CI 9-17]
Heterogeneity $\chi 2$ = 71.49 [df = 9]; P< .001
Variation in ES attributable to heterogeneity: I^2 = 87.41%
Between-study variance estimate τ^2 = 0.00
Test of ES=0: z= 6.57; P< .001

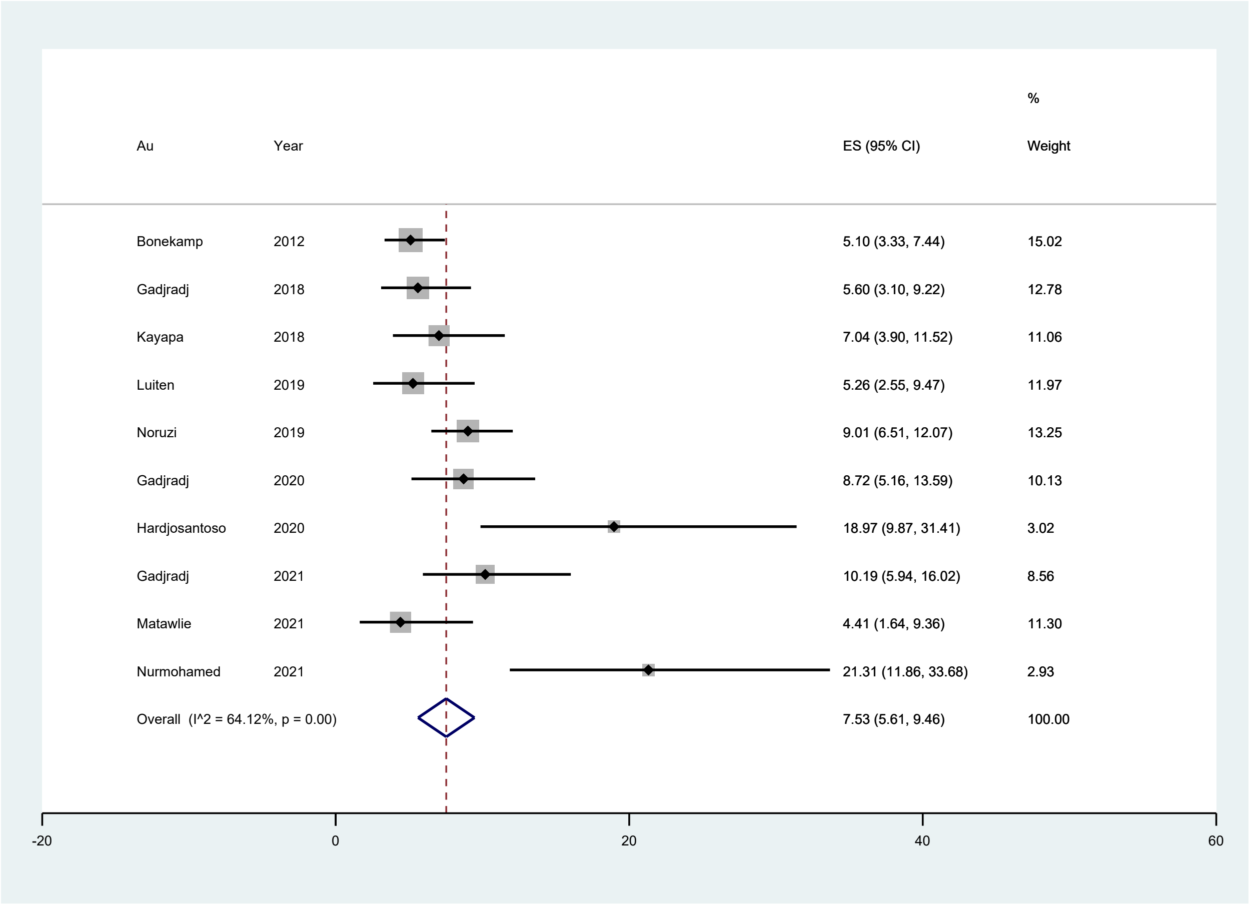

Appendix Figure A7. Forest plot for Review item 3a (justification: 'always justified'). Researchers reporting that the practice of automatically listing a senior member(s) of their department (including section chief or department head) as an author on all submitted articles is 'always' justified.

Effect Size (ES) 8% [95% CI 6-9]
Heterogeneity χ2 = 25.08 [df = 9]; P< .001
Variation in ES attributable to heterogeneity: I^2 *= 64.12%*
Between-study variance estimate τ^2 *= 0.00*
Test of ES=0: z= 7.68; P< .001

Researchers reporting the practice of automatically listing their section or department head as an author on all submitted articles (Review Item 2b) and the justification for this practice (Review item 3b)

Review item 2b (Appendix Table A37) (Appendix Figure A8) did not refer to senior members in general, but specifically to section or department heads and when pooled this led to an average of 25% [95% CI 22-27] of researchers (based on data from 3 surveys, and a total of 1,020 respondents). All three surveys were conducted in the field of radiology by the same research group [24-26]. No strong evidence for heterogeneity was found ($Chi^2 = 0.29$ (df = 2) P = 0.87) and the $I^2 = 0.00\%$ indicates high consistency across the prevalence statistics of the surveys (Appendix Figure A8). The corresponding author reported that individual survey data of all these 3 surveys were not available anymore, which did not permit the exploration of this diversity. All three surveys assessed the justification of this practice (Review item 3b), but only 1 of these surveys [24] published the results, i.e., 35.4% (34/96) of respondents felt that this practice was justified in all cases (Appendix Table A37)).

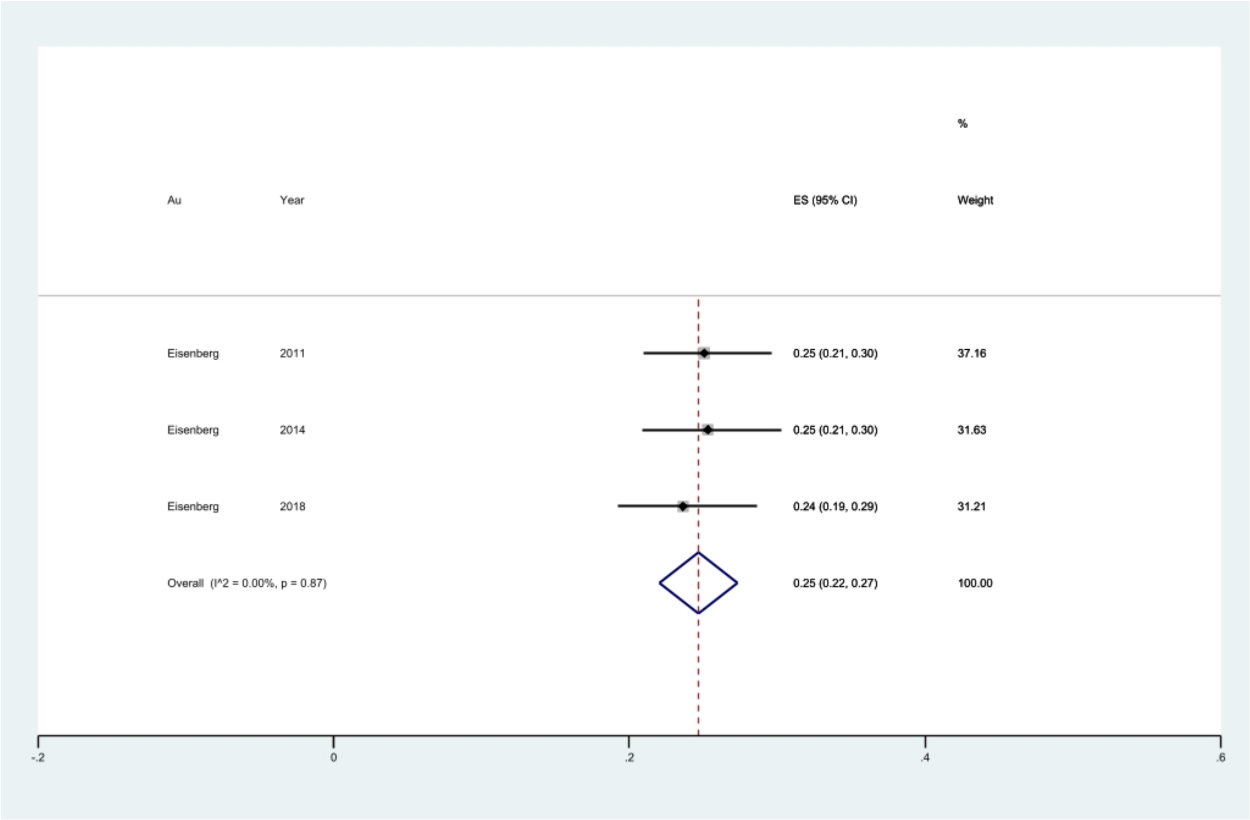

Appendix Figure A8. Forest plot for Review item 2b. Researchers reporting the practice of automatically listing their section or department head as an author on all submitted articles.

Effect Size (ES) 25% [95% CI 22-27]
Heterogeneity χ2 = 0.29 [df = 2]; P = 0.87
Variation in ES attributable to heterogeneity: $I^2 = 0.00\%$
Between-study variance estimate $\tau^2 = 0.00$
Test of ES=0: z= 18.29; P< .001

Researchers reporting the practice of automatically listing (a) senior member(s) of their department, including their section chief or department head, as a co-author on a manuscript without fulfilling the ICMJE criteria for authorship (Review Item 2c)

Review item 2c (Appendix Table A36) was only addressed in one survey [22] only and showed that 6.8% [95% CI 5-8.9] of 666 researchers reported that a senior member of their department, including their section chief or department head was automatically listed as a coauthor in a Cochrane review without fulfilling the ICMJE criteria for authorship. The justification of this practice was not assessed (Appendix Table A37). The question for this review item differed from the previous questions in that it did not refer to automatically listing of a co-author to all submitted manuscripts, but to a specific manuscript submitted by the surveyee. Further, this question referred to co-authorship without fulfilling the ICMJE criteria for authorship in Cochrane reviews.

Researchers reporting the practice of automatically listing (a) senior member(s) of their department, including their section chief or department head, as a co-author(s) on all articles submitted by these researchers (Review item 2d) and the justification of this practice (Review item 3d).

Contrary to the previous review items, review item 2d referred specifically to all manuscripts submitted by the surveyee. This review item was assessed in 1 survey [23] (Appendix Table A36), which showed that 12.6% [95% CI 8.7-17.4] of 247 researchers reported that a senior member of their department including their section chief or department head was automatically listed as a coauthor on all manuscripts submitted by the surveyee. This practice was considered ‘always justified’ by 67.7% of 31 respondents (Review item 3d) (Appendix Table A37).

Additional item N. Investigation of heterogeneity

Meta-regression

Meta-regressions were conducted for the results to questions 2a and 3a, because at least 10 observations per explanatory moderator were reported [16] and one or more of these observations differed from the other observations made. The results of these analyses as well as the explanation for the non-eligibility of explanatory moderators were listed in Appendix Tables A38-A43. For each analysis we reported the regression coefficient, the 95% confidence intervals, and the p value, e.g., (regression coefficient, XX, [95%CI XX to XX], p=XX).

For question 2a we assessed the year of publication of the survey as explanatory moderator. The year of publication of the survey (p = 0.76) had no association with the practice of automatically listing a senior member(s) of their department (including section chief or department head) as an author on all submitted articles. An association with the percentage of associate professors and higher could not be assessed, because only 5 surveys reported on this moderator.

Appendix Table A38. Moderators review Item 2a. Moderators of researchers reporting the practice of automatically listing a senior member(s) of their department (including section chief or department head) as an author on all submitted articles. **Question review item 2a:** Is there a senior member of your department (including section chief or department head) who is automatically listed as an author in all submitted manuscripts?

**Moderator**	**Eligible (yes/no)**	**# of observations**	**Rationale for non-eligibility**	**Regression coefficient**	**95% Confidence Intervals (CI), and p value**
Year of publication of the survey	Yes	10		-0.0026	95% CI -0.019%-0.014, p = 0.76
Percentage of associate professors and higher among the surveyees	No	5	Less than 10 observations		

Appendix Tables A39-A43 present the findings of the meta-regressions to assess associations of explanatory variables with the results of the justifications (‘Never justified’, ‘Rarely justified’, ‘Sometimes justified’, ‘Most of the time justified’, and ‘Always justified’) for the practice of automatically listing a senior member (s) of their department (including section chief or department head) as an author on all

submitted articles. The justification ‘most of the time justified’ increased with increasing years of publication of the survey (regression coefficient, 0.012, [95%CI: 0.00016 to 0.024], p=0.047). An association with the percentage of being at least an associate professors could not be assessed, because only 5 surveys reported on this moderator.

Appendix Table A39. Moderators review item 3a. Moderators of justification **‘never justified’** for review item 2a, i.e., researchers reporting the practice of automatically listing a senior member(s) of their department (including section chief or department head) as an author on all submitted articles.
Question review item 3a: If so, do you feel this is justified? **Answer: ‘Never justified’**

**Moderator**	**Eligible (yes/no)**	**# of observations**	**Rationale for non-eligibility**	**Regression coefficient**	**95% Confidence Intervals (CI), and p value**
Year of publication of the survey	Yes	10		-0.016	95% CI -0.038%-0.0073, p = 0.18
Percentage of associate professors and higher among the surveyees	No	5	Less than 10 observations		

Appendix Table A40. Moderators review item 3a. Moderators of justification **‘rarely justified’** for review item 2a, i.e., researchers reporting the practice of automatically listing a senior member(s) of their department (including section chief or department head) as an author on all submitted articles.
Question review item 3a: If so, do you feel this is justified? **Answer: ‘Rarely justified’**

**Moderator**	**Eligible (yes/no)**	**# of observations**	**Rationale for non-eligibility**	**Regression coefficient**	**95% Confidence Intervals (CI), and p value**
Year of publication of the survey	Yes	10		-0.0044	95% CI -0.011%-0.0026, p = 0.22
Percentage of associate professors and higher among the surveyees	No	5	Less than 10 observations		

Appendix Table A41. Moderators review item 3a. Moderators of justification **‘sometimes justified’** for review item 2a, i.e., researchers reporting the practice of automatically listing a senior member(s) of their department (including section chief or department head) as an author on all submitted articles.
Question review item 3a: If so, do you feel this is justified? **Answer: ‘Sometimes justified’**

**Moderator**	**Eligible (yes/no)**	**# of observations**	**Rationale for non-eligibility**	**Regression coefficient**	**95% Confidence Intervals (CI), and p value**
Year of publication of the survey	Yes	10		-0.0026	95% CI -0.012%-0.0072, p = 0.61
Percentage of associate professors and higher among the surveyees	No	5	Less than 10 observations		

Appendix Table A42. Moderators review item 3a. Moderators of justification **'most of the time justified'** for review item 2a, i.e., researchers reporting the practice of automatically listing a senior member(s) of their department (including section chief or department head) as an author on all submitted articles.
Question review item 3a: If so, do you feel this is justified? **Answer: 'Most of the time justified'**

Moderator	Eligible (yes/no)	# of observations	Rationale for non-eligibility	Regression coefficient	95% Confidence Intervals (CI), and p value
Year of publication of the survey	Yes	10		0.012	95% CI 0.00016%-0.024, p = 0.047*
Percentage of associate professors and higher among the surveyees	No	5	Less than 10 observations		

*P<0.05

Appendix Table A43. Moderators review item 3a. Moderators of justification **'Always justified'** for review item 2a, i.e., researchers reporting the practice of automatically listing a senior member(s) of their department (including section chief or department head) as an author on all submitted articles.
Question review item 3a: If so, do you feel this is justified? **Answer: 'Always justified'**

Moderator	Eligible (yes/no)	# of observations	Rationale for non-eligibility	Regression coefficient	95% Confidence Intervals (CI), and p value
Year of publication of the survey	Yes	10		0.0045	95% CI -0.0017%-0.011, p = 0.15
Percentage of associate professors and higher among the surveyees	No	5	Less than 10 observations		

Subgroup analyses

We conducted subgroup analyses when at least 10 observations per explanatory moderator were reported [6,16] and one or more of these observations differed from the other observations made. The following explanatory variables were assessed: 1) being a corresponding author 2) $\geq$50% of the surveyees being male 3) $\geq$50% of the surveyees being an associate professor or higher 4) the presence of risk of multiple submissions by same surveyees 5) the time frame between publication of the manuscript by the surveyee and conducting the survey was $\leq$1 year and 6) whether the survey was conducted by a research group whose' first author was affiliated with a research institute in the Netherlands or not. The results of the subgroup analyses and the rationale for non-eligibility of explanatory variables were given in Appendix Tables A44-A49. Being a corresponding author was associated with a higher prevalence (Q (1) = 6.47, p = 0.011) of researchers reporting the practice of

automatically listing a senior member(s) of their department (including section chief or department head) as an author on all submitted articles (Review item 2a).

Appendix Table A44. Moderators review Item 2a. Moderators of researchers reporting the practice of automatically listing a senior member(s) of their department (including section chief or department head) as an author on all submitted articles. **Question review item 2a:** Is there a senior member of your department (including section chief or department head) who is automatically listed as an author in all submitted manuscripts?

Variable	Eligible (yes/no)	# of observations	Rationale for non-eligibility	Test of group differences*
Netherlands country of first affiliation	Yes	10		Q (1) = 0.01, p = 0.941
Corresponding author (target population)	Yes	10		Q (1) = 6.47, p = 0.011**
$\geq$ 50% males among surveyees	No	8	Less than 10 observations	
$\geq$ 50% associate professor or higher among surveyees	No	5	Less than 10 observations	
Risk of multiple submissions by surveyees	No	6	Less than 10 observations	
Time frame $\leq$1 year between publication of manuscript and conducting the survey	No	9	Less than 10 observations	

*The chi squared test, 'Q', and the degrees of freedom (df), and the p value 'p'
**P<0.05

When the survey was conducted by a research group whose' first author was affiliated with a research institute in the Netherlands it was less likely (Q (1) = 6.68, p = 0.01) that researchers reported that the practice of automatically listing a senior member(s) of their department (including section chief or department head) as an author on all submitted articles was never justified (Review item 3a 'Never justified'). Being a corresponding author was associated with a lower prevalence (Q (1) = 7.05, p = 0.008) of researchers reporting that the practice of automatically listing a senior member(s) of their department (including section chief or department head) as an author on all submitted articles was 'never justified' (Review item 3a 'Never justified').

Appendix Table A45. Moderators review item 3a. Moderators of justification **'Never justified'** for review item 2a, i.e., researchers reporting the practice of automatically listing a senior member(s) of their department (including section chief or department head) as an author on all submitted articles.

Question review item 3a: If so, do you feel this is justified? **Answer: 'Never justified'**

**Variable**	**Eligible (yes/no)**	**# of observations**	**Rationale for non-eligibility**	**Test of group differences***
Netherlands country of first affiliation	Yes	10		Q (1) = 6.68, p = 0.01**
Corresponding author (target population)	Yes	10		Q (1) = 7.05, p = 0.008**
≥ 50% males among surveyees	No	8	Less than 10 observations	
≥ 50% associate professor or higher among surveyees	No	5	Less than 10 observations	
Risk of multiple submissions by surveyees	No	6	Less than 10 observations	
Time frame ≤1 year between publication of manuscript and conducting the survey	No	9	Less than 10 observations	

*The chi squared test, 'Q', and the degrees of freedom (df), and the p value 'p'

**P<0.05

No significant associations were found through subgroup analyses between any of the tested explanatory variables and the results for researchers reporting that the practice of automatically listing a senior member(s) of their department (including section chief or department head) as an author on all submitted articles was 'rarely justified' (Review item 3a 'Rarely justified').

Appendix Table A46. Moderators review item 3a. Moderators of justification **'rarely justified'** for review item 2a, i.e., researchers reporting the practice of automatically listing a senior member(s) of their department (including section chief or department head) as an author on all submitted articles.

Question review item 3a: If so, do you feel this is justified? **Answer: 'Rarely justified'**

**Variable**	**Eligible (yes/no)**	**# of observations**	**Rationale for non-eligibility**	**Test of group differences***
Netherlands country of first affiliation	Yes	10		Q (1) = 1.00, p = 0.317
Corresponding author (target population)	Yes	10		Q (1) = 1.76, p = 0.185
≥ 50% males among surveyees	No	8	Less than 10 observations	
≥ 50% associate professor or higher among surveyees	No	5	Less than 10 observations	
Risk of multiple submissions by surveyees	No	6	Less than 10 observations	
Time frame ≤1 year between publication of manuscript and conducting the survey	No	9	Less than 10 observations	

*The chi squared test, 'Q', and the degrees of freedom (df), and the p value 'p'

Being a corresponding author was associated with a higher prevalence (Q (1) = 7.61, p = 0.006) of researchers reporting that the practice of automatically listing a senior member(s) of their department (including section chief or department head) as an author on all submitted articles was 'sometimes justified' (Review item 3a 'Sometimes justified').

Appendix Table A47. Moderators review item 3a. Moderators of justification **'Sometimes justified'** for review item 2a, i.e., researchers reporting the practice of automatically listing a senior member(s) of their department (including section chief or department head) as an author on all submitted articles.
Question review item 3a: If so, do you feel this is justified? **Answer: 'Sometimes justified'**

**Variable**	**Eligible (yes/no)**	**# of observations**	**Rationale for non-eligibility**	**Test of group differences***
Netherlands country of first affiliation	Yes	10		Q (1) = 0.52, p = 0.473
Corresponding author (target population)	Yes	10		Q (1) = 7.61, p = 0.006**
$\geq$ 50% males among surveyees	No	8	Less than 10 observations	
$\geq$ 50% associate professor or higher among surveyees	No	5	Less than 10 observations	
Risk of multiple submissions by surveyees	No	6	Less than 10 observations	
Time frame $\leq$1 year between publication of manuscript and conducting the survey	No	9	Less than 10 observations	

*The chi squared test, 'Q', and the degrees of freedom (df), and the p value 'p'
**P<0.05

When the survey was conducted by a research group whose' first author was affiliated with a research institute in the Netherlands it was more likely (Q (1) = 18.3, p < 0.01) that researchers reported that the practice of automatically listing a senior member(s) of their department (including section chief or department head) as an author on all submitted articles was 'most of the time justified' (Review item 3a 'Most of the time justified').

Appendix Table A48. Moderators review item 3a. Moderators of justification **'Most of the time justified'** for review item 2a, i.e., researchers reporting the practice of automatically listing a senior member(s) of their department (including section chief or department head) as an author on all submitted articles.

Question review item 3a: If so, do you feel this is justified? **Answer: 'Most of the time justified'**

Variable	Eligible (yes/no)	# of observations	Rationale for non-eligibility	Test of group differences*
Netherlands country of first affiliation	Yes	10		Q (1) = 18.3, p = 0.000**
Corresponding author (target population)	Yes	10		Q (1) = 1.95, p = 0.162
$\geq$ 50% males among surveyees	No	8	Less than 10 observations	
$\geq$ 50% associate professor or higher among surveyees	No	5	Less than 10 observations	
Risk of multiple submissions by surveyees	No	6	Less than 10 observations	
Time frame $\leq$1 year between publication of manuscript and conducting the survey	No	9	Less than 10 observations	

*The chi squared test, 'Q', and the degrees of freedom (df), and the p value 'p'

**P<0.05

No significant associations were found through subgroup analyses between any of the tested explanatory variables and the results for researchers reporting that the practice of automatically listing a senior member(s) of their department (including section chief or department head) as an author on all submitted articles was 'always justified' (Review item 3a 'Always justified').

Appendix Table A49. Moderators for review item 3a. Moderators of justification **'Always justified'** for review item 2a, i.e., researchers reporting the practice of automatically listing a senior member(s) of their department (including section chief or department head) as an author on all submitted articles.

Question review item 3a: If so, do you feel this is justified? **Answer: 'Always justified'**

Variable	Eligible (yes/no)	# of observations	Rationale for non-eligibility	Test of group differences*
Netherlands country of first affiliation	Yes	10		Q (1) = 3.86, p = 0.05
Corresponding author (target population)	Yes	10		Q (1) = 2.89, p = 0.09
$\geq$ 50% males among surveyees	No	8	Less than 10 observations	
$\geq$ 50% associate professor or higher among surveyees	No	5	Less than 10 observations	
Risk of multiple submissions by surveyees	No	6	Less than 10 observations	
Time frame $\leq$1 year between publication of manuscript and conducting the survey	No	9	Less than 10 observations	

*The chi squared test, 'Q', and the degrees of freedom (df), and the p value 'p'

Additional item O. Non-reporting biases

Non-reporting biases

Cochrane's 6-step framework to explore non-reporting biases in a synthesis [17].

Step 1. Select syntheses to assess for risk of bias due to missing results.

All outcomes that were meta-analysed were selected, i.e., the results to questions 2a, 2b, and 3a.

Step 2. Define which results are eligible for inclusion in each synthesis.

All results to questions 2a, 2b, and 3a were eligible and the non-reporting biases are presented Appendix Table A50.

Step 3. Record whether any of the studies identified are missing from each synthesis because results known (or presumed) to have been generated by study investigators are unavailable: the 'known unknowns'.

None of the included surveys had protocols registered or published a priori. We could therefore not compare the results reported in the published manuscripts with those planned in the protocols.

Step 4. Consider whether each synthesis is likely to be biased because of the missing results in the studies identified.

All 'known unknown' items were reported in Appendix Table A50.

Step 5. Consider whether results from additional studies are likely to be missing from each synthesis: the 'unknown unknowns'.

To avoid the risk of missing additional surveys, we had implemented various strategies such as the implementation of broad-spectrum search strategies and conducting searches in a wide body of data bases. However, additional studies could still have been missed during our searches. We did not assess small study effects through funnel plots, because there is no evidence available on the validity of these graphs for proportions [18].

Step 6. Reach an overall judgement about risk of bias due to missing results in each synthesis.

Our overall judgment is that the results to all questions, except one were at a moderate probability of risk of bias due to missing results in a synthesis (Appendix Table A50). Only Review item 3b, i.e., the justification of review item 2b, i.e., Researchers reporting the practice of automatically listing their section or department head as an author on all submitted articles was rated as high probability of risk of bias due to missing results, because the results of 2 [25,26] of the 3 surveys that addressed this review item were not given.

Appendix Table A50. Risk of non-reporting bias for review items

**Survey items and pertinent questions**	**Items that could have caused risk of bias due to missing results in a synthesis.**	**Overall judgment about risk of bias due to missing results in a synthesis**
**Review Item 2a.** Researchers reporting the practice of automatically listing a senior member(s) of their department (including section chief or department head) as an author on all submitted articles. **Question review item 2a:** Is there a senior member of your department (including section chief or department head) who is automatically listed as an author in all submitted manuscripts?	• Unknown unknowns', i.e., risk of missing additional surveys	Moderate probability of risk of bias due to missing results in a synthesis
**Review Item 2b.** Researchers reporting the practice of automatically listing their section or department head as an author on all submitted articles. **Question review item 2b:** Is your section or department head automatically listed as an author in all submitted manuscripts?	• Unknown unknowns', i.e., risk of missing additional surveys	Moderate probability of risk of bias due to missing results in a synthesis
**Review Item 2c.** Researchers reporting the practice of automatically listing a senior member(s) of their department, including their section chief or department head, as a co-author on a manuscript without fulfilling the ICMJE criteria for authorship. **Question review item 2c:** Is there a senior member of your department, including your section chief or department head, who is automatically listed as a coauthor in the review without fulfilling the ICMJE criteria for authorship?	• Unknown unknowns', i.e., risk of missing additional surveys	Moderate probability of risk of bias due to missing results in a synthesis

**Review Item 2d.** Researchers reporting the practice of automatically listing a senior member(s) of their department, including their section chief or department head, as a co-author(s) on all articles submitted by these researchers.<br>**Question review item 2d:**<br>Is there a senior member of your department, including your section chief or department head, who is automatically listed as a coauthor on all of your submitted manuscripts?	• Unknown unknowns', i.e., risk of missing additional surveys	Moderate probability of risk of bias due to missing results in a synthesis
**Review item 3a**. Justification of review item 2a, i.e., Researchers reporting the practice of automatically listing a senior member(s) of their department (including section chief or department head) as an author on all submitted articles.<br>**Question review item 3a:**<br>If so, do you feel this is justified?	• Unknown unknowns', i.e., risk of missing additional surveys	Moderate probability of risk of bias due to missing results in a synthesis
**Review item 3b**.<br>Justification of review item 2b, i.e., Researchers reporting the practice of automatically listing their section or department head as an author on all submitted articles.<br>**Question review item 3b:**<br>If so, do you feel that this is justified in all cases?	• Unknown unknowns', i.e., risk of missing additional surveys	High probability of risk of bias due to missing results in a synthesis<br><br>This outcome was assessed in 2 other surveys [25,26], but results were not published.
**Review item 3d**.<br>Justification of review item 2d, i.e., Researchers reporting the practice of automatically listing a senior member(s) of their department, including their section chief or department head, as a co-author(s) on all articles submitted by these researchers.<br>**Question review item 3d:**<br>If yes, do you feel that this is justified in all cases?	• Unknown unknowns', i.e., risk of missing additional surveys	Moderate probability of risk of bias due to missing results in a synthesis

Additional item P. Certainty of the evidence

Appendix Table A51. Summary of findings. Prevalence of issues regarding the practice of automatically listing senior members as co-authors on submitted articles

**Surveyee:** Any author on the author list of a scientific publication, e.g., first, last, corresponding author, that was invited to participate in a survey on at least one of our review items. **Settings:** Any. **Intervention:** Surveys based on questionnaires for self-completion				
**Survey items**	**Prevalence (95% CI)**	**# of respondents and surveys**	**Certainty of the evidence (GRADE)**	**Comments**
**Review Item 2a (Question 2a).** Researchers reporting the practice of automatically listing a senior member(s) of their department (including section chief or department head) as an author on all submitted articles.	20% [95% CI 16-25]	3,619 respondents in 10 surveys	⊕⊝⊝⊝ **Very low**[a] Due to risk of bias, inconsistency, imprecision, and moderate risk of non-reporting biases	Evidence from 10 surveys
**Review Item 2b (Question 2b).** Researchers reporting the practice of automatically listing their section or department head as an author on all submitted articles.	25% [95% CI 22-27]	1,020 respondents in 3 surveys	⊕⊝⊝⊝ **Low**[b] Due to risk of bias, and moderate risk of non-reporting biases	Evidence from 3surveys
**Review Item 2c (Question 2c).** Researchers reporting the practice of automatically listing a senior member(s) of their department, including their section chief or department head, as a co-author on a manuscript without fulfilling the ICMJE criteria for authorship.	6.8% (45/666) [95% CI 5-8.9]	666 respondents in 1 survey [22]	⊕⊕⊝⊝ **low**[c] Due to risk of bias, imprecision, and moderate risk of non-reporting biases	Evidence from 1 survey [22]
**Review Item 2d (Question 2d).** Researchers reporting the practice of automatically listing a senior member(s) of their department, including their section chief or department head, as a co-author(s) on all articles submitted by these researchers.	12.6% (31/247) [95% CI 8.7-17.4]	247 respondents in 1 survey [23]	⊕⊝⊝⊝ **Very low**[d] Due to risk of bias, imprecision, and moderate risk of non-reporting biases	Evidence from 1 survey [23]
**Review item 3a (Question 3a)**. Justification **'Never justified'** for review item 2a, i.e., researchers reporting the practice of automatically listing a senior member(s) of their department (including section chief or department head) as an author on all submitted articles.	28% [95% CI 22-34]	2,180 respondents in 10 surveys	⊕⊝⊝⊝ **Very low**[e] Due to risk of bias, inconsistency, imprecision, and moderate risk of non-reporting biases	Evidence from 10 surveys
**Review item 3a (Question 3a)**. Justification **'Rarely justified'** for review item 2a, i.e., researchers reporting the practice of automatically listing a senior member(s) of their department (including section chief or department head) as an author on all submitted articles.	24% [95% CI 22-27]	2,180 respondents in 10 surveys	⊕⊕⊝⊝ **Low**[f] Due to risk of bias and moderate risk of non-reporting biases	Evidence from 10 surveys
**Review item 3a (Question 3a)**. Justification **'Sometimes justified'** for review item 2a, i.e., researchers reporting the practice of automatically listing a senior member(s) of their department (including section chief or department head) as an author on all submitted articles.	25% [95% CI 23-28]	2,180 respondents in 10 surveys	⊕⊕⊝⊝ **Low**[g] Due to risk of bias and moderate risk of non-reporting biases	Evidence from 10 surveys
**Review item 3a (Question 3a)**. Justification **'Most of the time justified'** for review item 2a, i.e., researchers reporting the practice of automatically listing a senior member(s) of their department (including section chief or department head) as an author on all submitted articles.	13% [95% CI 9-17]	2,180 respondents in 10 surveys	⊕⊝⊝⊝ **Very low**[h] Due to risk of bias, inconsistency, imprecision, and moderate risk of non-reporting biases	Evidence from 10 surveys
**Review item 3a (Question 3a)**. Justification **'Always justified'** for review item 2a, i.e., researchers reporting the practice of automatically listing a senior member(s) of their department (including section chief or department head) as an author on all submitted articles.	8% [95% CI 6-9]	2,180 respondents in 10 surveys	⊕⊝⊝⊝ **Very low**[i] Due to risk of bias, inconsistency, imprecision, and moderate risk of non-reporting biases	Evidence from 10 surveys
**Review item 3b (Question 3b)**. Justification **'Justified in all cases'** for review item 2b, i.e., Researchers reporting the practice of automatically listing their section or department head as an author on all submitted articles.	35.4% (34/96) [95% CI 25.9-45.8]	96 respondents in 1 survey) [24]	⊕⊝⊝⊝ **Very low**[j] Due to risk of bias, imprecision, and high risk of non-reporting biases	Evidence from 1 survey [24]**.** For this outcome the results of 2 surveys [25,26] were not available
**Review item 3d. (Question 3d)** Justification **'Justified in all cases'** for review item 2d, i.e., Researchers reporting the practice of automatically listing a senior member(s) of their department, including their section chief or department head, as a co-author(s) on all articles submitted by these researchers.	67.7% (21/31) [95% CI 48.6-83.3]	31 respondents in 1 survey [23]	⊕⊝⊝⊝ **Very low**[k] Due to risk of bias, imprecision, and non-reporting biases	Evidence from 1 survey [23]

Explanations

[a]The risk of bias was high for this outcome, because the overall confidence in the results of the 10 included surveys was rated as either low or critically low. The low P value and large Chi^2 ($Chi^2 = 95.84$ (df = 9) P<0.001) provide evidence of heterogeneity and the high I^2 ($I^2 = 90.61\%$) indicates considerable inconsistency across the prevalence statistics of the surveys. The wide confidence intervals indicate imprecision and there was moderate probability of non-reporting biases (Appendix Figure A2).

[b]The risk of bias was high for this outcome, because the overall confidence in the results of the 3 included surveys was rated as low or critically low. There was moderate probability of non-reporting biases.

[c]The risk of bias was high for this outcome, because the overall confidence in the results of the survey was rated as low. Imprecision was present, i.e., small number of studies (or single study) and/or a small number of participants (per study). There was moderate probability of non-reporting biases.

[d] The risk of bias was high for this outcome, because the overall confidence in the results of the survey was rated as critically low. Imprecision was present, i.e., small number of studies (or single study) and/or a small number of participants (per study). There was moderate probability of non-reporting biases.

[e]The risk of bias was high for this outcome, because the overall confidence in the results of the 10 included surveys was rated as either low or critically low. The low P value and large Chi^2 ($Chi^2 = 88.89$ (df = 9) P<0.001) provide evidence of heterogeneity and the high I^2 ($I^2 = 89.88\%$) indicates considerable inconsistency across the prevalence statistics of the surveys. The wide confidence intervals indicate imprecision and there was moderate probability of non-reporting biases (Appendix Figure A3).

[f]The risk of bias was high for this outcome, because the overall confidence in the results of the 10 included surveys was rated as either low or critically low and there was moderate probability of non-reporting biases.

[g]The risk of bias was high for this outcome, because the overall confidence in the results of the 10 included surveys was rated as low or critically low and there was moderate probability of non-reporting biases.

[h]The risk of bias was high for this outcome, because the overall confidence in the results of the 10 included surveys was rated as low or critically low. The low P value and large Chi^2 ($Chi^2 = 71.49$ (df = 9) P<0.001) provide evidence of heterogeneity and the high I^2 ($I^2 = 87.41\%$) indicates considerable inconsistency across the prevalence statistics of the surveys. The wide confidence intervals indicate imprecision and there was moderate probability of non-reporting biases (Appendix Figure A6).

[i]The risk of bias was high for this outcome, because the overall confidence in the results of the 10 included surveys was rated as low or critically low. The low P value and large Chi^2 ($Chi^2 = 25.08$ (df = 9) P<0.001) provide evidence of heterogeneity and the high I^2 ($I^2 = 64.12\%$) indicates considerable inconsistency across the prevalence statistics of the surveys. The wide confidence intervals indicate imprecision and there was moderate probability of non-reporting biases (Appendix Figure A7).

[j]The risk of bias was high for this outcome, because the overall confidence in the results of the survey was rated as low. Imprecision was present, i.e., small number of studies (or single study) and/or a small number of participants (per study). There was high probability of non-reporting biases, because the results of 2 surveys [25,26] were not reported. The corresponding author responded by email that additional data beyond what was published were not available anymore.

[k]The risk of bias was high for this outcome, because the overall confidence in the results of the survey was rated as critically low. Imprecision was present, i.e., small number of studies (or single study) and/or a small number of participants (per study). There was moderate probability of non-reporting biases.

Additional item Q. References

1. Meursinge Reynders R, Ter Riet G, Di Girolamo N, Cavagnetto D, Malički M. Honorary authorship is highly prevalent in health sciences: systematic review and meta-analysis of surveys. September 23 2023, PREPRINT (Version 1) available at Research Square [https://doi.org/10.21203/rs.3.rs-3321504/v1].
2. Meursinge Reynders R, Ter Riet G, Di Girolamo N, Malički M. Honorary authorship in health sciences: a protocol for a systematic review of survey research. Syst Rev. 2022 Apr 4;11(1):57. doi: 10.1186/s13643-022-01928-1. PMID: 35379330; PMCID: PMC8978359.
3. Wikipedia Survey. [online] Available from: https://en.wikipedia.org/wiki/Survey_(human_research) (accessed August 10th 2023).
4. Wikipedia Health sciences. [online] Available from: https://en.wikipedia.org/wiki/Outline_of_health_sciences (accessed August 10th 2023).
5. International Committee of Medical Journal Editors (ICMJE). Recommendations for the Conduct, Reporting, Editing, and Publication of Scholarly Work in Medical Journals. Updated May 2023. [online] Available from: http://www.icmje.org/icmje-recommendations.pdf (accessed August10th 2023).
6. Deeks JJ, Higgins JPT, Altman DG (editors). Chapter 10: Analysing data and undertaking meta-analyses. In: Higgins JPT, Thomas J, Chandler J, Cumpston M, Li T, Page MJ, Welch VA (editors). Cochrane Handbook for Systematic Reviews of Interventions version 6.2 (updated February 2021). Cochrane, 2021. Available from www.training.cochrane.org/handbook.
7. Röver C, Friede T. Double arcsine transform not appropriate for meta-analysis. Res Synth Methods. 2022 Sep;13(5):645-648. doi: 10.1002/jrsm.1591. Epub 2022 Jul 22. PMID: 35837800.
8. Schwarzer G, Chemaitelly H, Abu-Raddad LJ, Rücker G. Seriously misleading results using inverse of Freeman-Tukey double arcsine transformation in meta-analysis of single proportions. Res

Synth Methods. 2019 Sep;10(3):476-483. doi: 10.1002/jrsm.1348. Epub 2019 Apr 23. PMID: 30945438; PMCID: PMC6767151

9. Van Epps H, Astudillo O, del Pozo Martín Y, Marsh J. The Sex and Gender Equity in Research (SAGER) guidelines: Implementation and checklist development. Eur Sci Ed. 2022;48:e86910. https://doi.org/10.3897/ese.2022.e86910.
10. Bethlehem J. Chapter 12: A checklist for polls. In 'Understanding Public Opinion Polls'. Boca Raton, Florida (USA): Chapman and Hall/CRC; 2017. ISBN: 978-1498769747.
11. Shea BJ, Reeves BC, Wells G, Thuku M, Hamel C, Moran J, Moher D, Tugwell P, Welch V, Kristjansson E, Henry DA. AMSTAR 2: a critical appraisal tool for systematic reviews that include randomised or non randomised studies of healthcare interventions, or both. BMJ. 2017 Sep 21;358:j4008. doi: 10.1136/bmj.j4008.
12. Sergeant ESG. Sergeant, ESG, 2018. Epitools Epidemiological Calculators. Ausvet. [online] Available from: http://epitools.ausvet.com.au. (accessed August 10th 2023).
13. StataCorp. 2023. *Stata Statistical Software: Release 18*. College Station, TX: StataCorp LLC.
14. McKenzie JE, Brennan SE, Ryan RE, Thomson HJ, Johnston RV. Chapter 9: Summarizing study characteristics and preparing for synthesis. In: Higgins JPT, Thomas J, Chandler J, Cumpston M, Li T, Page MJ, Welch VA (editors). Cochrane Handbook for Systematic Reviews of Interventions version 6.2 (updated February 2021). Cochrane, 2021. Available from www.training.cochrane.org/handbook.
15. McKenzie JE, Brennan SE. Chapter 12: Synthesizing and presenting findings using other methods. In: Higgins JPT, Thomas J, Chandler J, Cumpston M, Li T, Page MJ, Welch VA (editors). Cochrane Handbook for Systematic Reviews of Interventions version 6.2 (updated February 2021). Cochrane, 2021. Available from www.training.cochrane.org/handbook.

16. Borenstein M, Hedges LV, Higgins JPT, Rothstein HR: Chapter 20: Meta-regression. In Introduction to Meta-Analysis. Edited by Borenstein M, Hedges LV, Higgins JPT, Rothstein HR. Chichester (UK): John Wiley & Sons; 2009.
17. Page MJ, Higgins JPT, Sterne JAC. Chapter 13: Assessing risk of bias due to missing results in a synthesis. In: Higgins JPT, Thomas J, Chandler J, Cumpston M, Li T, Page MJ, Welch VA (editors). *Cochrane Handbook for Systematic Reviews of Interventions* version 6.3 (updated February 2022). Cochrane, 2022. Available from www.training.cochrane.org/handbook.
18. Barker TH, Migliavaca CB, Stein C, Colpani V, Falavigna M, Aromataris E, Munn Z. Conducting proportional meta-analysis in different types of systematic reviews: a guide for synthesisers of evidence. BMC Med Res Methodol. 2021 Sep 20;21(1):189. doi: 10.1186/s12874-021-01381-z. PMID: 34544368; PMCID: PMC8451728.
19. Schünemann HJ, Higgins JPT, Vist GE, Glasziou P, Akl EA, Skoetz N, Guyatt GH. Chapter 14: Completing 'Summary of findings' tables and grading the certainty of the evidence. In: Higgins JPT, Thomas J, Chandler J, Cumpston M, Li T, Page MJ, Welch VA (editors). Cochrane Handbook for Systematic Reviews of Interventions version 6.2 (updated February 2021). Cochrane, 2021. Available from www.training.cochrane.org/handbook.
20. Page MJ, McKenzie JE, Bossuyt PM, Boutron I, Hoffmann TC, Mulrow CD, Shamseer L, Tetzlaff JM, Akl EA, Brennan SE, Chou R, Glanville J, Grimshaw JM, Hróbjartsson A, Lalu MM, Li T, Loder EW, Mayo-Wilson E, McDonald S, McGuinness LA, Stewart LA, Thomas J, Tricco AC, Welch VA, Whiting P, Moher D. The PRISMA 2020 statement: an updated guideline for reporting systematic reviews. BMJ. 2021 Mar 29;372:n71. doi: 10.1136/bmj.n71. PMID: 33782057; PMCID: PMC8005924.
21. Page MJ, Moher D, Bossuyt PM, Boutron I, Hoffmann TC, Mulrow CD, Shamseer L, Tetzlaff JM, Akl EA, Brennan SE, Chou R, Glanville J, Grimshaw JM, Hróbjartsson A, Lalu MM, Li T, Loder EW,

Mayo-Wilson E, McDonald S, McGuinness LA, Stewart LA, Thomas J, Tricco AC, Welch VA, Whiting P, McKenzie JE. PRISMA 2020 explanation and elaboration: updated guidance and exemplars for reporting systematic reviews. BMJ. 2021 Mar 29;372:n160. doi: 10.1136/bmj.n160. PMID: 33781993; PMCID: PMC8005925.

22. Gülen S, Fonnes S, Andresen K, Rosenberg J. More than one-third of Cochrane reviews had gift authors, whereas ghost authorship was rare. J Clin Epidemiol. 2020 Dec;128:13-19. doi: 10.1016/j.jclinepi.2020.08.004. Epub 2020 Aug 8. PMID: 32781115.
23. Rajasekaran S, Shan RL, Finnoff JT. Honorary authorship: frequency and associated factors in physical medicine and rehabilitation research articles. Arch Phys Med Rehabil. 2014;95(3):418–428. doi:10.1016/j.apmr.2013.09.024
24. Eisenberg RL, Ngo L, Boiselle PM, Bankier AA. Honorary authorship in radiologic research articles: assessment of frequency and associated factors. Radiology. 2011 May;259(2):479-86. doi: 10.1148/radiol.11101500. Epub 2011 Mar 8. PMID: 21386051.
25. Eisenberg RL, Ngo LH, Bankier AA. Honorary authorship in radiologic research articles: do geographic factors influence the frequency? Radiology. 2014 May;271(2):472-8. doi: 10.1148/radiol.13131710. Epub 2013 Nov 27. PMID: 24475845.
26. Eisenberg RL, Ngo LH, Heidinger BH, Bankier AA. Honorary Authorship in Radiologic Research Articles: Assessment of Pattern and Longitudinal Evolution. Acad Radiol. 2018;25(11):1451–1456. doi:10.1016/j.acra.2018.02.023.
